# Spatial and temporal dynamics of Antarctic shallow soft-bottom benthic communities: ecological drivers under climate change

**DOI:** 10.1186/s12898-019-0244-x

**Published:** 2019-07-01

**Authors:** Belinda J. Vause, Simon A. Morley, Vera G. Fonseca, Anna Jażdżewska, Gail V. Ashton, David K. A. Barnes, Hendrik Giebner, Melody S. Clark, Lloyd S. Peck

**Affiliations:** 1 British Antarctic Survey, High Cross, Madingley Road, Cambridge, Cambridgeshire CB30ET UK; 2 Centre for Molecular Biodiversity Research, Zoological Research Museum Alexander Koenig (ZFMK), Adenauerallee 160, 53113 Bonn, Germany; 3 Laboratory of Polar Biology and Oceanobiology, Department of Invertebrate Zoology and Hydrobiology, Faculty of Biology and Environmental Protection, University of Lodz, 12/16 Banacha st., 90-237 Lodz, Poland; 4 Smithsonian Environmental Research Center, Romberg Tiburon Center, Tiburon, CA USA

**Keywords:** Disturbance ecology, Patchiness, Seasonality, Latitudinal comparisons, Sediment properties

## Abstract

**Background:**

Marine soft sediments are some of the most widespread habitats in the ocean, playing a vital role in global carbon cycling, but are amongst the least studied with regard to species composition and ecosystem functioning. This is particularly true of the Polar Regions, which are currently undergoing rapid climate change, the impacts of which are poorly understood. Compared to other latitudes, Polar sediment habitats also experience additional environmental drivers of strong seasonality and intense disturbance from iceberg scouring, which are major structural forces for hard substratum communities. This study compared sediment assemblages from two coves, near Rothera Point, Antarctic Peninsula, 67°S in order to understand the principal drivers of community structure, for the first time, evaluating composition across all size classes from mega- to micro-fauna.

**Results:**

Morpho-taxonomy identified 77 macrofaunal species with densities of 464–16,084 individuals m^−2^. eDNA metabarcoding of microfauna, in summer only, identified a higher diversity, 189 metazoan amplicon sequence variants (ASVs) using the 18S ribosomal RNA and 249 metazoan ASVs using the mitochondrial COI gene. Both techniques recorded a greater taxonomic diversity in South Cove than Hangar Cove, with differences in communities between the coves, although the main taxonomic drivers varied between techniques. Morphotaxonomy identified the main differences between coves as the mollusc, *Altenaeum charcoti*, the cnidarian *Edwardsia* sp. and the polychaetes from the family cirratulidae. Metabarcoding identified greater numbers of species of nematodes, crustaceans and Platyhelminthes in South Cove, but more bivalve species in Hangar Cove. There were no detectable differences in community composition, measured through morphotaxonomy, between seasons, years or due to iceberg disturbance.

**Conclusions:**

This study found that unlike hard substratum communities the diversity of Antarctic soft sediment communities is correlated with the same factors as other latitudes. Diversity was significantly correlated with grain size and organic content, not iceberg scour. The increase in glacial sediment input as glaciers melt, may therefore be more important than increased iceberg disturbance.

**Electronic supplementary material:**

The online version of this article (10.1186/s12898-019-0244-x) contains supplementary material, which is available to authorized users.

## Background

Over 70% of the earth’s surface is ocean, with the vast majority of these sea floors covered in sediments. Thus, marine soft sediment communities are possibly the largest ecotype, and are arguably one of the dominant components of the earth’s biota. They also contribute significantly to marine ecosystem functioning [1], being key components of energy flow through food webs. They play important roles in sedimentary processes especially nutrient and carbon cycling, waste breakdown and removal [2]. In spite of this recognised importance, there are some areas of the globe where our knowledge of such communities, even in terms of the species living there, is poor. In these regions our confidence of predictions of future change is either low or not possible due to insufficient data [3]. This is particularly true of the polar regions [4]. However such knowledge is critical, given that some of these areas, in particular the Arctic and Antarctic Peninsula, have experienced some of the most rapid rates of regional climate change [3]. Comprehensive base-line studies are required in these regions, which evaluate not only current levels of biodiversity across all organism size classes, but also the underlying ecological drivers that shape these communities. At the moment there is very little understanding as to whether the drivers that shape sediment communities in temperate and tropical regions are the same at the poles or, whether, the “extra” contributing ecological factors in the poles, which are currently changing, will impact soft sediment communities in the future.

In spite of indications that warming of the air may have ceased along the Peninsula [5], the marine environment is still in transition, with continued reductions in annual sea ice duration, deglaciation and ice shelf collapse, which can significantly impact the endemic fauna [6, 7]. Increased melt water from glaciers can lead to an increase in the sediment load entering coastal waters from glacial grinding of bedrock, so called glacial flour, which will result in increased sedimentation rates onto the sea floor, potentially smothering benthic communities [8]. Globally, a wide range of linked physiological and biological factors determines sediment community structure, both in terms of abundance and taxonomic composition. Of these, sediment grain size is often the key physical parameter as it strongly correlates with many other factors, such as porosity, oxygen and organic content [9, 10]. Organisms in Antarctic sediment communities have to cope with the additional structuring elements of intense seasonality and ice. The Southern Ocean is subject to higher levels of solar irradiance in summer compared to other regions of the planet due to the Earth’s tilt [11]. This drives the extremely high phytoplankton blooms (and food supply) in the summer, followed by virtual absence in the winter [12]. Decreased winter sea ice along the Peninsula is both increasing the amount of light reaching coastal benthic ecosystems and also altering water column stratification. These factors are changing the time of onset, and strength of, the following summer bloom [13, 14]. Reduced winter fast ice is also leading to an increase in the frequency of ice-berg scouring which has significant impacts on community structure at depths shallower than 30 m [15, 16]. Hence it is essential to understand how these additional factors further structure marine sediment communities and are likely to affect future shallow water benthic biodiversity.

Until recently the few previous analyses of soft sediment communities in the Antarctic have concentrated on morpho-taxonomy of macro species [17]. Even so, these have shown considerable levels of biodiversity, similar to that of temperate regions. Thus indicating that the general paradigm of increasing biodiversity from the poles to the tropics only holds for the Northern hemisphere, not the more evolutionary and geographically isolated Southern hemisphere [12]. Recent analyses on sediment meiofauna have shown similar high levels of biodiversity [18], but also that these communities respond differently to change when compared with the macrofauna [19–21]. These latter studies have concentrated on colonization processes after ice shelf and glacier collapse, which analyse community progressions from extremely food-poor environments [20–22]. They may not accurately represent the responses, or rate of response, due to the inexorable consequences of a decrease in the duration of winter sea ice or gradual warming. Studies monitoring hard substrata communities in the bays around Rothera research base since 1997 have shown significant changes in species’ composition and structure over 7 years, which can be directly related to increased ice berg activity [23]. However, we currently have very few data, not only on how Antarctic soft sediment communities might be affected in the future, but also on the rate at which these communities respond to change, despite this being the dominant benthic habitat. This study provides data to fill this gap.

Here, we present the first study to comprehensively analyse the composition of Antarctic shallow water benthic sediment communities at two sites replicated sampling over 2.5 years. Fauna were catalogued across all size ranges from the mega- (10 cm–5 mm), macro- (5–1 mm) through to the micro-fauna (< 1 mm). A combination of morpho-taxonomy and metabarcoding approaches were used to evaluate levels of biodiversity in two adjacent coves, with different levels of exposure to iceberg scour [15] near Rothera Point on the Antarctic Peninsula. The choice of sites and sampling regime enabled, not only the assessment of levels of biodiversity across the different size classes, but also the dissection of the drivers behind sediment community composition in this extreme environment and to identify whether they differed to those of temperate and tropical regions.

## Results

### Morpho-taxonomy of mega- and macro-fauna

A total of 55,536 individuals were identified from 8 phyla, 14 classes, 37 orders, 62 families. Within these families, 72 genera and 77 species could be distinguished, with most families represented in 3 major phyla (Additional file 1: Table S1). Densities of individuals ranged from 464 to 16,084 individuals m^−2^.

There was no evidence of a temporal, i.e. seasonal, effect on biodiversity. There were no significant differences found in benthic assemblages within each cove between years (one-way ANOSIM, global *R *= 0.4 *P *= 0.18) or seasons (global *R *= 0.04 *P *= 0.14).

**Fig. 1:**
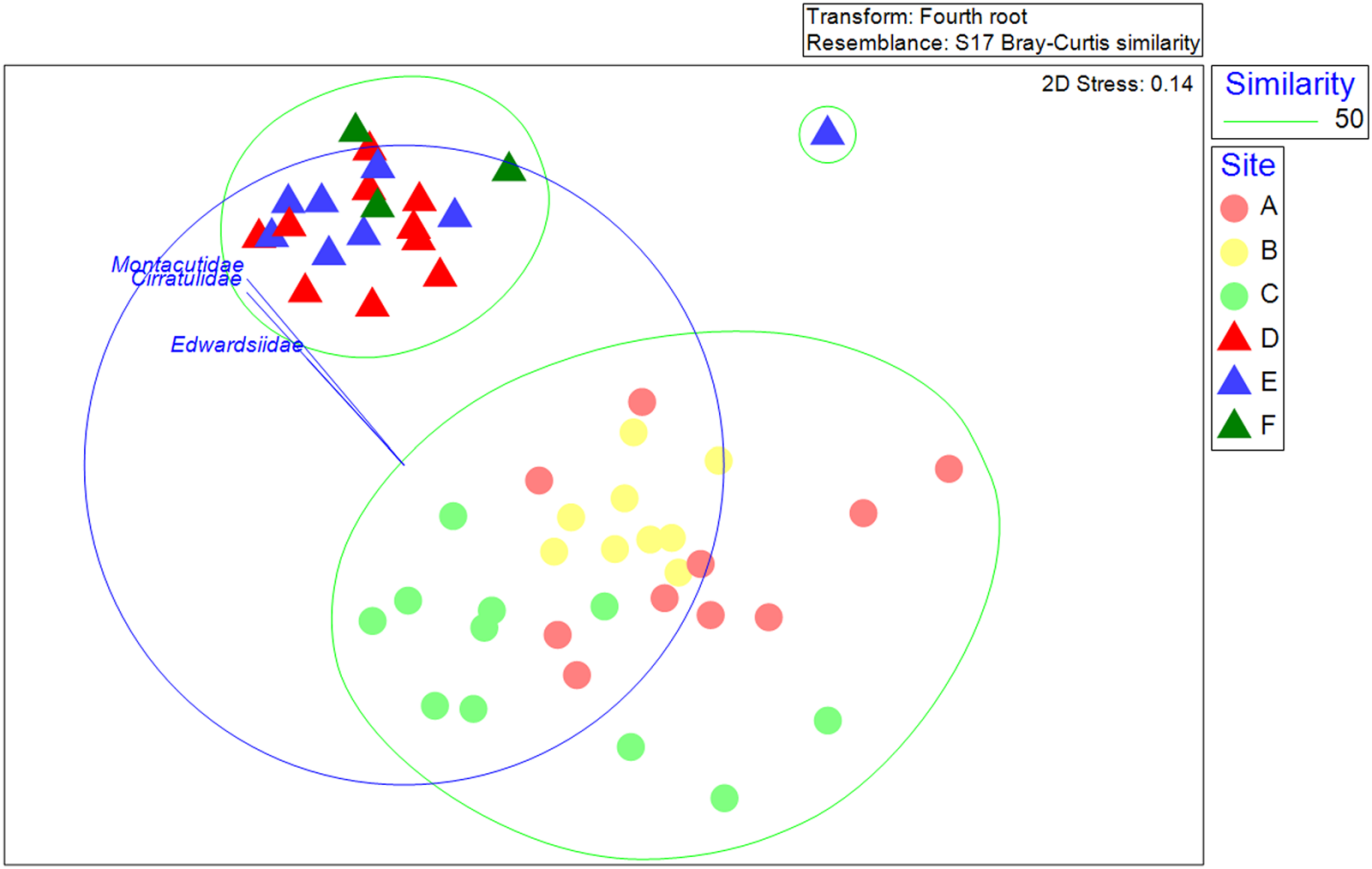
Non-metric multidimensional scaling (nMDS) plots based on the Bray–Curtis similarity matrix for the abundance of all families sampled. Benthic assemblages at South Cove as denoted by circles (Sites A, B and C) and Hangar Cove by triangles (Sites D, E and F). The low stress value of 0.14 indicates this plot is a good two dimensional representation of the community in multi-dimensional space. Clustering is shown at a similarity level of 50%

However, the relative community composition in each cove was different. The abundance of individuals (*N*) was significantly higher in Hangar than South Cove (Table 1) (*R*^2^= 0.84, ANOVA *F*_(1,5)_= 20.7, *P* < 0.01). Species richness (*d*), evenness (*J*′) and diversity (*H*′) were significantly higher in South Cove (all *R*^2^ > 0.8, ANOVA *F*_(1,5)_ > 22, *P *< 0.01) while species dominance (*D*) was significantly lower in South Cove (*R*^*2*^ > 0.79, ANOVA *F*_(1,5)_ = 15.0, *P *< 0.02). Benthic assemblages from the two coves were clearly separated from each other and samples within Hangar Cove were clustered more closely than those from South Cove (Bray–Curtis similarity, Fig. 1) except for one sample from Hangar Cove, site E, sampled on 29/04/13, which was clearly separated as an outlier (Fig. 1; Additional file 2). There was a significant difference in the benthic assemblage of families from all phyla between the coves (one-way ANOSIM; *R *= 0.88 *P *< 0.001). A two-way nested ANOSIM revealed a highly significant but small (low *R* value) difference between sites within a cove (global *R *= 0.25 *P *< 0.001).

**Table 1:** Number of samples (*n*), Total number of families (*S*), Mean number of individuals in 0.25 m^2^ (*N*), Margalef’s index of Species richness (*d*), Pielou’s evenness (*J*′), Shannon diversity index (*H*′), Simpson dominance index (*D*) and associated standard deviations from each site studied

Site/Cove	*n*	*S*	*N*	*d*	*J*′	*H*′(*loge*)	*D*
South Cove	30	51	349.6 ± 213	3.27 ± 0.58	0.67 ± 0.09	1.97 ± 0.26	0.22 ± 0.07
A	10	41	246.6 ± 160.33	3.47 ± 0.5	0.71 ± 0.08	2.11 ± 0.25	0.19 ± 0.07
B	9	33	432.2 ± 180.83	3.17 ± 0.45	0.61 ± 0.09	1.83 ± 0.29	0.26 ± 0.08
C	11	45	375.7 ± 254.5	3.18 ± 0.2	0.67 ± 0.07	1.95 ± 0.19	0.21 ± 0.04
Hangar Cove	21	49	2145.1 ± 1118.36	2.73 ± 0.36	0.46 ± 0.12	1.41 ± 0.34	0.41 ± 0.14
D	10	39	2448.6 ± 1196.06	2.75 ± 0.38	0.47 ± 0.12	1.44 ± 0.37	0.39 ± 0.15
E	8	41	2089 ± 1071.18	2.7 ± 0.41	0.44 ± 0.13	1.32 ± 0.33	0.47 ± 0.14
F	3	28	1283 ± 682.44	2.77 ± 0.26	0.52 ± 0.07	1.57 ± 0.23	0.33 ± 0.07

The average dissimilarity between Hangar Cove and South Cove was 53.7% (SIMPER). These differences were largely due to the dominance of the Molluscan bivalve family Montacutidae [represented by only one species, *Altenaeum charcoti* (previously *Mysella charcoti*)], which accounted for 13.2% of the total dissimilarity, the Cnidarian family Edwardsiidae (represented by only one genus *Edwardsia*) at 6.6% and the Polychaete family Cirratulidae at 5.2%.

The average similarity between samples within Hangar Cove was 70.5% with infaunal bivalves accounting for more than a quarter of the total similarity due to the abundance of Montacutidae (*Altenaeum charcoti*), which accounted for 16% of the total and Yoldiidae (represented by only one species *Aequiyoldia eightsi*) at 10.6%. The polychaete families Cirratulidae (9.5%) and Orbiniidae (8.5%) were also major contributors. The average similarity within South Cove was slightly less at 62.6%, with the main contributors being the polychaete family Orbiniidae (13.5%), the echinoderm family Ophiuridae (represented by only one species *Ophionotus victoriae*) (11.6%), the mollusc family Yoldiidae (*Aequiyoldia eightsi*) (10.9%) and the amphipod family Oedicerotidae, which comprised 10.2% of the total.

To check for taxon surrogacy, further analysis was conducted at the genus level of one Phyla, the Mollusca. A one-way ANOSIM test revealed a significant difference in Molluscan genera assemblages between the coves (global *R *= 0.78 *P *< 0.01, see Additional file 2: Fig. S1). The separation of the benthic assemblage was almost identical at this finer taxonomic scale (genus level).

### Functional diversity

Comparisons (ANOSIM) for all three functional traits tested (mobility, lifestyle and size), within the macro and mega-faunal assemblages, found significant difference between the coves. South Cove had more epifauna than Hangar Cove (epifauna *R *= 0.717, *P* < 0.001), while Hangar Cove had more infauna (infauna *R *= 0.809 *P* < 0.001). South Cove fauna were more mobile (errant *R *= 0.771 *P *< 0.001). South Cove also had a higher proportion of megafaunal species (megafauna *R *= 0.345, *P* < 0.001, macrofauna *R *= 0.925, *P* < 0.001).

#### Iceberg impact

Five of the 51 macro- and megafaunal samples were collected from seabed that had been scoured by icebergs within 20 weeks of sampling (South Cove site C within 80 days; South Cove site A within 134 days and Hangar Cove site F, was sampled within 7, 10 and 39 days). Two-way crossed (Iceberg impact/no impact × Cove) ANOSIM revealed no significant difference in benthic assemblage found in these samples compared to samples with no visible evidence of recent impact (global *R *= 0.202 *P *= 0.1).

### Metabarcoding of species smaller than 1 mm (microfauna)

**Fig. 2:**
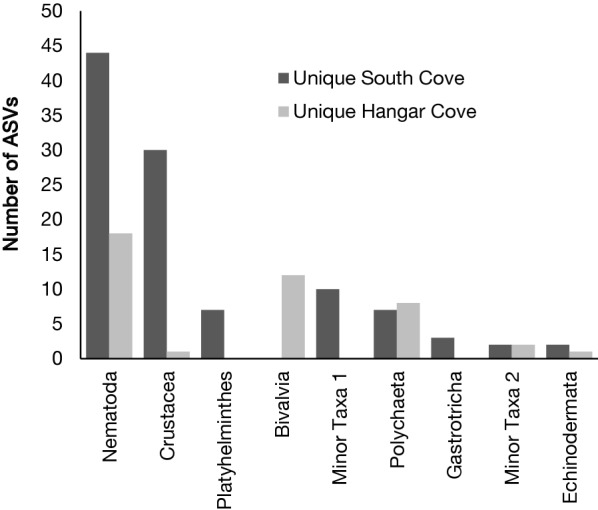
Number of unique amplicon sequence variants (ASVs) for each metazoan taxon found by metabarcoding in the two sampling locations, South and Hangar Coves in the Antarctic Peninsula. ASVs were retrieved using 18S rRNA gene region with blast matches higher than 95% sequence similarity against the SILVA database. Minor taxon 1 = Rotifera, Scalidophora, Xenacoelomorpha, Nemertea and Acanthocephala. Minor taxon 2 = Gnathostomulida, Hemichordata and Hydrozoa

The total number of ASVs assigned to Metazoan for the 18S rRNA and COI, was 189 and 249 respectively. Taxonomic composition using the 18S rRNA showed that the benthos is dominated by the nematodes, followed by Arthropods (namely crustaceans), Platyhelminthes and bivalves (Fig. 2; Additional file 3: Table S4). The biggest proportional differences in ASVs were for crustaceans (19%) and platyhelminthes (4%) that were more abundant in South Cove and mollusca (19%) which were more abundant in Hangar Cove.

The frequency of shared versus unique ASVs showed that ca. 80% of ASVs were unique to one or other cove with only 20% of ASVs common to both coves. The majority of ASVs (1365) were assigned to non-metazoan phyla (Heterokonta, Alveolates, Rhizaria, together referred to as “SAR”). Taxonomic composition from the COI was mainly represented by Arthropods (49%) and Annelida (15%) (Additional file 4: Table S5).

**Fig. 3:**
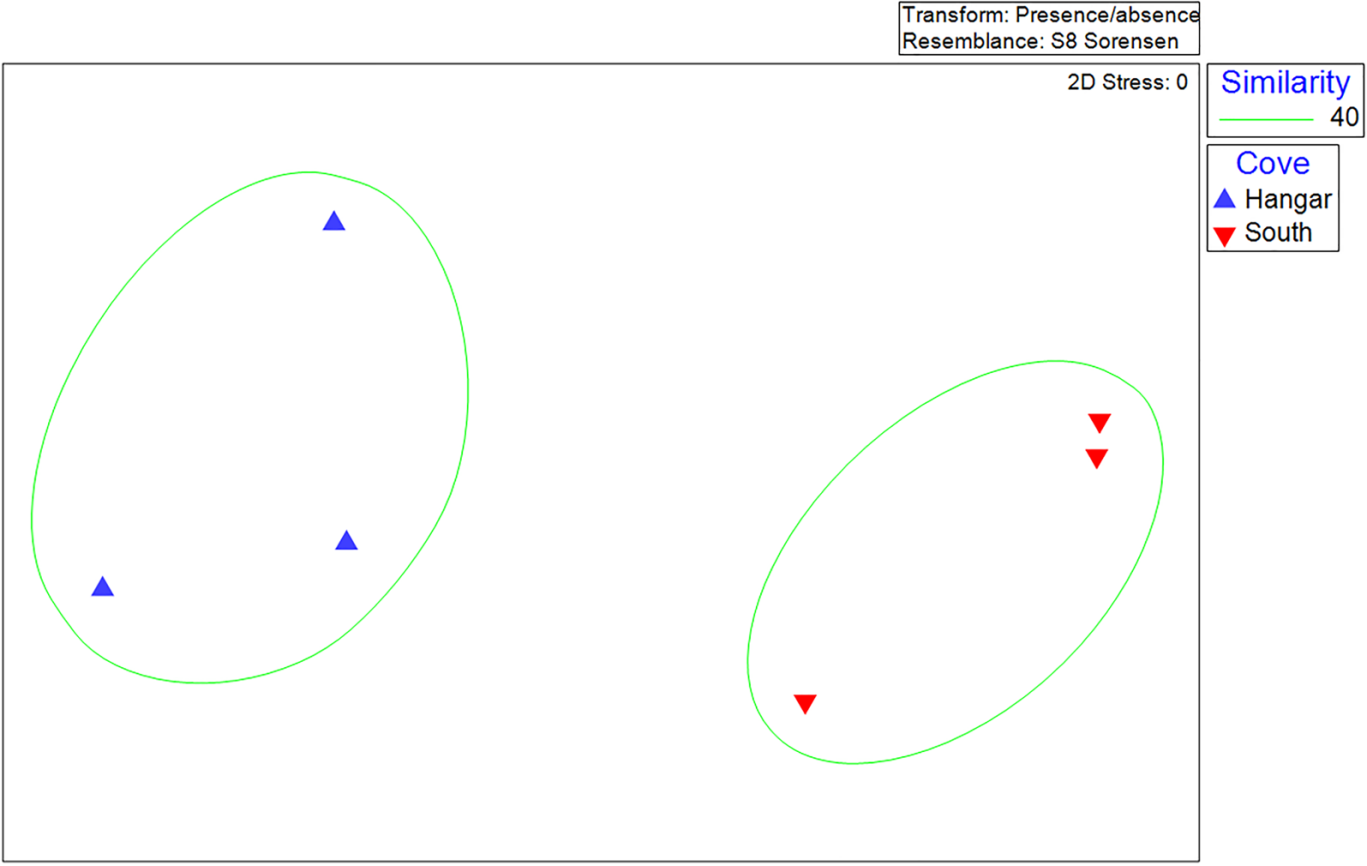
Non-metric multidimensional scaling (nMDS) plots based on the Sorensen similarity matrix for the presence/absence of all metazoan ASVs (amplicon sequence variants). Three replicates from South Cove site C and three from Hangar Cove site F. The low stress value of 0 indicates this plot is a good two dimensional representation of the community in multi-dimensional space. Clustering is shown at a similarity level of 40%

The metazoan ASV richness and diversity were higher than that recorded by morphotaxonomy (Tables 1 and 2), with both recording a higher richness and diversity in South Cove than Hangar Cove (Tables 1 and 2). Similar to the patterns for morphotaxonomy, the two coves were clearly separated from each other by metabarcoding (Fig. 3), with an average of 73% dissimilarity between the coves (ANOSIM). The main taxa responsible for these differences were nematodes (7 ASVs) and then rotifers (3 ASVs).

**Table 2:** Total number of metazoan ASVs (*A*) from 18S RNA ID95% metabarcoding (total of 189), Margalef’s index of Species richness (*d*), Shannon diversity index (*H*′) and associated standard deviations for three replicates from South Cove site C and three from Hangar Cove site F

Site/Cove	*A*	*d*	*H*′(*loge*)
South Cove	146	17.16 ± 4.72	4.28 ± 0.39
Hangar Cove	71	9.53 ± 3.31	3.48 ± 10.45

### Comparison of morphotaxonomy and metagenomics

Due to the limited molecular coverage of Antarctic species it was not possible to compare the taxonomy derived from morphotaxonomy and metabarcoding at the family level. However, nearly twice as many orders (62) were identified by metabarcoding than by morphotaxonomy (37). Of these taxa, only 11 were common to both techniques. Of these 11 orders, 4 were molluscs (Arcoida, Neogastropoda, Nuculidae and Valvatida), 3 were polychaetes (Eunicida, Spionida and Terebellida), 2 were crustaceans (Amphipoda and Isopoda) and 2 were echinoderms (Dendrochirotida and Ophiurida).

### Sediment properties

The sediment properties at each site were characterised by poorly sorted (South Cove site C), or very poorly sorted sediment (all other sites) (Table 3; following the classification of [24]). This variation in grain size was greatest in Hangar Cove with sorting values of 2.87–3.17 ɸ compared to South Cove 1.03–2.65 ɸ. South Cove site C sediment was predominantly composed of sand. The sediment in Hangar Cove contained a much higher proportion of mud (13.3–19.9%) compared to South Cove (1.1–1.9%; *F*_(1,5)_ = 55.1, *P* < 0.01). There were no significant difference in the proportion of gravel (ANOVA; *F*_(1,5)_ = 0.1, *P* = 0.81) or sand (*F*_(1,5)_ = 1.7, *P* = 0.26) between the coves.

**Table 3:** Summary of site depth, sample numbers taken and sediment properties of each sampling site shown in Fig. 1

Cove site	Mean water depth (m)	Mean sediment thickness (cm)	No. of cores (n)	Mean composition (%)			Mean particle size (µm)	Sorting (ɸ)	Skewness (ɸ)	Substratum group
				Gravel	Sand	Mud				
South Cove A	18.6	4.1	26	20.3	77.8	1.9	540	2.42	− 0.65	Gravelly Sand
South Cove B	14.6	3.9	27	39.6	57.8	2.6	705	2.65	− 0.38	Sandy Gravel
South Cove C	21.3	6.1	15	4.2	94.7	1.1	255	1.03	− 0.24	Slightly Gravelly Sand
Hangar Cove D	15.6	5.2	28	17.1	63.0	19.9	223	3.09	− 0.36	Gravelly Muddy Sand
Hangar Cove E	19.5	4.6	18	28.3	58.4	13.3	478	3.17	− 0.32	Gravelly Muddy Sand
Hangar Cove F	16.7	7.3	10	20.9	66.4	12.6	391	2.87	− 0.23	Gravelly Muddy Sand

### Relationship between benthic assemblages and sediment properties

The single sediment characteristic which best grouped the benthic assemblage of sites, from morphotaxonomy, was the proportion of organic matter in the sediment (BIOENV; *R *= 0.664, *P* < 0.001). This correlation was stronger than any two or three combinations of other variables tested (Table 4).

**Table 4:** Combinations of sediment grain size and sediment thickness yielding the best matches of biotic and abiotic similarity matrices as measured by a Spearmans rank correlation (*ρ*_s_)

No. variables	*ρ*_*s*_	Sediment properties	*P*
1	0.664	AFDM %	0.001
2	0.662	AFDM %, Mud %	0.001
1	0.661	Mud %	0.001
3	0.648	Sand %, Mud%, AFDM %	0.001
2	0.63	Mud %, Sand %	0.001
3	0.622	Mud %, Sediment depth, AFDM %	0.001

Relationships between sediment properties and the benthic assemblage calculated using PRIMER BEST Biota-environment (BIOENV) procedure

*AFDM* ash free dry mass

## Discussion

**Fig. 4:**
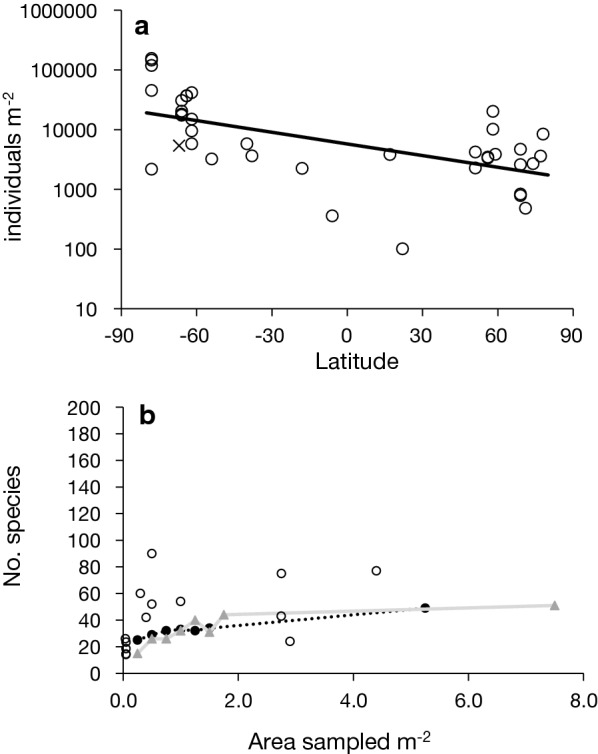
Meta-analysis of **a** abundance (density, m^−3^) across latitudes and **b** species richness of sediment communities. Abundance from samples using 1 mm mesh were doubled to compare with those from 0.5 mm mesh, following White and Marine benthos [25]. Average abundance from the current study is marked with a cross. Regression line log10(abundance) = 3.7609–0.0065 latitude. Species richness data from Hangar Cove (dotted line) and South Cove (solid line) were randomly selected and summed to create finders curves for each Cove. Species richness data from the literature are plotted against the sampled area. To allow comparison with the current study, studies that sampled 8 or more m^−2^ were not included. References for the abundance and species richness meta-analysis are listed in Additional file 1

This is the first study to comprehensively analyse the composition of Antarctic soft sediment metazoan communities across all size classes, from < 1 mm up to 10 cm, in two geographically distinct coves. These data strongly confirm the few previous analyses that showed there were a wide range of densities of mega and macro-fauna recorded within Antarctic soft sediments, 464 to 16,084 individuals m^−2^, which match the wide range of densities recorded for other Antarctic and global locations (Fig. 4a; Additional file 2: Table S3). There was a significant linear reduction in the density of individuals with increasing latitude (F_(1,37)_ = 18.4, P < 0.01) from Antarctic to Arctic sediments (Fig. 4a) with no significant difference between hemispheres (F_(1,37)_ = 1.7, P = 0.21). This may be largely driven by the very high numbers of individuals recorded in McMurdo Sound sponge spicule beds [26].

Biodiversity is high in this region of the Southern Ocean [12, 17, 18]. While literature values for soft sediment macro- and mega- fauna species richness vary considerably from study to study, the diversity of approximately 50 species/m^2^ found in the current study is within the range reported in the literature for other latitudes (Fig. 4b). Values have been reported from as low as 8 m^−2^ in Norway [27] to 650 m^−2^ in East Greenland [28]. Other values from the Antarctic also vary across a similar range, from 8 m^−2^ in Admiralty Bay [29] to 469 m^−2^ in O’Brien Bay near Casey station [30]. The addition of metabarcoding data shows that the inclusion of meiofauna greatly increased the richness measured. However, the morphotaxonomy data surprisingly reveal that the particular ecological drivers found in the Antarctic, namely seasonality and iceberg scour, had little effect on community composition, implying that similar to other regions of the world, organic content and sediment grain size are the major drivers of sediment biodiversity.

Seasonality is known to have a major effect on Antarctic shallow water benthic organisms [31]. It drives key physiological processes, particularly in primary consumers, with the quality and availability of food impacting not only on performance, but also reproductive capacity [32, 33]. However, the majority of studies to date have investigated the effect of seasonality on individual species and little is known about how this very dramatic “feast or famine” regime affects community composition [34]. Interestingly this study detected no seasonal differences in soft sediment community structure around Rothera Point. Thus, indicating that the seasonal variation in food supply from the phytoplankton does not lead to detectable changes in local community structure between summer and winter. While the difficulty of winter sampling resulted in a bias towards summer samples, the lack of seasonal differences in community composition is likely due to the very low temperatures reducing overwintering costs in polar regions, and the extended longevity of high latitude marine species [33, 35]. The lack of seasonal variation in many sediment processes, such as respiration, and recruitment may be due to the accumulation of a persistent food banks in the sediment [36, 37]. It is possible that more spatially extensive metabarcoding data could reveal seasonal and or interannual differences, particularly in the smaller component of the community.

Far more marked, was the difference between the two adjacent coves, emphasizing the patchiness of Antarctic biodiversity, even at relatively small scales [21, 22, 38]. Both techniques recorded a lower species diversity and richness in Hangar Cove than South Cove. Morphotaxonomy also recorded a lower evenness as well as higher species dominance compared with South Cove. This trade-off between diversity and dominance matches the expectations of terrestrial ecological theory [39], which is mirrored in other sediment studies across latitudes [40–42]. Both morphotaxonomy and metabarcoding recorded similar high levels of difference in species between the coves, with 54% dissimilarity in taxa identified through morphotaxonomy and approximately 80% of orders recorded from ASVs were unique to one or other cove. This matches with Sicinski et al. [43] whose comparison of two different glacial coves within Admiralty Bay, King George Island, found an 80–90% dissimilarity in species composition between their coves. Neither Hangar nor South Cove are glacial, but are similarly bounded on one side by ice cliffs and on the other by Rothera Point. The greater duration of winter sea ice cover in Hangar, rather than South, Cove may contribute to some of the differences between coves. The dynamics of the spring phytoplankton bloom are, however, set at a larger scale, that of Marguerite Bay [14] with both coves being bathed in the same water mass. Any future changes in primary productivity, due to changes in stratification or light intensity are therefore likely to affect both coves.

Morphotaxonomy and metabarcoding produced quite different taxonomic results as they sampled either macro and mega, or micro-fauna respectively. However, both techniques demonstrated similar levels of community differences between the coves, the main differences were due to taxa from different size groups. Analysis of samples collected using the suction sampler suggested that bivalves, cnidarians and polychaetes accounted for the main differences, whilst metabarcoding, using the 18S rRNA gene, identified nematodes, crustaceans, platyhelminthes and bivalves as the main taxonomic differences.

The difference between methods is perhaps not surprising given the fact that different taxa are characteristic of the different size classes and thus the two techniques were complementary by showing several dominant taxa in the benthos. The latter finding is in agreement with previous studies on Antarctic meiobenthos using both morphotaxonomy and metabarcoding approaches [44–46]. Additionally, the 18S rRNA is also known to have a slower molecular evolution rate making it a more valuable marker for distinguishing between samples at higher taxonomic levels [47]. On the other hand the results from COI mitochondrial gene evidenced that arthropods and annelids also dominated the benthos, which coincides more closely with the morphological approach. This happens since the COI is widely used as a DNA barcode for species identification [48] and because of this it has a high molecular resolution at the species level [47], although COI is not the most accurate marker for discriminating at higher taxonomic rankings [49].

These dominance differences and also the differences between coves revealed by the two techniques may be due to several reasons. The main one is obviously size selection, as suction sampling only collected organisms larger than 1 mm in size, which almost certainly accounts for the dominance of bivalves, particularly *Altenaeum charcoti* and *Aequiyoldia eightsi.* The vast majority of nematodes and crustaceans are too small to be sampled by the suction sampler but will be picked up by metabarcoding. However, the other major factor is the potential destruction of fragile or soft bodied species during the collection for morphotaxonomy, particularly nematodes and Platyhelminthes. Metabarcoding would identify these taxa as present, even if individuals were lethally damaged and unrecognizable as whole animals. eDNA samples include traces of animals, such as mucus trails, faeces, decaying tissue in the sediment and early life history stages that can be detected at the molecular level, even if not physically observed in the sample. In spite of these differences, some taxa were common to both techniques and the results of both techniques validated the overall higher taxonomic diversity in South Cove than Hangar Cove and a similar level of difference between the coves. The combined data of the current study, coupled with the high numbers of unique taxa, indicate that regional biodiversity (β-diversity) is high. They also emphasize the utility of combining different techniques to gain a more complete picture of overall biodiversity in soft sediments, particularly for the soft bodied microfauna [cf. 44, 50]. Overall both approaches proved to be quite complementary where the 18S rRNA gene allowed to assess general ecological patterns at higher taxonomic rankings like family or genus level but it also served as an auxiliary tool in this study, where information on morphological characteristics was available and also an additional marker such as the COI gene provided species-level resolution. Further to this, the eDNA approach also revealed high numbers of unassigned taxa (NAs) mainly reflecting the poor existing reference databases for Antarctic species [51]. Additionally, the 18S marker is well known to have a poor species-level resolution mainly due to being highly conservative [52]. Consequently some assignments will likely be assigned to non-polar species, which will significantly decrease the accuracy and number of the taxonomic assignments.

What was more surprising than a lack of seasonal effect on biodiversity was the absence of a correlation between biodiversity levels and recent iceberg impact. On a global scale diversity is generally correlated with levels of disturbance [53]. Many polar marine studies have attributed patchiness in benthic environments to physical disturbance from iceberg scour, and highlighted this as a dominant factor in structuring communities [cf. 54]. Iceberg scouring is a major forcing factor of Antarctic hard substratum benthic assemblage composition, the effects of which vary between taxa [e.g. 23]. Previous studies on hard substrata found that within 1 month of iceberg impact abundances of bivalves, polychaetes and ostracods were reduced, when compared to controls, but not gastropods or amphipods [15, 55].

While only five of the 51 samples in the current study had a known disturbance history, one of these was measured three times within the first 39 days, which is within a similar time-frame over which reductions in biodiversity were measured in the hard substrata studies. Despite the small sample size the lack of any disturbance signal on our soft sediment sites was unexpected. This implies that icebergs may have less effect on soft sediment communities compared with those found on hard substrata. For example, it is likely that the sediment provides a cushion or softening effect against direct impacts and organism damage [56]. In addition sediments are easily moved by water currents, but more particularly during storms, as these are particularly important for the recolonisation by some taxa [57]. Organisms may also be redistributed via the wash from adjacent grounded icebergs, which means that meiofauna in sediments can redistribute very quickly, increasing the speed with which sediment patches can recover [45]. Nematodes, which are one of the dominant taxa identified by metabarcoding, are little affected by disturbance [45]. This is possibly due to easy redistribution from adjacent sites, which may contribute to their prevalence in soft sediments [45].

This is particularly pertinent to Hangar Cove, where one site was sampled within 7 days of initial impact and this cove comprises a higher percentage of fine mud particles. Furthermore, some of the more dominant taxa identified in sediments, such as polychaetes and crustaceans, are highly mobile and thus rapidly recolonise impacted sites [57]. Although there was no evidence of recent iceberg disturbance acting on the outlier sample from Hangar Cove, the high numbers of mobile scavengers (amphipods and urchins), and low numbers of the dominant infauna, means that an unidentified iceberg impact cannot be ruled out. Alternately, these species may have been attracted to a nearby food source, such as a carcass. Little is known about the life history traits of the smaller sediment-living species, but there is evidence that harsher environments select for more resilient R-selected species [58]. However, generation times are slowed in Antarctic species and the fauna are more generally K-selected [58]. The lack of variation suggests that they persist as a consequence of low temperatures, rather than rapid reproductive replacement, and hence this is a major difference to lower latitude, warmer sites.

Hence, although this study surveyed Antarctic sediments that experience the strong structuring forces of seasonality and iceberg impact, community structure is, like sediment communities in other regions of the world, highly correlated with organic content. The latter is generally inversely correlated with particle size [10], with species diversity tending to be highest at intermediate levels of sediment organic enrichment [59, 60]. Therefore the dominance of diatom blooms in the majority of coastal seas [61] and the importance of sediment structure may constrain sediment communities across latitudes. This study also adds to the evidence that the overall abundance and diversity of Antarctic soft sediment communities is high and broadly similar to that of other latitudes [18, 28, 62].

Sea temperature, sea ice duration, iceberg disturbance, melt water run-off and plankton blooms are all rapidly changing in the polar-regions [8, 14, 23, 63]. It is, therefore, important to understand the key factors that will determine the structure of soft sediment communities into the future [64]. The results of this study suggest that increased sedimentation from melting glaciers may be the biggest impact of current climate change on soft sediment communities [8]. Previous studies of Antarctic shallow water sedimentary meiofauna have concentrated on areas of rapid glacier retreat with the aim of understanding re-colonisation processes [20–22]. These suggest that the microfauna may respond more slowly than the more mobile macrofauna [19]. Thus studies such as the one described here, where sediment communities are described across all size classes of organism (from macro- to microfauna) are vital to understanding not only community structure, but also ecosystem functioning and trophic interactions. One particular issue that needs to be addressed in the future is the correlation between species composition, energy transfer and nutrient cycling, as this has far-reaching implications for the wider benthic ecosystem and bentho-pelagic coupling. The meio- and microfauna in sediments can only be efficiently characterised using molecular methods. Metabarcoding provides a catalogue of species, but not their functions, whereas often with the megafauna there is at least rudimentary knowledge of the feeding modes (primary consumer, secondary consumer, carnivore, scavenger, detritivore, etc.) and therefore a basic classification within the food web. In the future these molecular types of studies need to be more closely linked to genomic shotgun sequencing and transcriptomic approaches to evaluate active biochemical processes in the sediment and identify the species responsible for them. This will significantly aid in our understanding of the functional roles of the various community components present in these poorly studied environments, and their potential responses to future climate change.

## Conclusions

Studies combining multiple techniques, including morphotaxonomy and metabarcoding are required to gain a complete picture of the drivers controlling community structure in one of the most abundant habitats on earth, soft marine sediments. This study measured high biodiversity in soft sediment communities, and in contrast to findings from rocky substrata, there was no evidence of an effect of typical Antarctic stressors of iceberg scour and intense seasonality. As at other latitudes, organic content of the sediment was most strongly correlated with community structure, suggesting that increased sedimentation from run-off from melting glaciers may be the main climate change effect on these communities.

## Methods

### Permit and ethical considerations

As required by the Antarctic Treaty, all research was conducted after a preliminary environmental assessment and under a permit issued by the UK Foreign and Commonwealth Office. All importation of specimens into the UK was under relevant UK government department permits (DEFRA—Department of the Environment, Food and Rural Affairs). This study did not involve animal experiments and so no ethical approval was required.

### Field sites

The sampling was conducted in two coves adjacent to the British Antarctic Survey research station at Rothera Point, Adelaide Island, West Antarctic Peninsula (67°34.5′S, 68°07.0′W).

**Fig. 5:**
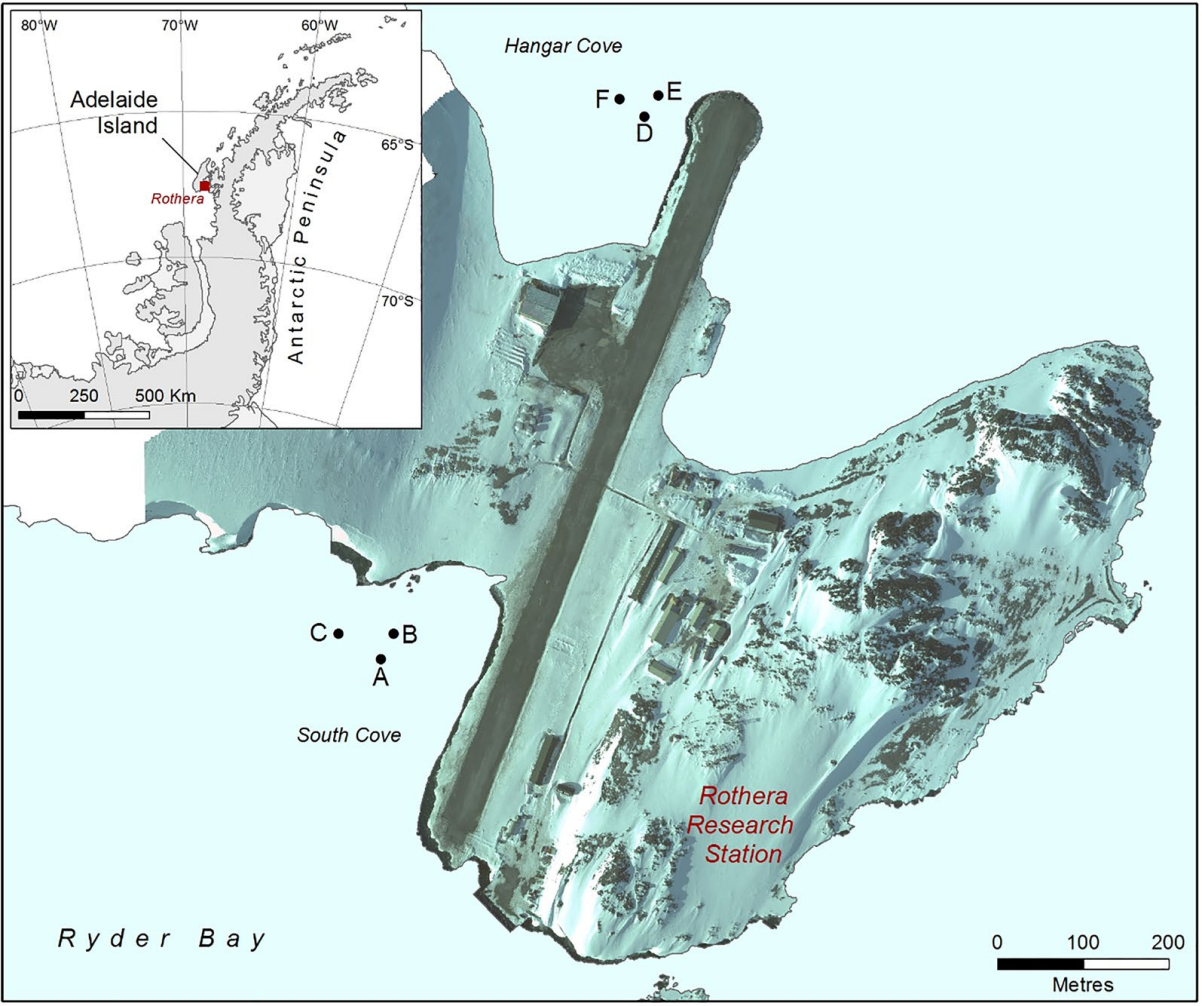
Location of study sites: South Cove (sites A, B and C) and Hangar Cove (sites D, E and F) around Rothera Point, Adelaide Island, Antarctica (67°34.5′S, 68°07.0′W). Inset (top) shows position of Adelaide Island on the Antarctic Peninsula. Map produced by Mapping and Geographical Information Centre, British Antarctic Survey

The substratum in South Cove is a mixture of bedrock and cobbles with patches of soft sediment. Conversely, Hangar Cove primarily has a base of compacted cobbles covered with a thin layer of fine sediment (2–3 cm) interspersed with patches of, deeper, fine sediment. In both coves the thickness of the soft sediment ranges from 0 to ~ 20 cm with a gently sloping topography. During summer icebergs impact the benthic communities in these coves and in the winter the sea surface is covered by fast ice for 3 to 8 months [15]. For this study, searches were conducted in the 15–21 m depth range for large patches of soft sediment. Six sites were selected within the two coves; South Cove (Fig. 5, sites A, B and C) and Hangar Cove (Fig. 5, sites D, E and F).

In Spring 2014 concrete markers were laid at each site 1 m apart in grids, each covering an area of 9 m^2^ (akin to the design developed by Brown et al. [15] and described further by Barnes et al. [16], providing both a grid reference for sample extraction and a method of monitoring iceberg impacts). The effect of any recorded disturbance of the grid markers could therefore be correlated with sediment community composition. To further test for the effect of iceberg scour on soft substratum community dynamics, Site F was situated on the site of a recent scour.

All sampling was conducted during a 2.5 year period from the start of the austral summer 2013 to the end of summer 2015 (Additional file 2: Table S2); winter is hereafter defined as June to September and summer from October to May inclusive. Due to the challenges of winter diving, most samples were collected in summer. Three Hangar and three South Cove samples were collected in summer 2012/2013, five Hangar and 12 South Cove samples were collected in summer 2013/2014. Four Hangar and six South Cove samples were collected in winter 2014 and nine samples from each of Hangar and South Cove were collected in summer 2014/2015. Samples for metabarcoding were only collected in summer from South Cove site C and Hangar Cove site F.

### Faunal sampling

#### Suction sampling for mega and macro-fauna

To investigate benthic assemblages, SCUBA (self-contained underwater breathing apparatus) divers used a 10 cm diameter suction sampler fitted with a 1 mm^2^ mesh sampling bag to extract the benthos from 0.25 m^2^ quadrats. For each site (A to F) quadrats within the 3 × 3 m grid were chosen using a random number generator but if the selected area of seabed was adjacent to a quadrat that had been sampled within the previous 6 months another quadrat was randomly selected. This was a precaution taken to ensure data were not affected by any small disturbance caused by divers in the adjacent quadrat during a previous sampling. Organisms retained within the 1 mm^2^ mesh suction sampler bag were taken back to the laboratory counted and sorted into morphotypes, while alive. Individuals were either identified during sorting were or identified later from preserved specimens. Individuals greater than 1 mm but less than 5 mm were classified as macrofauna and anything larger (up to 10 cm) as megafauna. 99% of the Mollusca were identified to genus level or higher. Individuals belonging to all other Phyla were identified as far as possible, at least to a minimum of family level, by experts using taxonomic keys. Fragments of drifting algae and any exposed pebbles that may have been colonized by encrusting fauna, e.g. bryozoans and spirorbid polychaete worms also collected by the suction sampler were not included in the subsequent mega and macro-faunal community analysis. Post extraction, the removed sediment thickness was measured at five random points using a ruler and the average sediment thickness was calculated.

#### Metabarcoding for microfauna

Benthic sediment samples were collected in triplicate from South Cove site C and Hangar Cove site F, in the summer of 2014 only, using a 100 cm^3^ corer and kept at − 80 °C. For all sediment cores the upper 20 cm^3^ of the core were screened using a 45 μm sieve, and 8–10 g of the homogenized sediment was used for direct eDNA extraction, using the PowerMax^®^ Soil DNA Isolation Kit (MO-BIO) following the manufacturer’s instructions. DNA extracts were visualized by agarose gel electrophoresis, quantified using the NanoDrop2000 spectrophometer and diluted to 10 ng/µL. The universal primers ‘TAReuk454FWD1’ (5′-CCAGCASCYGCGGTAATTCC-3′) and ‘TAReukREV3’ (5′-ACTTTCGTTCTTGATYRA-3′) were used to amplify ca. 400 bp of the 18S rRNA V4 region [65]. The mitochondrial COI 313 bp gene region was amplified using the primers forward “mlCOIintF” (5′ GGWACWGGWTGAACWGTWTAYCCYCC-3′) and reverse “jgHCO2198” (5′-TAAACTTCAGGGTGACCAAARAAYCA-3′) [66]. All forward and reverse primer combinations were designed to include the Illumina MiSeq 8 nt index-tags (i5/i7) and Adaptors (P5/P7) to differentiate all samples and triplicates. PCR conditions were performed similarly to the Fonseca and Lallias [44] method. Briefly, PCR amplification of the specified nSSU region was performed with a 2-step PCR approach using 1 µl of genomic DNA template (1:500 dilutions; 10 ng/μl) in 3 × 40 µl independent reactions with Pfu DNA polymerase (Promega). The first PCR involved a 5 min denaturation at 95 °C, followed by 15 and 20 cycles (18S and COI) with 30 s 98 °C, 30 s 50 °C, 30 s 72 °C and final extension of 10 min at 72 °C. At this stage, amplicons from the first PCR were purified using the HT ExoSAP-IT (Affymetrix) following manufactures’ instructions. To add the Illumina index tag adaptors a second PCR using 7 µl of purified PCR1 products was performed using the same conditions as before but with 15 cycles and annealing temperature of 55 °C. Negative controls (ultrapure water only) were included for all amplification reactions. PCR products were visualized and purified (QIAquick Gel Extraction Kit, Qiagen) in an agarose gel and quantified using the Agilent Bioanalyser. All PCR products were diluted to the same concentration of 3 ng/μl, pooled together to create one amplicon-library and pair-end sequenced on a single run of the Illumina Miseq platform using the v2 Illumina chemistry (2 × 250 bp). Illumina raw sequences from both markers (18S and COI) were analysed using the dada2 plugin within QIIME2 [67]. This plugin does not rely on OTU clustering, but rather utilizes modern sequencing quality by producing fine-scale resolution through amplicon sequence variants (ASVs), resolving differences of as little as a single nucleotide [67]. Its workflow consists of: filtering, de-replication, sample inference, reference-free chimera detection, and paired-end reads merging [67]. ASVs with less than four sequences were discarded and taxonomies assigned by applying the QIIME2 consensus blast ‘q2-feature-classifier’ with the ‘classify-consensus-blast command’ [68] using 0.95 and 0.97 classification thresholds for the 18S rRNA and COI, respectively. The 18S rRNA data was used to infer all ecological patterns at higher taxonomic ranks whereas the COI data was only used to compare the number of species identified by the morphology and eDNA metabarcoding approaches.

#### Sediment sampling

At the same time as suction samples were collected, additional cores were taken for sediment particle size analysis. All sediment cores were collected in triplicate from undisturbed sediment at each site using a cylindrical hand-held plastic corer of 8 cm diameter × 20 cm length. Extracted sediment from all three cores was mixed, rinsed with fresh water and subjected to sequential decantation of percolated material to reduce the salt content. This was then dried and weighed. To measure organic content a random subsample of dry sediment (approx 150 g) was taken and weighed. Organic content was quantified through the loss on ignition method (after [69]), where samples were dried to constant weight at 68 °C and then ignited at 475 °C for 24 h to obtain the proportion of Ash Free Dry Mass AFDM [(dry weight) − (ash weight)]/(dry weight). The remaining sediment was weighed and passed through a stack of sieves in a vibrating sieve shaker (15 min, 50 kpa). Each distinct fraction was weighed and the percentages of gravel (> 2 mm), sand (2 mm to < 63 μm) and mud (< 63 μm) were recorded for each site. Mean size, sorting and skewness were calculated using GRADISTAT [70] following [71].

#### Statistical analysis

Assemblage comparisons were calculated using family level identifications or ASV’s from 18S rRNA and multivariate analyses performed using PRIMER v.7 software [24, 71]. Due to the presence/absence nature of metabarcoding data, evenness and dominance could not be calculated.

For morphotaxonomy multivariate analyses abundance data were fourth root transformed and non-metric multi-dimensional scaling (nMDS) applied to a Bray–Curtis similarity matrix, to produce ordination plots, representing the similarity in benthic assemblage amongst sites and coves [24]. Fourth root was determined as the most appropriate transformation through comparison of shade plots, which visualize the influence of extreme values under different transformations. For metabarcoding, Sørensen’s similarity coefficient among samples was computed based on a presence/absence similarity matrix and was used to create cluster dendrograms and nMDS with 500 random starts.

A similarity profile (‘SIMPROF’) permutation test, was performed on group-average cluster analysis to test whether mega/macro-benthic and meiobenthic samples differed from each other. The benthic assemblage groupings identified in the nMDS ordinations were further explored using the similarities percentages routine (SIMPER) to identify the taxa contributing most to dissimilarities between coves and to the similarities within each cove.

For morphotaxonomy only, the ANOSIM (analysis of similarities) *R* statistic was used to test for statistical differences both spatially between coves and sites and temporally, between season (winter/summer) and year. A one-way ANOSIM was used to test if differences between coves could be aligned to functional traits using a binary family-by-trait matrix based on three traits; Mobility (Errant or Sedentary), Size (Megafauna or Macrofauna) and Lifestyle (Epifauna or Infauna).

To investigate taxon surrogacy, i.e. test whether there was any substantial loss of information from using families instead of genera, the overall trend at the family level assemblage was compared to the genera assemblage in one of the most common Phyla identified through morphotaxonomy, the Mollusca [27].

As residuals were normally distributed (Anderson–Darling test) and the variances of data were homogeneous (Levene’s test) differences in abundance, community richness, evenness, diversity and dominance between sites and coves were tested with ANOVA. Particle size and organic content data were normalized through arcsine square root transformation, followed by ANOVA tests for community differences between sites and coves.

Correlations between benthic assemblages and the sediment characteristics (% gravel, % sand, % mud, sediment thickness and % ash free dry mass (AFDM) and water depth) were analysed using PRIMER BEST Biota-environment (BIOENV) to determine which factors best explained the distribution of the faunal community. All data were normalised before analysis.

## Additional files

**Additional file 1: Table S1.** Average number of individuals 0.25 m-2 collected at each site (South Cove, A–C; Hangar Cove, D–F).

**Additional file 2: Table S2.** The date, position and water depth of samples taken in summer and winter. The square of the IBIS (iceberg disturbance) grid associated with the sample, is also reported.* indicates that the position was calculated from position from previous samples. **Figure S1.** Non-metric multidimensional scaling (nMDS) plots based on the Bray–Curtis similarity matrix for abundance of Mollusca, identified to the genus level. Benthic assemblages at South Cove, circles (Sites A, B and C) and Hangar Cove, triangles (Sites D, E and F). The low stress value of 0.15 indicates this plot is a good two-dimensional representation of the community in multi-dimensional space. Clustering shown at a similarity level of 50%. Description of outlier, Hangar Cove, site E. **Table S3.** Meta-analysis of the abundance (density, m-3) of sediment communities across latitudes. Abundance from samples using 1 mm mesh were doubled to compare with those from 0.5 mm mesh (following Arrigo and Dijken [63]). Reference lists for abundance and species richness mata-analyses.

**Additional file 3: Table S4.** Summary results of the metabarcoding in silico analysis for both markers tested (COI and 18S rRNA) using QIIME2. Total number of raw reads and reads after each step in the pipelines are shown as well as total number of Amplicon Sequence Variants (ASVs) after running Qiime2. ASVs assigned to both Eukaryotes and Metazoa are shown for the different BLAST sequence identity matches (90%, 95% and 97%) and also Not assigned ASVs for both markers using 95% BLAST match. Shaded grey; data used for ecological patterns and community profiles at higher taxonomic ranking. (*) ASVs used to compare species identification detected by eDNA and morphology. Amplicon Sequence Variants; ASVs.

**Additional file 4: Table S5.** eDNA species list retrieved using the COI marker using a 97% sequence BLAST match against GenBank database. First column shows ASVs identification tag, followed by number of reads allocated to each ASVs per sample site (HC1, HC2, HC3) and the last seven columns show the taxonomy ranking to each ASVs from Kingdom to species, respectively. Hangar Cove replicates 1–3 (HC1, HC2, HC3) and South Cove replicates 1–3 (SC1-3).

## Abbreviations

ASV: amplicon sequence variants
DEFRA: Department of the Environment, Food and Rural Affairs
SCUBA: self-contained underwater breathing apparatus
nMDS: non-metric multi-dimensional scaling
SIMPER: similarities percentage routine
ANOSIM: analysis of similarities
AFDM: ash free dry mass
BIOENV: primer best biota-environment
NA: unassigned taxa
