# Supplementary material for: Spatial and temporal dynamics of Antarctic shallow soft-bottom benthic communities: ecological drivers under climate change
Source: BMC Ecol. 2019 Jul 1;19:27. doi: 10.1186/s12898-019-0244-x (PMC6604130; doi:10.1186/s12898-019-0244-x)
Supplement: Supplementary file 1 — Additional file 1: Table S1. Average number of individuals 0.25 m-2 collected at each site (South Cove, A–C; Hangar Cove, D–F). [file 12898_2019_244_MOESM1_ESM.docx]

| Average number of individuals 0.25m^-2^ for each site. | | | | Sites A-C South Cove. | |  |
| --- | --- | --- | --- | --- | --- | --- |
| **Phylum** | **Class** | **Order (where there is no order f:family, sf:superfamily)** | **Family** | **A** | **B** | **C** |
| Annelida | Polychaeta | Capitellidae (f) | Capitellidae | 0.1 ± 0.3 | 0 | 0.3 ± 0.5 |
| Annelida | Polychaeta | Eunicida | Dorvilleidae | 0.2 ± 0.4 | 0 | 0 |
| Annelida | Polychaeta | Maldanidae (f) | Maldanidae | 0.6 ± 1.6 | 0 | 7.4 ± 12.6 |
| Annelida | Polychaeta | Opheliida | Opheliidae | 0.9 ± 1.9 | 0.1 ± 0.3 | 0.4 ± 0.7 |
| Annelida | Polychaeta | Orbiniida | Orbiniidae | 80.8 ± 65.1 | 100.4 ± 47.3 | 67.2 ± 44.6 |
| Annelida | Polychaeta | Phyllodocida | Nephtyidae | 12.2 ± 6.6 | 16.9 ± 7.7 | 10.7 ± 5.9 |
| Annelida | Polychaeta | Phyllodocida | Nereididae | 0 | 0 | 0.1 ± 0.3 |
| Annelida | Polychaeta | Phyllodocida | Phyllodocidae | 0 | 0 | 0 |
| Annelida | Polychaeta | Phyllodocida | Polynoidae | 2.9 ± 3.1 | 2.1 ± 2.1 | 0.8 ± 1.4 |
| Annelida | Polychaeta | Phyllodocida | Syllidae | 0 | 0 | 0.1 ± 0.3 |
| Annelida | Polychaeta | Polygordiidae (f) | Polygordiidae | 0 | 0 | 0 |
| Annelida | Polychaeta | Spionida | Apistobranchidae | 0 | 0 | 0 |
| Annelida | Polychaeta | Spionida | Spionidae | 0 | 0 | 0.1 ± 0.3 |
| Annelida | Polychaeta | Terebellida | Ampharetidae | 0.3 ± 0.5 | 0.1 ± 0.3 | 0.2 ± 0.6 |
| Annelida | Polychaeta | Terebellida | Cirratulidae | 8.5 ± 15.6 | 6.3 ± 7.7 | 63.5 ± 97.1 |
| Annelida | Polychaeta | Terebellida | Terebellidae | 0.3 ± 0.5 | 0 | 2.8 ± 4.8 |
| Arthropoda | Malacostraca | Amphipoda | Amphilochidae | 0 | 0.1 ± 0.3 | 0 |
| Arthropoda | Malacostraca | Amphipoda | Calliopiidae | 1.2 ± 2.3 | 2.1 ± 1.9 | 0.3 ± 0.5 |
| Arthropoda | Malacostraca | Amphipoda | Exoedicerotidae | 0.8 ± 0.8 | 3.4 ± 2.7 | 1.5 ± 1.3 |
| Arthropoda | Malacostraca | Amphipoda | Lysianassidae | 3.1 ± 4 | 0.1 ± 0.3 | 0.9 ± 2.1 |
| Arthropoda | Malacostraca | Amphipoda | Oedicerotidae | 21.9 ± 12 | 21.8 ± 9.3 | 27.8 ± 16.8 |
| Arthropoda | Malacostraca | Amphipoda | Phoxocephalidae | 0.6 ± 1 | 0.7 ± 1 | 1.1 ± 1.3 |
| Arthropoda | Malacostraca | Amphipoda | Pontogeneiidae | 8.4 ± 12.7 | 15.1 ± 27.3 | 1.6 ± 3.8 |
| Arthropoda | Malacostraca | Amphipoda | Stenothoidae | 3 ± 8.2 | 0.7 ± 0.9 | 0.4 ± 0.5 |
| Arthropoda | Malacostraca | Cumacea | Bodotriidae | 0.8 ± 1 | 0.8 ± 1.1 | 1.5 ± 2.1 |
| Arthropoda | Malacostraca | Cumacea | Diastylidae | 0 | 0 | 0.1 ± 0.3 |
| Arthropoda | Malacostraca | Cumacea | Leuconidae | 0 | 0 | 0 |
| Arthropoda | Malacostraca | Cumacea | Nannastacidae | 3.4 ± 4.4 | 0.7 ± 0.5 | 2.8 ± 4 |
| Arthropoda | Malacostraca | Euphausiacea | Euphausiidae | 0 | 0 | 0 |
| Arthropoda | Malacostraca | Isopoda | Gnathiidae | 0 | 0 | 0.3 ± 0.5 |
| Arthropoda | Malacostraca | Isopoda | Janiridae | 0 | 0 | 0 |
| Arthropoda | Malacostraca | Isopoda | Sphaeromatidae | 0.1 ± 0.3 | 0.7 ± 0.7 | 0.2 ± 0.4 |
| Arthropoda | Malacostraca | Mysida | Mysidae | 0.8 ± 1.8 | 0 | 0.4 ± 1.2 |
| Arthropoda | Malacostraca | Tanaidacea | Nototanaidae | 0.4 ± 1 | 0.1 ± 0.3 | 0 |
| Arthropoda | Ostracoda | Myodocopida | Philomedidae | 0.3 ± 0.7 | 0.2 ± 0.4 | 0.4 ± 0.7 |
| Cephalorhyncha | Priapulida | Priapulidae (f) | Priapulidae | 0 | 0 | 0 |
| Chordata | Actinopterygii | Perciformes | Artedidraconidae | 0 | 0 | 0.1 ± 0.3 |
| Chordata | Actinopterygii | Perciformes | Nototheniidae | 0 | 0 | 0 |
| Cnidaria | Anthozoa | Actiniaria | Edwardsiidae | 0 | 0 | 12.5 ± 41.3 |
| Echinodermata | Asteroidea | Forcipulatida | Asteriidae | 0 | 0 | 0.1 ± 0.3 |
| Echinodermata | Asteroidea | Valvatida | Odontasteridae | 1.8 ± 2.7 | 3.3 ± 2.5 | 0.6 ± 0.8 |
| Echinodermata | Echinoidea | Camarodonta | Echinidae | 3.2 ± 6.2 | 8.2 ± 4.6 | 0.8 ± 1.4 |
| Echinodermata | Holothuroidea | Dendrochirotida | Cucumariidae | 0.1 ± 0.3 | 0 | 0.1 ± 0.3 |
| Echinodermata | Holothuroidea | Dendrochirotida | Psolidae | 0 | 0 | 0 |
| Echinodermata | Ophiuroidea | Ophiurida | Ophiuridae | 35.3 ± 21.3 | 27.8 ± 12.1 | 47.5 ± 12.8 |
| Mollusca | Bivalvia | Anomalodesmata | Laternulidae | 7.5 ± 16.3 | 17.8 ± 23.5 | 14.7 ± 14.7 |
| Mollusca | Bivalvia | Arcoida | Philobryidae | 0.1 ± 0.3 | 0 | 0.3 ± 0.5 |
| Mollusca | Bivalvia | Lucinoida | Thyasiridae | 0 | 0 | 0 |
| Mollusca | Bivalvia | Nuculanoida | Yoldiidae | 31.8 ± 41.9 | 189.6 ± 129.9 | 93.5 ± 89.2 |
| Mollusca | Bivalvia | Veneroida | Cyamiidae | 0.6 ± 1.3 | 0.3 ± 0.7 | 0 |
| Mollusca | Bivalvia | Veneroida | Montacutidae | 1.1 ± 1.6 | 0.4 ± 0.7 | 0.3 ± 0.5 |
| Mollusca | Gastropoda | Littorinimorpha | Eatoniellidae | 1.4 ± 2.1 | 1.1 ± 0.8 | 1.8 ± 1.5 |
| Mollusca | Gastropoda | Littorinimorpha | Littorinidae | 4.8 ± 10.1 | 5.3 ± 4.1 | 1.6 ± 2.4 |
| Mollusca | Gastropoda | Littorinimorpha | Rissoidae | 1.6 ± 1.6 | 0.6 ± 0.9 | 6 ± 10.4 |
| Mollusca | Gastropoda | Lottioidea (sf) | Lepetidae | 0.1 ± 0.3 | 0 | 0.5 ± 1.2 |
| Mollusca | Gastropoda | Lottioidea (sf) | Nacellidae | 3.8 ± 2.8 | 4.4 ± 4.9 | 0.8 ± 1.7 |
| Mollusca | Gastropoda | Neogastropoda | Mangeliidae | 0 | 0 | 0 |
| Mollusca | Gastropoda | Trochaclididae (f) | Trochaclididae | 0.8 ± 0.8 | 0.2 ± 0.4 | 0.4 ± 0.7 |
| Mollusca | Gastropoda | Trochoidea (sf) | Calliostomatidae | 0.1 ± 0.3 | 0.2 ± 0.7 | 0 |
| Mollusca | Polyplacophora | Chitonida | Ischnochitonidae | 0 | 0 | 0.1 ± 0.3 |
| Nemertea | Anopla | Lineidae (f) | Lineidae | 0.3 ± 0.7 | 0 | 0 |
| Nemertea | Anopla | Monostilifera | non Lineidae Indet. | 0.6 ± 1.3 | 0.4 ± 0.7 | 1.3 ± 1.3 |
|  |  |  |  |  |  |  |
|  |  |  |  |  |  |  |
| Average number of individuals m^-2^ for each site. | | |  | Sites D-F Hangar Cove. | |  |
| **Phylum** | **Class** | **Order (where there is no order f:family, sf:superfamily)** | **Family** | **D** | **E** | **F** |
| Annelida | Polychaeta | Capitellidae (f) | Capitellidae | 0 | 0.1 ± 0.4 | 0 |
| Annelida | Polychaeta | Eunicida | Dorvilleidae | 0.1 ± 0.3 | 0 | 0 |
| Annelida | Polychaeta | Maldanidae (f) | Maldanidae | 0.7 ± 1.1 | 0.1 ± 0.4 | 0 |
| Annelida | Polychaeta | Opheliida | Opheliidae | 6.2 ± 8.7 | 6.9 ± 13.2 | 2.3 ± 1.5 |
| Annelida | Polychaeta | Orbiniida | Orbiniidae | 98.6 ± 69.1 | 97.8 ± 61.6 | 41 ± 6.1 |
| Annelida | Polychaeta | Phyllodocida | Nephtyidae | 15 ± 9.8 | 10.9 ± 4.8 | 3.3 ± 1.2 |
| Annelida | Polychaeta | Phyllodocida | Nereididae | 0 | 0 | 0 |
| Annelida | Polychaeta | Phyllodocida | Phyllodocidae | 0 | 0.1 ± 0.4 | 0 |
| Annelida | Polychaeta | Phyllodocida | Polynoidae | 3.4 ± 3 | 2.9 ± 3.7 | 0.3 ± 0.6 |
| Annelida | Polychaeta | Phyllodocida | Syllidae | 0 | 0.1 ± 0.4 | 0 |
| Annelida | Polychaeta | Polygordiidae (f) | Polygordiidae | 3 ± 7.8 | 0 | 0 |
| Annelida | Polychaeta | Spionida | Apistobranchidae | 1.8 ± 3 | 6.8 ± 10.5 | 0.3 ± 0.6 |
| Annelida | Polychaeta | Spionida | Spionidae | 0 | 0 | 0 |
| Annelida | Polychaeta | Terebellida | Ampharetidae | 0.1 ± 0.3 | 0 | 0 |
| Annelida | Polychaeta | Terebellida | Cirratulidae | 218.4 ± 100.2 | 128.4 ± 104.6 | 266.3 ± 144.5 |
| Annelida | Polychaeta | Terebellida | Terebellidae | 0.5 ± 0.8 | 3.9 ± 4 | 2.7 ± 1.5 |
| Arthropoda | Malacostraca | Amphipoda | Amphilochidae | 0 | 0 | 0 |
| Arthropoda | Malacostraca | Amphipoda | Calliopiidae | 1.2 ± 1.9 | 0.4 ± 1.1 | 6.7 ± 9.1 |
| Arthropoda | Malacostraca | Amphipoda | Exoedicerotidae | 0.4 ± 0.7 | 0.3 ± 0.7 | 0.7 ± 1.2 |
| Arthropoda | Malacostraca | Amphipoda | Lysianassidae | 0.2 ± 0.4 | 11.8 ± 31.2 | 0.7 ± 1.2 |
| Arthropoda | Malacostraca | Amphipoda | Oedicerotidae | 38.6 ± 28.6 | 21.9 ± 9.5 | 56 ± 86 |
| Arthropoda | Malacostraca | Amphipoda | Phoxocephalidae | 0.4 ± 0.7 | 0.1 ± 0.4 | 0 |
| Arthropoda | Malacostraca | Amphipoda | Pontogeneiidae | 6.9 ± 8.9 | 0.9 ± 1.5 | 57.3 ± 66.2 |
| Arthropoda | Malacostraca | Amphipoda | Stenothoidae | 0 | 0 | 0 |
| Arthropoda | Malacostraca | Cumacea | Bodotriidae | 3.6 ± 3.9 | 5.5 ± 5 | 2.3 ± 1.5 |
| Arthropoda | Malacostraca | Cumacea | Diastylidae | 0 | 0 | 0 |
| Arthropoda | Malacostraca | Cumacea | Leuconidae | 0.6 ± 1.6 | 4.3 ± 3.8 | 0.7 ± 1.2 |
| Arthropoda | Malacostraca | Cumacea | Nannastacidae | 1.4 ± 2.3 | 2.3 ± 4.2 | 0 |
| Arthropoda | Malacostraca | Euphausiacea | Euphausiidae | 0 | 0.1 ± 0.4 | 0 |
| Arthropoda | Malacostraca | Isopoda | Gnathiidae | 0.1 ± 0.3 | 0 | 0 |
| Arthropoda | Malacostraca | Isopoda | Janiridae | 0 | 0.3 ± 0.5 | 0 |
| Arthropoda | Malacostraca | Isopoda | Sphaeromatidae | 0 | 0 | 0 |
| Arthropoda | Malacostraca | Mysida | Mysidae | 0 | 0 | 0 |
| Arthropoda | Malacostraca | Tanaidacea | Nototanaidae | 8.6 ± 9.4 | 5.1 ± 5.7 | 42.7 ± 38.5 |
| Arthropoda | Ostracoda | Myodocopida | Philomedidae | 8.8 ± 7.5 | 10 ± 4.2 | 0.3 ± 0.6 |
| Cephalorhyncha | Priapulida | Priapulidae (f) | Priapulidae | 6.6 ± 5.4 | 3.9 ± 3.3 | 10.3 ± 5.9 |
| Chordata | Actinopterygii | Perciformes | Artedidraconidae | 0 | 0 | 0 |
| Chordata | Actinopterygii | Perciformes | Nototheniidae | 0 | 0 | 0.3 ± 0.6 |
| Cnidaria | Anthozoa | Actiniaria | Edwardsiidae | 209.7 ± 207.5 | 71.5 ± 119 | 23 ± 33 |
| Echinodermata | Asteroidea | Forcipulatida | Asteriidae | 0 | 0 | 0 |
| Echinodermata | Asteroidea | Valvatida | Odontasteridae | 0.1 ± 0.3 | 0 | 0 |
| Echinodermata | Echinoidea | Camarodonta | Echinidae | 11.9 ± 9.6 | 20.9 ± 16.1 | 8 ± 6.2 |
| Echinodermata | Holothuroidea | Dendrochirotida | Cucumariidae | 0 | 0.1 ± 0.4 | 0.3 ± 0.6 |
| Echinodermata | Holothuroidea | Dendrochirotida | Psolidae | 0 | 0.1 ± 0.4 | 0 |
| Echinodermata | Ophiuroidea | Ophiurida | Ophiuridae | 2 ± 1.3 | 2.4 ± 1.2 | 3.7 ± 1.5 |
| Mollusca | Bivalvia | Anomalodesmata | Laternulidae | 11.9 ± 6.8 | 13.4 ± 17.6 | 4.7 ± 2.5 |
| Mollusca | Bivalvia | Arcoida | Philobryidae | 0 | 0 | 0 |
| Mollusca | Bivalvia | Lucinoida | Thyasiridae | 3.1 ± 2.2 | 2.8 ± 3.1 | 4 ± 3.6 |
| Mollusca | Bivalvia | Nuculanoida | Yoldiidae | 243.6 ± 106.8 | 158.6 ± 86.1 | 167.3 ± 112.9 |
| Mollusca | Bivalvia | Veneroida | Cyamiidae | 2.9 ± 3.7 | 43.3 ± 43.2 | 0 |
| Mollusca | Bivalvia | Veneroida | Montacutidae | 1535.1 ± 993.2 | 1449.3 ± 828 | 576.7 ± 506.6 |
| Mollusca | Gastropoda | Littorinimorpha | Eatoniellidae | 0 | 0 | 0 |
| Mollusca | Gastropoda | Littorinimorpha | Littorinidae | 0 | 0.1 ± 0.4 | 0 |
| Mollusca | Gastropoda | Littorinimorpha | Rissoidae | 1.2 ± 2.3 | 0.6 ± 0.7 | 0.3 ± 0.6 |
| Mollusca | Gastropoda | Lottioidea (sf) | Lepetidae | 0 | 0.1 ± 0.4 | 0 |
| Mollusca | Gastropoda | Lottioidea (sf) | Nacellidae | 0.2 ± 0.4 | 0.3 ± 0.5 | 0 |
| Mollusca | Gastropoda | Neogastropoda | Mangeliidae | 0.9 ± 1.1 | 0 | 0 |
| Mollusca | Gastropoda | Trochaclididae (f) | Trochaclididae | 0.1 ± 0.3 | 0 | 0 |
| Mollusca | Gastropoda | Trochoidea (sf) | Calliostomatidae | 0 | 0 | 0 |
| Mollusca | Polyplacophora | Chitonida | Ischnochitonidae | 0 | 0 | 0 |
| Nemertea | Anopla | Lineidae (f) | Lineidae | 0.5 ± 0.7 | 0.6 ± 1.8 | 0.7 ± 0.6 |
| Nemertea | Anopla | Monostilifera | non Lineidae Indet. | 0.2 ± 0.6 | 0.5 ± 1.1 | 0 |

Table S1. Average number of individuals 0.25m^-2^ collected at each site (South Cove, A-C; Hangar Cove, D-F).
