# Supplementary material for: Spatial and temporal dynamics of Antarctic shallow soft-bottom benthic communities: ecological drivers under climate change
Source: BMC Ecol. 2019 Jul 1;19:27. doi: 10.1186/s12898-019-0244-x (PMC6604130; doi:10.1186/s12898-019-0244-x)
Supplement: Supplementary file 2 — Additional file 2: Table S2. The date, position and water depth of samples taken in summer and winter. The square of the IBIS (iceberg disturbance) grid associated with the sample, is also reported.* indicates that the position was calculated from position from previous samples. Figure S1. Non-metric multidimensional scaling (nMDS) plots based on the Bray–Curtis similarity matrix for abundance of Mollusca, identified to the genus level. Benthic assemblages at South Cove, circles (Sites A, B and C) and Hangar Cove, triangles (Sites D, E and F). The low stress value of 0.15 indicates this plot is a good two-dimensional representation of the community in multi-dimensional space. Clustering shown at a similarity level of 50%. Description of outlier, Hangar Cove, site E. Table S3. Meta-analysis of the abundance (density, m-3) of sediment communities across latitudes. Abundance from samples using 1 mm mesh were doubled to compare with those from 0.5 mm mesh (following Arrigo and Dijken [63]). Reference lists for abundance and species richness mata-analyses. [file 12898_2019_244_MOESM2_ESM.docx]

| **Site** | **Sampling Date** | **Summer/Winter** | **IBIS grid square sampled** | **Lat** | **Lon** | **Depth** |
| --- | --- | --- | --- | --- | --- | --- |
| HangarA | 2013-01-30 | S |  | 67.33.833 | 68.07.546 | 16.7 |
| SCoveC | 2013-02-21 | S |  | 67.34.152 | 68.08.019 | 19.7 |
| SCoveC | 2013-03-18 | S |  | 67 34.150* | 68 08.016 | 20.4 |
| HangarA | 2013-04-29 | S |  | 67.33.819 | 68.07.522 | 19.2 |
| SCoveA | 2013-05-22 | S |  | 67.34.173 | 68.07.977 | 18.1 |
| HangarB | 2013-05-28 | S |  | 67.33.815 | 68.07.524 | 19 |
| SCoveA | 2014-01-08 | S |  | 67.34.168 | 68.07.968 | 18.1 |
| SCoveC | 2014-01-15 | S |  | 67.34.152 | 68.08.008 | 19.1 |
| SCoveA | 2014-01-21 | S |  | 67.34.172 | 68.07.978 | 19.3 |
| HangarA | 2014-01-27 | S |  | 67.33.818 | 68.07.541 | 17.9 |
| HangarA | 2014-02-04 | S |  | 67.33.830 | 68.07.539 | 16.4 |
| SCoveB | 2014-02-19 | S | C3 | 67.34.153 | 68.07.956 | 15.1 |
| HangarA | 2014-02-25 | S |  | 67.33.833 | 68.07.546 | 14.9 |
| SCoveB | 2014-03-04 | S | B2 | 67.34.153 | 68.07.956 | 14.7 |
| SCoveA | 2014-03-11 | S |  | 67.34.169 | 68.07.976 | 20.4 |
| SCoveB | 2014-03-22 | S | F2 | 67.34.153 | 68.07.956 | 15 |
| SCoveC | 2014-03-26 | S | F2 | 67.34.153 | 68.08.045 | 22.2 |
| SCoveC | 2014-03-28 | S | D6 | 67.34.153 | 68.08.045 | 21.5 |
| HangarA | 2014-04-16 | S | B3 | 67.33.833 | 68.07.546 | 15.3 |
| SCoveC | 2014-04-26 | S | A1 | 67.34.154 | 68.08.046 | 21.6 |
| SCoveB | 2014-05-13 | S | C5 | 67.34.153 | 68.07.956 | 14.9 |
| SCoveB | 2014-05-20 | S | E5 | 67.34.153 | 68.07.956 | 14.3 |
| HangarB | 2014-05-22 | S | E2 | 67.33.820 | 68.07.524 | 19.9 |
| SCoveA | 2014-06-09 | W | E5 | 67.34.170 | 68.07.977 | 18.7 |
| SCoveC | 2014-06-16 | W | F6 | 67.34.156 | 68.08.048 | 22.2 |
| SCoveB | 2014-06-27 | W | A1 | 67.34.153 | 68.07.956 | 14.8 |
| SCoveC | 2014-07-15 | W | A5 | 67.34.155 | 68.08.047 | 21.6 |
| SCoveA | 2014-07-23 | W | A2 | 67.34.171 | 68.07.978 | 18.7 |
| HangarA | 2014-08-18 | W | F6 | 67.33.833 | 68.07.546 | 15.7 |
| HangarA | 2014-08-26 | W | E3 | 67.33.833 | 68.07.546 | 14.8 |
| HangarB | 2014-09-01 | W | A2 | 67.33.821 | 68.07.525 | 19.9 |
| HangarB | 2014-09-24 | W | E5 | 67.33.822 | 68.07.526 | 19.6 |
| SCoveA | 2014-09-26 | W | F3 | 67.34.172 | 68.07.979 | 18.6 |
| SCoveB | 2014-10-27 | S | A3 | 67.34.153 | 68.07.956 | 14.9 |
| SCoveA | 2014-11-24 | S | B1 | 67.34.169 | 68.07.976 | 17.6 |
| SCoveC | 2014-11-28 | S | D3 | 67.34.153 | 68.08.045 | 21.8 |
| SCoveC | 2014-12-03 | S | A3 | 67.34.153 | 68.08.045 | 21.8 |
| SCoveA | 2014-12-16 | S | A4 | 67.34.169 | 68.07.976 | 18.2 |
| HangarB | 2014-12-18 | S | B4 | 67.33.820 | 68.07.524 | 18.7 |
| SCoveB | 2015-01-21 | S | F4 | 67.34.153 | 68.07.956 | 14.5 |
| SCoveA | 2015-01-26 | S | F1 | 67.34.169 | 68.07.976 | 18.3 |
| SCoveB | 2015-02-02 | S | D4 | 67.34.153 | 68.07.956 | 13.6 |
| SCoveC | 2015-02-09 | S | B4 | 67.34.153 | 68.08.045 | 22.4 |
| HangarC | 2015-02-23 | S | E4 | 67.33.822 | 68.07.587 | 16.8 |
| HangarC | 2015-02-26 | S | A3 | 67.33.822 | 68.07.587 | 16.8 |
| HangarA | 2015-03-03 | S | A2 | 67.33.833 | 68.07.546 | 15 |
| HangarA | 2015-03-04 | S | C1 | 67.33.833 | 68.07.546 | 14.8 |
| HangarA | 2015-03-10 | S | A6 | 67.33.833 | 68.07.546 | 15.3 |
| HangarB | 2015-03-11 | S | E1 | 67.33.820 | 68.07.524 | 19.9 |
| HangarB | 2015-03-18 | S | E6 | 67.33.820 | 68.07.524 | 19.9 |
| HangarC | 2015-03-27 | S | D3 | 67.33.822 | 68.07.587 | 16.4 |

Table S2. The date, position and water depth of samples taken in summer and winter. The square of the IBIS (iceberg disturbance) grid associated with the sample, is also reported.* indicates that the position was calculated from position from previous samples.

Figure S1. Non-metric multidimensional scaling (nMDS) plots based on the Bray-Curtis similarity matrix for abundance of Mollusca, identified to the genus level. Benthic assemblages at South Cove, circles (Sites A, B and C) and Hangar Cove, triangles (Sites D, E and F). The low stress value of 0.15 indicates this plot is a good two-dimensional representation of the community in multi-dimensional space. Clustering shown at a similarity level of 50%.

Description of outlier Hangar Cove, site E.

In terms of community composition, one sample from Hangar Cove, site E, sampled on 29/04/13 was clearly separated as an outlier (Fig. 2). This sample had relatively low abundance of the dominant infauna compared to the other samples from this cove, in particular the bivalves *Altenaeum charcoti* (Montacutidae) and *Aequiyoldia eightsi* (Yoldiidae), a low abundance of all polychaetes including a complete absence of Cirratulidae and Edwardsiidae burrowing anemones. In contrast it contained very high numbers of the amphipod *Cheirimedon femoratus* (family Lysianassidae); 80% of *Cheirimedon femoratus* found within the 51 samples collected for this study were found in this one sample (89 out of a total of 111 individuals). The sample also contained relatively high numbers of the urchin *Sterechinus neumayeri*. The sediment thickness of this quadrat was one of the shallowest, at only 25 mm. There was no evidence of recent iceberg disturbance close to this grid.

| Location | Latitude | Density.m^-3^ | Mesh size | Water depth/m | Reference | Source |
| --- | --- | --- | --- | --- | --- | --- |
| McMurdo Sound East | -78 | 118712 | 0.5 | 20 | 26 | 25 |
| McMurdo Sound East | -78 | 155573 | 0.5 | 20 | 26 | 25 |
| McMurdo Sound East | -78 | 145781 | 0.5 | 30 | 26 | 25 |
| McMurdo Sound West | -78 | 2184 | 0.5 | 30 | 26 | 25 |
| McMurdo Sound West | -78 | 45294 | 0.5 | 30 | 26 | 25 |
| Rothera Point, Atarctica | -67 | 5400 | 1 | 18 | Current Study |  |
| Brown Bay, Casey Antarctica | -66 | 20377.6 | 1 | 10 | 29 |  |
| Shanon Bay, Casey Antarctica | -66 | 17417.6 | 1 | 17.5 | 29 |  |
| O'Brien Bay, Casey, Antarctica | -66 | 18162.4 | 1 | 25 | 29 |  |
| Beall Island, Casey, Antarctica | -66 | 30856 | 1 | 25 | 29 |  |
| Arthur harbour, Anvers Island, Bismark strait | -64 | 36824 | 1 | 40 | 73 | 25 |
| Arthur harbour, Anvers Island | -64 | 36824 | 1 | 30 | 74 | 25 |
| Admiralty Bay, Antarctica | -62 | 9533 | 0.5 | 17 | 43 |  |
| Admiralty Bay, Antarctica | -62 | 41696.4286 | 0.5 | 25 | 75 |  |
| Admiralty Bay, Antarctica | -62 | 15000 | 0.2 | 22 | 76 |  |
| Admiralty Bay, Antarctica | -62 | 5800 | 0.5 | 10 | 29 |  |
| King Edward Cove, S. Georgia | -54 | 3236 | 1 | 11 | 27 | 25 |
| Bass Strait, Australia | -40 | 5794.03846 |  | 31 | 78 | 79 |
| Bass Strait | -40 |  | 1 | 31 | 78 | 27 |
| Port Phillip Bay | -38 | 3634.27907 |  | 13 | 78 | 79 |
| Port Philip Bay | -38 |  |  | 13 | 78 | 27 |
| Hinchinbrook Island, Australia | -18 | 2250 | 0.5 | 15 | 80 |  |
| Java | -6 | 360 | 0.5 | 30 | 81 |  |
| Topical, Kingston Harbour, Jamaica | 17 | 3840 | 0.5 | 11 | 82 | 79 |
| Subtropical Hong Kong | 22 | 101.42 |  | 18 | 79 |  |
| South Wales | 51 | 2284 | 0.5 | 14 | 81 |  |
| South Wales | 51 | 4231 | 0.5 | 13.5 | 81 |  |
| Lochs Linnhe and Eil | 56 | 3480 | 1 | 60 | 83 | 27 |
| Lochs Etive, Creran | 56 | 3363.63636 | 1 | 70 | 84 | 27 |
| Swedish fjords, above halocline | 58 | 10164.0909 | 1 | 11 | 85 | 79 |
| Swedish fjords, below halocline | 58 | 20242.5 | 1 | 30 | 85 | 79 |
| Frigg oilfield, Norway | 59 | 3872.01333 |  | 70 | 78 | 79 |
| Disk Bugt, shallow | 69 | 2598 | 1 |  | 86 | 25 |
| McBeth, Bafin Island | 69 | 836 | 2 | 20 | 87 |  |
| McBeth, Bafin Island | 69 | 4704 | 2 | 20 | 87 |  |
| Itirbilung, Bafin Island | 69 | 788 | 2 | 55 | 87 |  |
| Cambridge fjord, Bafin Island | 71 | 484 | 2 | 19 | 87 |  |
| E Greenland | 74 | 2700 | 0.5 | 20 | 28 |  |
| Spitzbergen | 77 | 3613.46154 | 1 | 63 | 88 |  |
| Spitzbergen | 78 | 8420 | 0.5 | 30 | 89 |  |

Table S3: Meta-analysis of the abundance (density, m^-3^) of sediment communities across latitudes. Abundance from samples using 1mm mesh were doubled to compare with those from 0.5mm mesh (following White [25]).

References for abundance meta-analysis.

25. White MG Marine benthos, In Laws RM editor. Antarctic Ecology Volume 2. London: Academic Press. 1984. P 421-461.

26. Dayton PK, Oliver JS.“Antarctic Soft-bottom Benthos in Oligotrophic and Eutrophic Environments”. Science, 1977;197:55–58.

27. Gray JS. Antarctic marine benthic biodiversity in a world-wide latitudinal context. Polar Biol. 2001;24:633-641. https://doi.org/10.1007/s003000100244.

28. Sjer MK, Thomas Jensen K, Rysgaard S. Macrozoobenthic community structure in a high-arctic East Greenland fjord. Polar Biology, 2000;23:792-801.

29. Sicinski J, Rózycki O, Kittel W, Zoobenthos and zooplankton of Herve Cove, King George Island, South Shetland Islands, Antarctic. Pol Polar Res. 1996;17:221-238.

30. Stark JS. The distribution and abundance of soft-sediment macrobenthos around Casey Station, East Antarctica. Polar Biol. 2000;23:840-850.

43. Siciński J, Pabis K, Jażdżewski K, Konopacka A, Błażewicz-Paszkowycz M. Macrozoobenthos of two Antarctic glacial coves: a comparison with non-disturbed bottom areas. Polar Biol. 2012;35:355-367.

73. Richardson MG, Hedgpeth JW. Antarctic soft-bottom, microbenthic community adaptations to a cold, stable, highly productive glacially affected environment, In: Llano GA, editor. Adaptations within Antarctic Ecosystems. Proc. 3rd SCAR symp. Antarctic Biology, Houston: Gulf Publishing Company. 1977. p. 181-196.

74. Lowry JK. Soft bottom microbenthic community of Arthur Harbour, Antarctica. Ant Res Ser 1975;23:1-19.

75. Echeverria CA, Paiva PC (2006) Macrofaunal shallow benthic communities along a discontinuous annual cycle at Admiralty Bay, King George Island, Antarctica. Polar Biol. 2006;29:263-269.

76. Kern Y, Rodrigues AR, Absher TM. Colonization of soft sediments by benthic communities: an experimental approach in Admiralty Bay, King George Island. J Exp Mar Biol Ecol, 2014;453:1-12.

77. Platt HM. Sedimentation and the distribution of organic matter in a sub-Antarctic marine bay. Estuar Coast Mar Sci. 1979;9:51-63.

78. Gray JS, Poore GCB, Ugland KI, Wilson RS, Olsgard F, Johannessen Ø. Coastal and deep-sea benthic diversities compared. Mar Ecol Prog Ser 1997;159:97-103.

79. Gray JS. Species richness of marine soft sediments. Marine Ecology Progress Series, 2002;244:285–297.

80. Alongi DM, Christoffersen P. Benthic infauna and organism-sediment relations in a shallow, tropical coastal area: influence of outwelled mangrove detritus and physical disturbance. Mar Ecol Prog Ser. 1992;81:229-245.

81. Warwick RM, Ruswahyuni Comparative study of the structure of some tropical and temperate marine soft-bottom microbenthic communities. Mar Biol. 1987;95:641.

82. Wade BA. A description of a highly diverse soft-bottom community in Kingston Harbour, Jamaica. Mar Biol 1972;13:57–69.

83. Pearson TH. The benthic ecology of Loch Linnhe and Loch Eil, a sea-loch system on the west coast of Scotland. I. The physical environment and the distribution of the macrobenthic fauna. J Exp Mar Biol Ecol. 1970;5:1-34.

84. Gage JD. Community structure of the benthos in Scottish sealochs. I. Intorductionand species diversity. Mar Biol 1993;14:281-297.

85. Rosenberg R, Möller P. Salinity stratified benthic macrofaunal communities and long-term monitoring along the west coast of Sweden. J Exp Mar Biol Ecol. 1979;37:175–203.

86. Ellis DV Marine infaunal benthos in Arctic North America. Arct Inst North Am Tech Pap 1960;5:1-53.

87. Syvitski JPM, Farrow GE, Atkinson RJA, Moore PG, Andrews JT. Baffin Island Fjord Macrobenthos: bottom communities and environmental signficance. Arctic 1989;42:232-247.

88. Renaud PE, Webb TJ, Bjorgesaeter A, Karakassis I, Kedra M, Kendall A et al. Continental-scale patterns in benthic invertebrate diversity: insights from the MacroBen database. Mar Ecol Prog Ser, 2009;382:239-252.

89. Kendall MA, Aschan M. Latitudinal gradients in the structure of macrobenthic communities: a comparison of Arctic, temperate and tropical sites. J Exp Mar Biol Ecol. 1993;172:157-169.

References for species richness meta-analysis

28. Sjer MK, Thomas Jensen K, Rysgaard S. Macrozoobenthic community structure in a high-arctic East Greenland fjord. Polar Biology, 2000;23:792-801.

29. Sicinski J, Rózycki O, Kittel W, Zoobenthos and zooplankton of Herve Cove, King George Island, South Shetland Islands, Antarctic. Pol Polar Res. 1996;17:221-238.

30. Stark JS. The distribution and abundance of soft-sediment macrobenthos around Casey Station, East Antarctica. Polar Biol. 2000;23:840-850.

43. Siciński J, Pabis K, Jażdżewski K, Konopacka A, Błażewicz-Paszkowycz M. Macrozoobenthos of two Antarctic glacial coves: a comparison with non-disturbed bottom areas. Polar Biol. 2012;35:355-367.

79. Gray JS. Species richness of marine soft sediments. Marine Ecology Progress Series, 2002;244:285–297.

83. Pearson TH. The benthic ecology of Loch Linnhe and Loch Eil, a sea-loch system on the west coast of Scotland. I. The physical environment and the distribution of the macrobenthic fauna. J Exp Mar Biol Ecol. 1970;5:1-34.

85. Rosenberg R, Möller P. Salinity stratified benthic macrofaunal communities and long-term monitoring along the west coast of Sweden. J Exp Mar Biol Ecol. 1979;37:175–203.

88. Renaud PE, Webb TJ, Bjorgesaeter A, Karakassis I, Kedra M, Kendall A et al. Continental-scale patterns in benthic invertebrate diversity: insights from the MacroBen database. Mar Ecol Prog Ser, 2009;382:239-252.

89. Kendall MA, Aschan M. Latitudinal gradients in the structure of macrobenthic communities: a comparison of Arctic, temperate and tropical sites. J Exp Mar Biol Ecol. 1993;172:157-169.

90. Barnes DKAB, Brockington S. Zoobenthic biodiversity, biomass and abundance at Adeleide Island, Antarctica. Mar Ecol Prog Ser. 2003;249:145-155.
