# Supplementary material for: Spatial and temporal dynamics of Antarctic shallow soft-bottom benthic communities: ecological drivers under climate change
Source: BMC Ecol. 2019 Jul 1;19:27. doi: 10.1186/s12898-019-0244-x (PMC6604130; doi:10.1186/s12898-019-0244-x)
Supplement: Supplementary file 3 — Additional file 3: Table S4. Summary results of the metabarcoding in silico analysis for both markers tested (COI and 18S rRNA) using QIIME2. Total number of raw reads and reads after each step in the pipelines are shown as well as total number of Amplicon Sequence Variants (ASVs) after running Qiime2. ASVs assigned to both Eukaryotes and Metazoa are shown for the different BLAST sequence identity matches (90%, 95% and 97%) and also Not assigned ASVs for both markers using 95% BLAST match. Shaded grey; data used for ecological patterns and community profiles at higher taxonomic ranking. (*) ASVs used to compare species identification detected by eDNA and morphology. Amplicon Sequence Variants; ASVs. [file 12898_2019_244_MOESM3_ESM.docx]

| Stats MiSeq | 18S | COI |
| --- | --- | --- |
| raw reads | 4283138 | 8864734 |
| filtered | 3748469 | 8427528 |
| denoised | 3748469 | 8427528 |
| merged | 3666942 | 8385200 |
| non-chimeric | 3463925 | 8295561 |
| ASVs | 3007 | 11698 |
| Eukaryotes ASVs assigned at 90% BLAST match | 2553 | 791 |
| Eukaryotes ASVs assigned at 95% BLAST match | 1854 | 443 |
| Eukaryotes ASVs assigned at 97% BLAST match | 1358 | 323 |
| Metazoa ASVs assigned at 90% BLAST match | 273 | 618 |
| Metazoa ASVs assigned at 95% BLAST match | 189 | 347 |
| Metazoa ASVs assigned at 97% BLAST match | 127 | 249* |
| Not Assigned ASVs at 95% BLAST match | 1346 | 13271 |
|  |  |  |

**Table S4-** Summary results of the metabarcoding in silico analysis for both markers tested (COI and 18S rRNA) using QIIME2. Total number of raw reads and reads after each step in the pipelines are shown as well as total number of Amplicon Sequence Variants (ASVs) after running Qiime2. ASVs assigned to both Eukaryotes and Metazoa are shown for the different BLAST sequence identity matches (90%, 95% and 97%) and also Not assigned ASVs for both markers using 95% BLAST match. Shaded grey; data used for ecological patterns and community profiles at higher taxonomic ranking. (*) ASVs used to compare species identification detected by eDNA and morphology. Amplicon Sequence Variants; ASVs.
