# Supplementary material for: Spatial and temporal dynamics of Antarctic shallow soft-bottom benthic communities: ecological drivers under climate change
Source: BMC Ecol. 2019 Jul 1;19:27. doi: 10.1186/s12898-019-0244-x (PMC6604130; doi:10.1186/s12898-019-0244-x)
Supplement: Supplementary file 4 — Additional file 4: Table S5. eDNA species list retrieved using the COI marker using a 97% sequence BLAST match against GenBank database. First column shows ASVs identification tag, followed by number of reads allocated to each ASVs per sample site (HC1, HC2, HC3) and the last seven columns show the taxonomy ranking to each ASVs from Kingdom to species, respectively. Hangar Cove replicates 1–3 (HC1, HC2, HC3) and South Cove replicates 1–3 (SC1-3). [file 12898_2019_244_MOESM4_ESM.docx]

| #ASV ID | HC1 | HC2 | HC3 | Superkingdom | Kingdom | Phylum | Class | Order | Family | Genus | Species |
| --- | --- | --- | --- | --- | --- | --- | --- | --- | --- | --- | --- |
| f4940b9aa86f03f3f222b68e80f5abb8 | 0 | 0 | 0 | Eukaryota | Metazoa | Arthropoda | Insecta | Coleoptera | Chrysomelidae | Agelastica | Agelastica coerulea |
| a8fbcdd964d02e437d7c349c33ef46e4 | 0 | 0 | 0 | Eukaryota | Metazoa | Annelida | Polychaeta | Phyllodocida | Nephtyidae | Aglaophamus | Aglaophamus dibranchis |
| 25ca4a2cc56daffb4f7358070876a7f7 | 0 | 0 | 0 | Eukaryota | Metazoa | Annelida | Polychaeta | Phyllodocida | Nephtyidae | Aglaophamus | Aglaophamus trissophyllus |
| 566efce7f80634ef733a156d7897aa48 | 0 | 0 | 0 | Eukaryota | Metazoa | Annelida | Polychaeta | Phyllodocida | Nephtyidae | Aglaophamus | Aglaophamus trissophyllus |
| 6fe8a6e5e146131488f1f71c009bd93b | 63 | 0 | 0 | Eukaryota | Metazoa | Annelida | Polychaeta | Phyllodocida | Nephtyidae | Aglaophamus | Aglaophamus trissophyllus |
| 8b60eaaa89e01572fbb88a5c810d4725 | 128 | 157 | 0 | Eukaryota | Metazoa | Annelida | Polychaeta | Phyllodocida | Nephtyidae | Aglaophamus | Aglaophamus trissophyllus |
| 54365498f436f498c61b93f369620772 | 0 | 0 | 0 | Eukaryota | Metazoa | Chordata | Amphibia | Anura | Dendrobatidae | Allobates | Allobates femoralis |
| 090c761191703bb1fbe9aedbaa249ebf | 0 | 0 | 0 | Eukaryota | Metazoa | Annelida | Clitellata | Haplotaxida | Lumbricidae | Allolobophora | Allolobophora chlorotica |
| 1a8056778c5201f4a87e8174689b1681 | 0 | 0 | 0 | Eukaryota | Metazoa | Annelida | Clitellata | Haplotaxida | Lumbricidae | Allolobophora | Allolobophora chlorotica |
| a3a92718e2c2c66253f67a931d71b459 | 0 | 0 | 0 | Eukaryota | Metazoa | Annelida | Clitellata | Haplotaxida | Lumbricidae | Allolobophora | Allolobophora chlorotica |
| 059a2b2e2ad5353094280cc6145d5743 | 0 | 0 | 0 | Eukaryota | Metazoa | Arthropoda | Insecta | Psocoptera | Psocidae | Amphigerontia | Amphigerontia contaminata |
| e69436c3a62912d6f93aa160bbbf643f | 0 | 0 | 0 | Eukaryota | Metazoa | Arthropoda | Insecta | Psocoptera | Psocidae | Amphigerontia | Amphigerontia contaminata |
| 649429954022cf584069b329535be8a8 | 0 | 0 | 0 | Eukaryota | Metazoa | Arthropoda | Insecta | Hymenoptera | Andrenidae | Andrena | Andrena hirticincta |
| b9c18ede9eddb649dbc5bf6c987819bd | 6 | 0 | 0 | Eukaryota | Metazoa | Bryozoa | Gymnolaemata | Cheilostomatida | Hippothoidae | Antarctothoa | Antarctothoa sp. 'polystachya' |
| 8405259960ffe8c1f96ffb7e3b266b7f | 0 | 0 | 0 | Eukaryota | Metazoa | Arthropoda | Insecta | Coleoptera | Scarabaeidae |  | Aphodiinae sp. BOLD:AAN5996 |
| 9c89f86603d5009aaad1ba263a1f2432 | 0 | 0 | 0 | Eukaryota | Metazoa | Arthropoda | Branchiopoda | Anostraca | Artemiidae | Artemia | Artemia frameshifta |
| c15aa075c6540ccafed8d96fffbac14d | 0 | 0 | 0 | Eukaryota | Metazoa | Arthropoda | Branchiopoda | Anostraca | Artemiidae | Artemia | Artemia frameshifta |
| e8e2d1c0958ffd1fe66b0a025e2b5645 | 117 | 0 | 0 | Eukaryota | Metazoa | Arthropoda | Branchiopoda | Anostraca | Artemiidae | Artemia | Artemia frameshifta |
| 8b788ddb9e2a3756faa6d951778cd55d | 0 | 0 | 0 | Eukaryota | Metazoa | Arthropoda | Insecta | Hymenoptera | Bethylidae | Austranesia | Austranesia sp. 17 IA-2018 |
| e7ebc5995c645793172dc81483dd0254 | 68 | 0 | 0 | Eukaryota | Metazoa | Arthropoda | Insecta | Coleoptera | Cerambycidae | Batocera | Batocera lineolata |
| a22ba1170c0e73063b45ea6c4a6f1880 | 0 | 0 | 0 | Eukaryota | Metazoa | Rotifera | Bdelloidea | o__ |  |  | Bdelloidea sp. nd |
| 05ef7552829e187ef8a04e28d2906c59 | 0 | 0 | 0 | Eukaryota | Metazoa | Mollusca | Bivalvia | Mytiloida | Mytilidae | Brachidontes | Brachidontes rodriguezii |
| 13f0ac391ccb0ff198728f4a6bbb1080 | 0 | 0 | 0 | Eukaryota | Metazoa | Mollusca | Bivalvia | Mytiloida | Mytilidae | Brachidontes | Brachidontes rodriguezii |
| 2fd28833b2028682aaf194384392aba9 | 0 | 0 | 0 | Eukaryota | Metazoa | Mollusca | Bivalvia | Mytiloida | Mytilidae | Brachidontes | Brachidontes rodriguezii |
| 30177e2d1ceea56c4cf6bfd00959febe | 0 | 0 | 0 | Eukaryota | Metazoa | Mollusca | Bivalvia | Mytiloida | Mytilidae | Brachidontes | Brachidontes rodriguezii |
| 46c8c5c9a419c628ad4d247b685f0180 | 0 | 0 | 0 | Eukaryota | Metazoa | Mollusca | Bivalvia | Mytiloida | Mytilidae | Brachidontes | Brachidontes rodriguezii |
| 4dc1520eeaed5ecb72f5b20bb3eaf9d4 | 0 | 0 | 0 | Eukaryota | Metazoa | Mollusca | Bivalvia | Mytiloida | Mytilidae | Brachidontes | Brachidontes rodriguezii |
| 74f4e1f5322b79500ff00a8b0fe3d240 | 0 | 0 | 0 | Eukaryota | Metazoa | Mollusca | Bivalvia | Mytiloida | Mytilidae | Brachidontes | Brachidontes rodriguezii |
| cee0814f80f9b069a35b42f0aa88de85 | 0 | 0 | 0 | Eukaryota | Metazoa | Mollusca | Bivalvia | Mytiloida | Mytilidae | Brachidontes | Brachidontes rodriguezii |
| e21b7ecf303ffeed0a482ef14fabae44 | 0 | 0 | 0 | Eukaryota | Metazoa | Mollusca | Bivalvia | Mytiloida | Mytilidae | Brachidontes | Brachidontes rodriguezii |
| f6059ad0632b151a911010c58d1a9c35 | 0 | 0 | 0 | Eukaryota | Metazoa | Mollusca | Bivalvia | Mytiloida | Mytilidae | Brachidontes | Brachidontes rodriguezii |
| dc466c8a8957cbe196b0a667bef0519d | 0 | 0 | 0 | Eukaryota | Metazoa | Arthropoda | Arachnida | Sarcoptiformes | Brachychthoniidae |  | Brachychthoniidae sp. BOLD:ABV0368 |
| 1aa2a7e7c9a95668503fc1dda7dfa56f | 0 | 0 | 0 | Eukaryota | Metazoa | Arthropoda | Insecta | Coleoptera | Staphylinidae | Cafius | Cafius australis |
| f81c6945ae7145b45090e0ed4fa32924 | 127 | 0 | 0 | Eukaryota | Metazoa | Arthropoda | Insecta | Coleoptera | Staphylinidae | Cafius | Cafius australis |
| a6382624a173c3484f3b498edfd2d4d3 | 0 | 0 | 0 | Eukaryota | Metazoa | Arthropoda | Insecta | Hymenoptera | Formicidae | Camponotus | Camponotus sp. MG053 |
| aefb5d3e7e9f41df356f9eb8ec52c7c4 | 15 | 0 | 0 | Eukaryota | Metazoa | Arthropoda | Insecta | Diptera | Cecidomyiidae |  | Cecidomyiidae sp. BIOUG20769-C11 |
| c081b2e5f59ab109fcc844d4fb68a54d | 0 | 0 | 0 | Eukaryota | Metazoa | Arthropoda | Insecta | Diptera | Cecidomyiidae |  | Cecidomyiidae sp. BIOUG20893-G01 |
| 1eee3338b6d31c85cb66ffac57b68f7c | 0 | 0 | 0 | Eukaryota | Metazoa | Arthropoda | Insecta | Diptera | Cecidomyiidae |  | Cecidomyiidae sp. BOLD:ACI6109 |
| 06c2426cae7ad3ae4d0fc770eaf1d7b3 | 0 | 0 | 0 | Eukaryota | Metazoa | Annelida | Polychaeta | Phyllodocida | Polynoidae |  | cf. Polynoidae sp. DH-2009 |
| c11da9bd886c7b94e90e6e53e1696826 | 0 | 0 | 0 | Eukaryota | Metazoa | Annelida | Polychaeta | Phyllodocida | Polynoidae |  | cf. Polynoidae sp. DH-2009 |
| 06bbc8763f28d54fa99a3b40b608c69f | 0 | 0 | 0 | Eukaryota | Metazoa | Annelida | Polychaeta | Terebellida |  |  | cf. Terebellida sp. DH-2009 |
| 3325153b1618fdca2bc68db6461b4672 | 0 | 0 | 0 | Eukaryota | Metazoa | Annelida | Polychaeta | Terebellida |  |  | cf. Terebellida sp. DH-2009 |
| 74f079fdb77e0b4c12995db488d5085e | 41 | 0 | 0 | Eukaryota | Metazoa | Arthropoda | Insecta | Diptera | Chamaemyiidae |  | Chamaemyiidae sp. BOLD:ACG2991 |
| 8c0953af4c7c2cdf5804561fab138af9 | 0 | 0 | 0 | Eukaryota | Metazoa | Arthropoda | Insecta | Lepidoptera | Gelechiidae | Chionodes | Chionodes fuscomaculella |
| 3a0018caf61a860e36d81efe4e4c8e8f | 0 | 0 | 7 | Eukaryota | Metazoa | Annelida | Polychaeta | Spionida | Cirratulidae | Cirratulus | Cirratulus balaenophilus |
| e487b48222433918829cf2c3c62602d8 | 0 | 16 | 0 | Eukaryota | Metazoa | Annelida | Polychaeta | Spionida | Cirratulidae | Cirratulus | Cirratulus balaenophilus |
| 0ae114a845b8f4e424cdd1d067cf46b0 | 0 | 0 | 0 | Eukaryota | Metazoa | Arthropoda | Insecta | Diptera | Pipunculidae | Clistoabdominalis | Clistoabdominalis digitatus |
| 117d4a205023aa705fedbb282b710da9 | 0 | 0 | 0 | Eukaryota | Metazoa | Arthropoda | Insecta | Diptera | Pipunculidae | Clistoabdominalis | Clistoabdominalis digitatus |
| 117df6f4d5cb03c38edcef2023c47a2e | 0 | 0 | 0 | Eukaryota | Metazoa | Arthropoda | Insecta | Diptera | Pipunculidae | Clistoabdominalis | Clistoabdominalis digitatus |
| 1214c13977f03e71b687d44dd8490ac2 | 0 | 0 | 0 | Eukaryota | Metazoa | Arthropoda | Insecta | Diptera | Pipunculidae | Clistoabdominalis | Clistoabdominalis digitatus |
| 143641fa87b430fdb5ae284e6801a1e2 | 0 | 0 | 0 | Eukaryota | Metazoa | Arthropoda | Insecta | Diptera | Pipunculidae | Clistoabdominalis | Clistoabdominalis digitatus |
| 1f0c8d243e9c6d4e13c143015a7c7e27 | 0 | 0 | 0 | Eukaryota | Metazoa | Arthropoda | Insecta | Diptera | Pipunculidae | Clistoabdominalis | Clistoabdominalis digitatus |
| 2b999bd334b7d3d4574b1c487b1f701b | 0 | 0 | 0 | Eukaryota | Metazoa | Arthropoda | Insecta | Diptera | Pipunculidae | Clistoabdominalis | Clistoabdominalis digitatus |
| 2e27351642e5e9746e7fac844ce5ef92 | 0 | 0 | 0 | Eukaryota | Metazoa | Arthropoda | Insecta | Diptera | Pipunculidae | Clistoabdominalis | Clistoabdominalis digitatus |
| 43e5d4af75ed085582fd4a890705457a | 0 | 0 | 0 | Eukaryota | Metazoa | Arthropoda | Insecta | Diptera | Pipunculidae | Clistoabdominalis | Clistoabdominalis digitatus |
| 45c15400a3f38775331b30b85f12155f | 0 | 0 | 0 | Eukaryota | Metazoa | Arthropoda | Insecta | Diptera | Pipunculidae | Clistoabdominalis | Clistoabdominalis digitatus |
| 45c5e6d4274b1e05782af82b30e4216e | 0 | 0 | 0 | Eukaryota | Metazoa | Arthropoda | Insecta | Diptera | Pipunculidae | istoabdominalis | Clistoabdominalis digitatus |
| 510e62b17ba5959ccfc603968baba4fa | 0 | 0 | 0 | Eukaryota | Metazoa | Arthropoda | Insecta | Diptera | Pipunculidae | Clistoabdominalis | Clistoabdominalis digitatus |
| 5941f34c0ff71498ea1b4b8034f2a003 | 0 | 0 | 0 | Eukaryota | Metazoa | Arthropoda | Insecta | Diptera | Pipunculidae | Clistoabdominalis | Clistoabdominalis digitatus |
| 72b0ed8d4cddafe447d01569531fb54e | 0 | 0 | 0 | Eukaryota | Metazoa | Arthropoda | Insecta | Diptera | Pipunculidae | Clistoabdominalis | Clistoabdominalis digitatus |
| 7f30dca592a2439a16d599fe4674368d | 0 | 0 | 0 | Eukaryota | Metazoa | Arthropoda | Insecta | Diptera | Pipunculidae | Clistoabdominalis | Clistoabdominalis digitatus |
| 8107afe43dd932d51973d670622fd93e | 0 | 0 | 0 | Eukaryota | Metazoa | Arthropoda | Insecta | Diptera | Pipunculidae | Clistoabdominalis | Clistoabdominalis digitatus |
| 8424cccd50aa8031a384026410d215bb | 0 | 0 | 0 | Eukaryota | Metazoa | Arthropoda | Insecta | Diptera | Pipunculidae | Clistoabdominalis | Clistoabdominalis digitatus |
| a68e343aadc595b54a5a22355b3f2aa5 | 0 | 0 | 0 | Eukaryota | Metazoa | Arthropoda | Insecta | Diptera | Pipunculidae | Clistoabdominalis | Clistoabdominalis digitatus |
| a936ab4feee4fb457db6f9be534c0ba7 | 0 | 0 | 0 | Eukaryota | Metazoa | Arthropoda | Insecta | Diptera | Pipunculidae | Clistoabdominalis | Clistoabdominalis digitatus |
| a97f306a169d825b19e9c8cf9bc211f6 | 0 | 0 | 0 | Eukaryota | Metazoa | Arthropoda | Insecta | Diptera | Pipunculidae | Clistoabdominalis | Clistoabdominalis digitatus |
| b8ee2964272696231e8d4fde685b41de | 0 | 0 | 0 | Eukaryota | Metazoa | Arthropoda | Insecta | Diptera | Pipunculidae | Clistoabdominalis | Clistoabdominalis digitatus |
| bc5413a67107ec57341162bd3d8472d9 | 0 | 0 | 0 | Eukaryota | Metazoa | Arthropoda | Insecta | Diptera | Pipunculidae | Clistoabdominalis | Clistoabdominalis digitatus |
| bf1ef7e9289e9e9ae2083a3d88b32e09 | 0 | 0 | 0 | Eukaryota | Metazoa | Arthropoda | Insecta | Diptera | Pipunculidae | Clistoabdominalis | Clistoabdominalis digitatus |
| c0a1eb4811f31c13a805e0005512573b | 0 | 0 | 0 | Eukaryota | Metazoa | Arthropoda | Insecta | Diptera | Pipunculidae | Clistoabdominalis | Clistoabdominalis digitatus |
| c3510580ca801f46738ee4961811bb60 | 0 | 0 | 0 | Eukaryota | Metazoa | Arthropoda | Insecta | Diptera | Pipunculidae | Clistoabdominalis | Clistoabdominalis digitatus |
| d605ec6128565a6c19f10cd7da545436 | 0 | 0 | 0 | Eukaryota | Metazoa | Arthropoda | Insecta | Diptera | Pipunculidae | Clistoabdominalis | Clistoabdominalis digitatus |
| e40117b39922f4d6c06d8c4d5d967ba4 | 0 | 0 | 0 | Eukaryota | Metazoa | Arthropoda | Insecta | Diptera | Pipunculidae | Clistoabdominalis | Clistoabdominalis digitatus |
| e72b8a0f9ca445360ed6aaaedc4dc0f4 | 0 | 0 | 0 | Eukaryota | Metazoa | Arthropoda | Insecta | Diptera | Pipunculidae | Clistoabdominalis | Clistoabdominalis digitatus |
| f33bf11cb4c508d93a10dd86b70b84c8 | 0 | 0 | 0 | Eukaryota | Metazoa | Echinodermata | Crinoidea |  |  |  | Crinoidea sp. NHM_008 |
| 309a7c9a434caa70385fe8a9915b5083 | 0 | 0 | 0 | Eukaryota | Metazoa | Arthropoda | Malacostraca | Isopoda | Cymothoidae | Cymothoa | Cymothoa truncata |
| ab5a874162c0f599a8ea89749b1cde6e | 0 | 0 | 0 | Eukaryota | Metazoa | Chordata | Mammalia | Chiroptera | Molossidae | Cynomops | Cynomops paranus |
| 49cf8aeacba37bb33e6ead1dc603d692 | 0 | 0 | 0 | Eukaryota | Metazoa | Chordata | Aves | Passeriformes | Certhiidae | Cyphorhinus | Cyphorhinus arada |
| 317cdf8dfa2a5d8ce8c44528a6afb706 | 0 | 0 | 0 | Eukaryota | Metazoa | Chordata | Actinopteri | Blenniiformes | Dactyloscopidae | Dactyloscopus | Dactyloscopus tridigitatus |
| 16bcba5a277b434374fe482f2c23a5d2 | 0 | 0 | 0 | Eukaryota | Metazoa | Chordata | Actinopteri |  | Pomacentridae | Dascyllus | Dascyllus aruanus |
| 929391012990605acb06b6a916f3ee06 | 0 | 0 | 0 | Eukaryota | Metazoa | Arthropoda | Insecta | Orthoptera | Acrididae | Diabolocatantops | Diabolocatantops sp. BOLD:AAW5355 |
| 89f033ee291579b1638addda5c72a270 | 0 | 0 | 0 | Eukaryota | Metazoa | Chordata | Actinopteri | Lophiiformes | Ogcocephalidae | Dibranchus | Dibranchus atlanticus |
| 77ff31f998df974c6997adbc92f5b357 | 169 | 98 | 0 | Eukaryota | Metazoa | Mollusca | Gastropoda | Architaenioglossa | Diplommatinidae | Diplommatina | Diplommatina plecta |
| 60ce93d178c12a614a597907c81a43ec | 0 | 0 | 0 | Eukaryota | Metazoa | Arthropoda | Insecta | Trichoptera | Dipseudopsidae | Dipseudopsis | Dipseudopsis africana |
| 0cdad98ee2d166a8efd12c5a4abc8d87 | 4 | 0 | 0 | Eukaryota | Metazoa | Arthropoda | Insecta | Psocoptera | Psyllipsocidae | Dorypteryx | Dorypteryx domestica |
| e03b0b8a709bb39c984e08961f5c9e5b | 0 | 0 | 0 | Eukaryota | Metazoa | Arthropoda | Insecta | Hymenoptera | Dryinidae |  | Dryinidae sp. BOLD:ACL6033 |
| 582ef505d1ae1c1fab5f7b4cf382c648 | 0 | 0 | 0 | Eukaryota | Metazoa | Platyhelminthes | Rhabditophora | Tricladida | Dugesiidae | Dugesia | Dugesia naiadis |
| b73cfe920f532e119c11da35f8862005 | 0 | 8 | 0 | Eukaryota | Metazoa | Arthropoda | Arachnida | Araneae | Dysderidae | Dysdera | Dysdera calderensis |
| e5512dae70ecbee0113c00d8eeb2a175 | 71 | 0 | 0 | Eukaryota | Metazoa | Annelida | Clitellata | Haplotaxida | Enchytraeidae |  | Enchytraeidae sp. En_442 |
| 8bddb9f4a531784b33b45a6108138968 | 0 | 0 | 0 | Eukaryota | Metazoa | Bryozoa | Gymnolaemata | Cheilostomatida | Romancheinidae | Escharella | Escharella immersa |
| fdd4dd3f60036cd206dd318efdd2aa14 | 14 | 36 | 0 | Eukaryota | Metazoa | Annelida | Polychaeta | Phyllodocida | Phyllodocidae | Eumida | Eumida kelaino |
| 044389ec59b8f3360ba227e1230a7d7a | 0 | 0 | 0 | Eukaryota | Metazoa | Arthropoda | Malacostraca | Isopoda | Cirolanidae | Excirolana | Excirolana braziliensis |
| f266f698c8850cf0698507a6e1b1cb38 | 83 | 0 | 0 | Eukaryota | Metazoa | Arthropoda | Malacostraca | Isopoda | Cirolanidae | Excirolana | Excirolana braziliensis |
| ad3b7cf5687545a3e090d9b4209bda37 | 0 | 0 | 0 | Eukaryota | Metazoa | Mollusca | Gastropoda |  |  |  | Gastropoda sp. IOP_0604 |
| be5f1c452cad8ee2569505d2cce8442b | 0 | 0 | 0 | Eukaryota | Metazoa | Arthropoda | Insecta | Phthiraptera | Trichodectidae | Geomydoecus | Geomydoecus perotensis |
| c18fea0380bed2d81f31205aabec4858 | 0 | 0 | 0 | Eukaryota | Metazoa | Arthropoda | Insecta | Phthiraptera | Trichodectidae | Geomydoecus | Geomydoecus perotensis |
| bcb2f5add3f33b5fdb968d5aab4190e0 | 0 | 0 | 0 | Eukaryota | Metazoa | Chordata | Aves | Strigiformes | Strigidae | Glaucidium | Glaucidium passerinum |
| 9d475f617b01c1789e123aef3ec47b39 | 0 | 0 | 0 | Eukaryota | Metazoa | Chordata | Aves | Strigiformes | Strigidae | Glaucidium | Glaucidium tephronotum |
| 9c8e2787e172fd0a9777b1dffeef3f21 | 0 | 0 | 0 | Eukaryota | Metazoa | Chordata | Aves | Passeriformes | Formicariidae | Grallaria | Grallaria guatimalensis |
| 37cc0ca7efd917e0c2c3e7db217b4c8d | 8 | 0 | 0 | Eukaryota | Metazoa | Platyhelminthes | Monogenea | Gyrodactylidea | Gyrodactylidae | Gyrodactylus | Gyrodactylus pannonicus |
| 2d230758d5bb1bba16bea21f706c6f9a | 0 | 0 | 2 | Eukaryota | Metazoa | Arthropoda | Insecta | Diptera | Muscidae | Hebecnema | Hebecnema sp. BOLD-2016 |
| 1ba81e000e8b38ea9981469cb56cf318 | 0 | 6 | 0 | Eukaryota | Metazoa | Arthropoda | Insecta | Hymenoptera | Braconidae | Heterospilus | Heterospilus sp. BOLD:AAI1758 |
| 607918e5b817cb68ec75ca5ea19d4478 | 0 | 0 | 0 | Eukaryota | Metazoa | Arthropoda | Insecta | Hymenoptera | Braconidae | Heterospilus | Heterospilus sp. BOLD:AAI1758 |
| df781ae77319a6028c6a640206fafacf | 0 | 0 | 0 | Eukaryota | Metazoa | Arthropoda | Insecta | Hymenoptera | Braconidae | Heterospilus | Heterospilus sp. BOLD:AAI1758 |
| 021d52677f7a94c81661b17993822bf2 | 8 | 10 | 0 | Eukaryota | Metazoa | Arthropoda | Insecta | Hemiptera | Membracidae | Hypsauchenia | Hypsauchenia sp. CPL-2004 |
| bfbe8bc2d864cf0517d2bcb9e43811a7 | 0 | 0 | 0 | Eukaryota | Metazoa | Arthropoda | Arachnida | Ixodida | Ixodidae | Ixodes | Ixodes scapularis |
| d8e267e1037c0635c45f43502f03e93f | 0 | 0 | 4 | Eukaryota | Metazoa | Arthropoda | Arachnida | Ixodida | Ixodidae | Ixodes | Ixodes scapularis |
| 6c5d58a2bced961168e804015e94471b | 0 | 0 | 0 | Eukaryota | Metazoa | Chordata | Actinopteri |  | Sciaenidae | Johnius | Johnius carouna |
| 0132104f19475a35ba6cc5d07398ae40 | 0 | 0 | 0 | Eukaryota | Metazoa | Arthropoda | Insecta | Hemiptera | Tropiduchidae | Kallitaxila | Kallitaxila sinica |
| c1e05e4bbe69b2ff2641abc536b1a204 | 0 | 0 | 0 | Eukaryota | Metazoa | Chordata | Actinopteri | Atheriniformes | Atherinopsidae | Labidesthes | Labidesthes sicculus |
| 1e55132eff8cc2084a780b839748b4a1 | 0 | 0 | 0 | Eukaryota | Metazoa | Arthropoda | Arachnida | Mesostigmata | Laelapidae |  | Laelapidae sp. BOLD:ACI9030 |
| 02d876aa7e08f972c661231541e72697 | 0 | 0 | 0 | Eukaryota | Metazoa | Arthropoda | Malacostraca | Isopoda | Ligiidae | Ligia | Ligia pallasii |
| 54c8e3d20650082b3209fbcec791e657 | 0 | 0 | 0 | Eukaryota | Metazoa | Arthropoda | Chilopoda | Lithobiomorpha | Lithobiidae | Lithobius | Lithobius sp. BOLD:AAH6437 |
| 9a10f332bd01fada543aa2436aa39deb | 0 | 0 | 0 | Eukaryota | Metazoa | Cnidaria | Scyphozoa | Rhizostomeae | Lobonematidae |  | Lobonematidae sp. 4 LGD-2017 |
| 105e92529e0694d7d0b17673db6d4680 | 0 | 0 | 0 | Eukaryota | Metazoa | Arthropoda | Arachnida | Araneae | Ctenidae | Macroctenus | Macroctenus cf. kingsleyi MRAC 238214 DNA N0 |
| 27aa6b4f92976a30b52c384ad38727e2 | 0 | 0 | 0 | Eukaryota | Metazoa | Arthropoda | Arachnida | Araneae | Ctenidae | Macroctenus | Macroctenus cf. kingsleyi MRAC 238214 DNA N0 |
| 45b75a8416c49d9dfbc892a54037512b | 0 | 0 | 0 | Eukaryota | Metazoa | Arthropoda | Arachnida | Araneae | Ctenidae | Macroctenus | Macroctenus cf. kingsleyi MRAC 238214 DNA N0 |
| 47cb763a41b81b531a1e78e1475ce4b3 | 0 | 0 | 0 | Eukaryota | Metazoa | Arthropoda | Arachnida | Araneae | Ctenidae | Macroctenus | Macroctenus cf. kingsleyi MRAC 238214 DNA N0 |
| eb52dc3498e6a5a191e02e3087a39f93 | 0 | 0 | 0 | Eukaryota | Metazoa | Arthropoda | Arachnida | Araneae | Ctenidae | Macroctenus | Macroctenus cf. kingsleyi MRAC 238214 DNA N0 |
| f94bd6f98867d7489a2732e066b6eb09 | 3 | 0 | 0 | Eukaryota | Metazoa | Arthropoda | Arachnida | Araneae | Ctenidae | Macroctenus | Macroctenus cf. kingsleyi MRAC 238214 DNA N0 |
| 51ddfb64ae380382f27ab7e77294120a | 0 | 0 | 0 | Eukaryota | Metazoa | Arthropoda | Malacostraca | Amphipoda | Stenothoidae | Metopa | Metopa boeckii |
| 49d9a2992ea8f128efc31f081f04fb73 | 0 | 0 | 0 | Eukaryota | Metazoa | Arthropoda | Hexanauplia | Calanoida | Metridinidae | Metridia | Metridia gerlachei |
| bcce511319c7fa5700b921279c7759c2 | 0 | 0 | 0 | Eukaryota | Metazoa | Arthropoda | Hexanauplia | Calanoida | Metridinidae | Metridia | Metridia gerlachei |
| 48e6e5fb0486957c2a247fed88ebe152 | 33 | 13 | 0 | Eukaryota | Metazoa | Mollusca | Bivalvia | Pectinoida | Pectinidae | Mimachlamys | Mimachlamys nobilis |
| 69d698a262033d8cd4bd0b538eb17913 | 0 | 0 | 0 | Eukaryota | Metazoa | Ctenophora | Tentaculata | Lobata | Bolinopsidae | Mnemiopsis | Mnemiopsis leidyi |
| 7ce2a1de0479b954fa615c1c166a9ee9 | 0 | 80 | 0 | Eukaryota | Metazoa | Chordata | Mammalia | Rodentia | Muridae | Mus | Mus musculus |
| ad924262a050e757f4b5ecf4740eb42b | 0 | 4 | 0 | Eukaryota | Metazoa | Mollusca | Gastropoda | Neogastropoda | Terebridae |  | Myurella sp. NP-2009 |
| 038162c435c90d36837c481ae1b8ced1 | 0 | 15 | 0 | Eukaryota | Metazoa | Arthropoda | Insecta | Hemiptera | Aphididae | Neoamphorophora | Neoamphorophora kalmiae |
| c2cac860828c95a83e718fb6c3083a48 | 0 | 0 | 0 | Eukaryota | Metazoa | Chordata | Actinopteri | Cypriniformes | Cyprinidae | Notropis | Notropis baileyi |
| 982b2323f9ce221cdd9fe306ca94221d | 0 | 0 | 0 | Eukaryota | Metazoa | Echinodermata | Asteroidea | Valvatida | Odontasteridae | Odontaster | Odontaster validus |
| 623b4c18087252161e87aa6b3b231266 | 0 | 0 | 0 | Eukaryota | Metazoa | Chordata | Aves | Columbiformes | Columbidae | Oena | Oena capensis |
| 3f059d653b1dc71a0fea8c76a069dc1a | 0 | 0 | 0 | Eukaryota | Metazoa | Arthropoda | Collembola | Poduromorpha | Onychiuridae |  | Onychiuridae sp. DPCOL101273 |
| 4226040095340332184ab37b21930bb8 | 0 | 0 | 0 | Eukaryota | Metazoa | Arthropoda | Collembola | Poduromorpha | Onychiuridae |  | Onychiuridae sp. DPCOL101273 |
| 8286e0f2a630c5064918b2e5d340c13a | 0 | 0 | 0 | Eukaryota | Metazoa | Arthropoda | Collembola | Poduromorpha | Onychiuridae |  | Onychiuridae sp. DPCOL101273 |
| 87ca2999061d05cc34833a57fdfa948f | 0 | 0 | 0 | Eukaryota | Metazoa | Arthropoda | Collembola | Poduromorpha | Onychiuridae |  | Onychiuridae sp. DPCOL101273 |
| 27c47cf0f6454548b8b61ca71b1fda10 | 0 | 0 | 0 | Eukaryota | Metazoa | Echinodermata | Ophiuroidea | Ophiurida | Ophiuridae | Ophionotus | Ophionotus victoriae |
| 34349a9bd68aad880d06eadd0580adb3 | 0 | 0 | 0 | Eukaryota | Metazoa | Echinodermata | Ophiuroidea | Ophiurida | Ophiuridae | Ophionotus | Ophionotus victoriae |
| aa77bf1c1d8c1c4c54036e2d4a22f17e | 46 | 122 | 77 | Eukaryota | Metazoa | Echinodermata | Ophiuroidea | Ophiurida | Ophiuridae | Ophionotus | Ophionotus victoriae |
| c5ede58e1ba520be3c226d9af3fe5ae9 | 103 | 182 | 137 | Eukaryota | Metazoa | Echinodermata | Ophiuroidea | Ophiurida | Ophiuridae | Ophionotus | Ophionotus victoriae |
| e413ba4affa1724c22adfa4a29b6456b | 0 | 0 | 0 | Eukaryota | Metazoa | Echinodermata | Ophiuroidea | Ophiurida | Ophiuridae | Ophionotus | Ophionotus victoriae |
| 08776efb618bab7db91cbbc7bdb8ad60 | 200 | 250381 | 133 | Eukaryota | Metazoa | Annelida | Polychaeta | Eunicida | Dorvilleidae | Ophryotrocha | Ophryotrocha clava |
| 1ab21ffedcba2eaf938f38403575331e | 0 | 751 | 0 | Eukaryota | Metazoa | Annelida | Polychaeta | Eunicida | Dorvilleidae | Ophryotrocha | Ophryotrocha clava |
| 1fd6c0b4544074da97257b8ae088c534 | 0 | 386 | 0 | Eukaryota | Metazoa | Annelida | Polychaeta | Eunicida | Dorvilleidae | Ophryotrocha | Ophryotrocha clava |
| 4db6e15518fa36d0f21c5c1b18ab2426 | 0 | 9164 | 0 | Eukaryota | Metazoa | Annelida | Polychaeta | Eunicida | Dorvilleidae | Ophryotrocha | Ophryotrocha clava |
| 9b610c0817198248eabdd1a50e3c45eb | 0 | 4 | 0 | Eukaryota | Metazoa | Annelida | Polychaeta | Sabellida | Siboglinidae | Osedax | Osedax sp. 'green-palp' |
| 024f89fd4819918fb10b516fd09de684 | 0 | 0 | 68 | Eukaryota | Metazoa | Arthropoda | Hexanauplia | Calanoida | Acartiidae | Paralabidocera | Paralabidocera grandispina |
| 2978acf0cabd5e5e96373e7b5b1c750d | 0 | 0 | 27 | Eukaryota | Metazoa | Arthropoda | Hexanauplia | Calanoida | Acartiidae | Paralabidocera | Paralabidocera grandispina |
| 99d529220add5867a5b7b5517e24ee0a | 0 | 0 | 58 | Eukaryota | Metazoa | Arthropoda | Hexanauplia | Calanoida | Acartiidae | Paralabidocera | Paralabidocera grandispina |
| b95764f468758182a51b0204150408fd | 0 | 0 | 44 | Eukaryota | Metazoa | Arthropoda | Hexanauplia | Calanoida | Acartiidae | Paralabidocera | Paralabidocera grandispina |
| bbbc12cd212fb39788b8c338ca1df94e | 104 | 0 | 0 | Eukaryota | Metazoa | Arthropoda | Hexanauplia | Calanoida | Acartiidae | Paralabidocera | Paralabidocera grandispina |
| ccd7960a78c504410cd0e608fe25b20a | 0 | 0 | 0 | Eukaryota | Metazoa | Arthropoda | Hexanauplia | Calanoida | Acartiidae | Paralabidocera | Paralabidocera grandispina |
| d69d7d4316c07d66e97321f061f55e96 | 0 | 0 | 0 | Eukaryota | Metazoa | Arthropoda | Hexanauplia | Calanoida | Acartiidae | Paralabidocera | Paralabidocera grandispina |
| e12e67cc1c4bf4aeb1762da979d5a76c | 0 | 0 | 0 | Eukaryota | Metazoa | Arthropoda | Hexanauplia | Calanoida | Acartiidae | Paralabidocera | Paralabidocera grandispina |
| c47af405209be7d7c485ec43577822eb | 1777 | 4656 | 123 | Eukaryota | Metazoa | Arthropoda | Insecta | Lepidoptera | Geometridae | Patalene | Patalene asychisaria |
| 59474d690d66cc3b4a87bff9f4691d6b | 0 | 0 | 0 | Eukaryota | Metazoa | Chordata | Actinopteri | Pempheriformes | Pempheridae | Pempheris | Pempheris schomburgkii |
| aeec72f6a05529ef56cc2487a59525fb | 0 | 0 | 0 | Eukaryota | Metazoa | Rotifera | Bdelloidea | Philodinida | Philodinidae | Philodina | Philodina megalotrocha |
| 7570ca83d62db5c984a1bba1733e134e | 0 | 0 | 0 | Eukaryota | Metazoa | Arthropoda | Arachnida | Araneae | Pimoidae | Pimoa | Pimoa sp. SP74 |
| 88abc7f2d23fbbe769be3b63a6ed9be4 | 0 | 0 | 0 | Eukaryota | Metazoa | Mollusca | Gastropoda | Architaenioglossa | Ampullariidae | Pomacea | Pomacea flagellata |
| ae36f7dd0ee6e1b35c86180b7ca76ceb | 0 | 0 | 0 | Eukaryota | Metazoa | Mollusca | Gastropoda | Acochlidiacea | Microhedylidae | Pontohedyle | Pontohedyle milaschewitchii |
| f69a438cf6cd050902020ec4c4abd80e | 0 | 0 | 0 | Eukaryota | Metazoa | Arthropoda | Malacostraca | Decapoda | Portunidae | Portunus | Portunus segnis |
| 76e992a5e227ddcbaa7cc2f5934aa31e | 23 | 0 | 0 | Eukaryota | Metazoa | Arthropoda | Malacostraca | Isopoda | Asellidae | Proasellus | Proasellus escolai |
| b6abe091223a3293f5c04959f1b3353b | 0 | 0 | 0 | Eukaryota | Metazoa | Arthropoda | Insecta | Trichoptera | Glossosomatidae | Protoptila | Protoptila voluta |
| d1609c1f386be7fc5fb6a5d6eb3334da | 0 | 0 | 0 | Eukaryota | Metazoa | Arthropoda | Arachnida | Araneae | Pholcidae | Psilochorus | Psilochorus hesperus |
| c744b206edbfcec355a78fbeb6f97339 | 0 | 5 | 0 | Eukaryota | Metazoa | Echinodermata | Holothuroidea | Dendrochirotida | Psolidae | Psolus | Psolus charcoti |
| 34bd79265ba58b213aa4ef9c483b867a | 0 | 0 | 0 | Eukaryota | Metazoa | Arthropoda | Insecta | Hymenoptera | Pteromalidae |  | Pteromalidae sp. BOLD-2016 |
| 8a52dd2dd4e431f95f536ee257fd9d8c | 0 | 0 | 0 | Eukaryota | Metazoa | Mollusca | Gastropoda | Neogastropoda | Costellariidae | Pusia | Pusia ebenus |
| a6e019c4eb9033dc67ddd5297790dadc | 0 | 0 | 0 | Eukaryota | Metazoa | Mollusca | Gastropoda | Neogastropoda | Costellariidae | Pusia | Pusia ebenus |
| c5f4e8a7aa7fc01e9e568d0ee82adab6 | 0 | 0 | 0 | Eukaryota | Metazoa | Kinorhyncha |  | Homalorhagida | Pycnophyidae | Pycnophyes | Pycnophyes flaveolatus |
| 778f60d6e06e2fe92127ca766ba82805 | 0 | 0 | 0 | Eukaryota | Metazoa | Arthropoda | Insecta | Diptera | Sciaridae |  | Sciaridae sp. BOLD:ACL4012 |
| ec6bf28b07a99c9518ec958a5a422135 | 0 | 9 | 0 | Eukaryota | Metazoa | Cnidaria | Hydrozoa | Narcomedusae | Cuninidae | Solmissus | Solmissus marshalli |
| 536a2504453b318a8b3437a969ab3c4e | 0 | 0 | 0 | Eukaryota | Metazoa | Arthropoda | Insecta | Hymenoptera | Braconidae | Spathius | Spathius aff. caudatus 1 AZR-2017 |
| da86e1d9f47ecbee986da0c249c045c2 | 0 | 8 | 0 | Eukaryota | Metazoa | Mollusca | Bivalvia | Arcoida | Arcidae | Tegillarca | Tegillarca nodifera |
| 022ce19250d18ecb26b61c640eda0273 | 0 | 12 | 0 | Eukaryota | Metazoa | Platyhelminthes | Rhabditophora | Rhabdocoela | Temnocephalidae | Temnosewellia | Temnosewellia albata |
| b68d4e4a5b71c2bed0cc29befd999b7c | 0 | 0 | 0 | Eukaryota | Metazoa | Platyhelminthes | Rhabditophora | Rhabdocoela | Temnocephalidae | Temnosewellia | Temnosewellia albata |
| cd0aaf55bf8d95b7d814772728cbcdf9 | 19 | 0 | 7 | Eukaryota | Metazoa | Platyhelminthes | Rhabditophora | Rhabdocoela | Temnocephalidae | Temnosewellia | Temnosewellia albata |
| e05a88e84160abd2e9b6a03c19fe24bb | 0 | 0 | 0 | Eukaryota | Metazoa | Arthropoda | Arachnida | Sarcoptiformes | Terpnacaridae |  | Terpnacaridae sp. BOLD:AAU6267 |
| 2db18cb4bbf8818a455e9fd61873538e | 0 | 0 | 0 | Eukaryota | Metazoa | Chordata | Actinopteri | Labriformes | Labridae | Thalassoma | Thalassoma quinquevittatum |
| ca0fe9710f24c79507aaec76e93f5f37 | 0 | 0 | 0 | Eukaryota | Metazoa | Annelida | Polychaeta | Terebellida | Terebellidae | Thelepus | Thelepus plagiostoma |
| a5dc4cebcfb9a3ff0aaf1a5d96223281 | 0 | 0 | 0 | Eukaryota | Metazoa | Arthropoda | Malacostraca | Decapoda | Hippolytidae | Tozeuma | Tozeuma carolinense |
| d19488b399402afa3f411d812814cb04 | 0 | 0 | 0 | Eukaryota | Metazoa | Arthropoda | Insecta | Coleoptera | Curculionidae | Trigonopterus | Trigonopterus sp. 621 MB-2015 |
| 0e83ce82bdc4c05fdceb584b8c1a4748 | 0 | 0 | 0 | Eukaryota | Metazoa | Mollusca | Bivalvia | Unionoida | Unionidae | Unio | Unio durieui |
| 831fd40b5e226f9ba3e551d843a083fb | 11 | 0 | 0 | Eukaryota | Metazoa | Chordata |  | Squamata | Phrynosomatidae | Uta | Uta stansburiana |
| 03d1d0e4744992cea69c783c23492768 | 0 | 0 | 0 | Eukaryota | Metazoa | Arthropoda | Arachnida | Araneae | Thomisidae | Xysticus | Xysticus bimaculatus |
| 44331df7f8fd1bd2d9de398b6671a6f5 | 0 | 0 | 0 | Eukaryota | Metazoa | Arthropoda | Arachnida | Araneae | Thomisidae | Xysticus | Xysticus durus |
| 51518acfa94d710f3f207cc63bbdd3b3 | 1661 | 62103 | 82294 | Eukaryota | Metazoa | Mollusca | Bivalvia | Nuculanoida | Yoldiidae | Yoldia | Yoldia eightsii |
| 952a48a38980fe174027fbf83a726534 | 0 | 0 | 0 | Eukaryota | Metazoa | Mollusca | Bivalvia | Nuculanoida | Yoldiidae | Yoldia | Yoldia eightsii |
| a247f6317f88869f57f03b49e184019d | 0 | 28 | 0 | Eukaryota | Metazoa | Mollusca | Bivalvia | Nuculanoida | Yoldiidae | Yoldia | Yoldia eightsii |
| e046b3168cca4162df81a8644b558c40 | 0 | 33 | 0 | Eukaryota | Metazoa | Mollusca | Bivalvia | Nuculanoida | Yoldiidae | Yoldia | Yoldia eightsii |
| 03e81e297b6623f4eb0a902ad85dc1e5 | 0 | 0 | 0 | Eukaryota | Metazoa | Annelida | Polychaeta | Phyllodocida | Nephtyidae |  |  |
| 1fcb35f2427dfc4bed9fc254e46bb1ae | 0 | 0 | 9 | Eukaryota | Metazoa | Annelida | Polychaeta | Phyllodocida | Nephtyidae |  |  |
| 39e26f74d65694291cf5576cf2ba2a77 | 132 | 0 | 0 | Eukaryota | Metazoa | Annelida | Polychaeta | Phyllodocida | Nephtyidae |  |  |
| 3d6829010866a65b9a9b23ea063e1bee | 0 | 0 | 0 | Eukaryota | Metazoa | Annelida | Polychaeta | Phyllodocida | Syllidae |  |  |
| 6eaab046f3e2abf830bdc00e5b3754b9 | 5554 | 131 | 636 | Eukaryota | Metazoa | Annelida | Polychaeta | Phyllodocida | Nephtyidae |  |  |
| 8b921e48355fa2d3d8865d1e9314fa29 | 111 | 0 | 0 | Eukaryota | Metazoa | Annelida | Polychaeta | Phyllodocida | Nephtyidae |  |  |
| b218ade6615cf431f64549fc2381b750 | 0 | 0 | 0 | Eukaryota | Metazoa | Annelida | Polychaeta | Phyllodocida | Nephtyidae |  |  |
| b62ed6887473a2c005f7755af1e557d1 | 400 | 0 | 0 | Eukaryota | Metazoa | Annelida | Polychaeta | Phyllodocida | Nephtyidae |  |  |
| bd51ab145696c70bea3cd3f51d0ff77e | 0 | 0 | 0 | Eukaryota | Metazoa | Annelida | Polychaeta | Phyllodocida | Nephtyidae |  |  |
| bfe73aefcfc17b1366fd253f4dff1b5f | 52 | 0 | 0 | Eukaryota | Metazoa | Annelida | Polychaeta | Phyllodocida | Syllidae |  |  |
| c026982b2e750c8180dd9fbf53a2ad80 | 7871 | 275 | 468 | Eukaryota | Metazoa | Annelida | Polychaeta | Phyllodocida | Nephtyidae |  |  |
| c27ec963a5bddf31574c53a4b762518b | 20252 | 1473 | 3988 | Eukaryota | Metazoa | Annelida | Polychaeta | Phyllodocida | Nephtyidae |  |  |
| ddb8a851dc2fb4d8b1cd2a786dcbcd63 | 0 | 0 | 0 | Eukaryota | Metazoa | Annelida | Polychaeta | Phyllodocida | Nephtyidae |  |  |
| e93b2240ca1a26ddd64935c2a5b6a500 | 0 | 0 | 0 | Eukaryota | Metazoa | Annelida | Polychaeta | Phyllodocida | Nephtyidae |  |  |
| eba1485e59af541ab50db5f2fc056bc1 | 0 | 67 | 0 | Eukaryota | Metazoa | Annelida | Polychaeta | Phyllodocida | Nephtyidae |  |  |
| f498bd369169dd037cc21ab6d2e4ab37 | 0 | 0 | 0 | Eukaryota | Metazoa | Annelida | Polychaeta | Phyllodocida | Nephtyidae |  |  |
| 09b18a9c4fcb593dba0f5d9211c9401f | 0 | 0 | 30 | Eukaryota | Metazoa | Arthropoda | Arachnida | Araneae | Idiopidae | Idiosoma |  |
| 0a328331716814a12e7cc8a01ab66f0b | 0 | 0 | 0 | Eukaryota | Metazoa | Arthropoda | Insecta | Hymenoptera | Ichneumonidae | Sussaba |  |
| 18a2e110004e455b097f83f0c05922c6 | 0 | 0 | 0 | Eukaryota | Metazoa | Arthropoda |  |  |  |  |  |
| 1d08860f1f02226df177b2e0a8b08994 | 334 | 0 | 0 | Eukaryota | Metazoa | Arthropoda | Insecta | Hymenoptera |  |  |  |
| 219a853a0a730d1f261dd01ef137372f | 0 | 5 | 0 | Eukaryota | Metazoa | Arthropoda | Insecta | Hymenoptera |  |  |  |
| 259e93f0bb65d07780a9b5ce5e757bec | 0 | 0 | 0 | Eukaryota | Metazoa | Arthropoda | Insecta | Hymenoptera |  |  |  |
| 2b91e6d72b48a449e4a36a1eb31fd98f | 0 | 0 | 0 | Eukaryota | Metazoa | Arthropoda | Insecta | Odonata | Gomphidae |  |  |
| 4ceafea83b9a28cfb4a0f341c9e25204 | 6 | 0 | 0 | Eukaryota | Metazoa | Arthropoda | Insecta |  |  |  |  |
| 7f8ade63fe041f2b9340d3bd51201fd8 | 52 | 93 | 16 | Eukaryota | Metazoa | Arthropoda | Arachnida | Sarcoptiformes | Eremellidae |  |  |
| 85bdd26a0ff9657d9d3d0f6fee445f9f | 0 | 0 | 0 | Eukaryota | Metazoa | Arthropoda | Insecta |  |  |  |  |
| a21b497bf2bb562fd354e41a49657fb2 | 0 | 0 | 0 | Eukaryota | Metazoa | Arthropoda | Insecta | Hymenoptera | Ichneumonidae | Sussaba |  |
| c2c3f6cfb7fa7b31e94dc12a632819a0 | 0 | 0 | 0 | Eukaryota | Metazoa | Arthropoda | Insecta | Plecoptera | Perlodidae | Perlodes |  |
| c5ab042adabf53629c87aa52668f9d3f | 0 | 0 | 0 | Eukaryota | Metazoa | Arthropoda | Insecta |  |  |  |  |
| db2ae51f2667f1784825677fde8fdff9 | 0 | 0 | 0 | Eukaryota | Metazoa | Arthropoda |  |  |  |  |  |
| ddc4af043f5cf8d8f1df36eade48f712 | 0 | 0 | 0 | Eukaryota | Metazoa | Arthropoda | Arachnida | Mesostigmata | Phytoseiidae |  |  |
| e9a28771acd1eadcbc6e3cbde61b6d97 | 0 | 0 | 33 | Eukaryota | Metazoa | Arthropoda | Arachnida | Araneae | Ctenidae | Macroctenus |  |
| fa059b9b01c9a35a7810e8938588daca | 39 | 0 | 0 | Eukaryota | Metazoa | Arthropoda | Insecta | Hymenoptera | Ichneumonidae | Sussaba |  |
| fe2a798e52b52deb5f4945df43e2da07 | 53 | 96 | 0 | Eukaryota | Metazoa | Arthropoda | Arachnida | Araneae | Idiopidae | Idiosoma |  |
| 46e05651925bb77d8a5dc5398736e827 | 0 | 0 | 16 | Eukaryota | Metazoa | Bryozoa | Gymnolaemata | Cheilostomatida | Watersiporidae | Watersipora |  |
| 0570abfcfec201193b5de68031237381 | 238 | 178 | 32 | Eukaryota | Metazoa | Chordata |  |  |  |  |  |
| 08fb7435697fe5871371c4d08735a66f | 0 | 0 | 0 | Eukaryota | Metazoa | Chordata |  | Squamata |  |  |  |
| 3a17a2da986ef5f9fc4f284ccf62cf0f | 0 | 0 | 0 | Eukaryota | Metazoa | Chordata |  | Squamata |  |  |  |
| 3af4ceac3db9571c00383925b63140a7 | 0 | 0 | 0 | Eukaryota | Metazoa | Chordata |  | Squamata |  |  |  |
| 41d7aed584a3f627fbaa45301ac5f177 | 0 | 0 | 0 | Eukaryota | Metazoa | Chordata |  | Squamata |  |  |  |
| 4717177bb3a3232378589d5091aefc4d | 0 | 0 | 0 | Eukaryota | Metazoa | Chordata |  | Squamata |  |  |  |
| 57d22f660bfa49aed96abf13802d0113 | 0 | 0 | 0 | Eukaryota | Metazoa | Chordata |  |  |  |  |  |
| 5dcb313708db18bd631c29e23e586fe8 | 0 | 0 | 0 | Eukaryota | Metazoa | Chordata |  |  |  |  |  |
| 5fae74a14078ba92dbaaeaaddec8b856 | 0 | 0 | 0 | Eukaryota | Metazoa | Chordata |  | Squamata | Sphaerodactylidae | Teratoscincus |  |
| 70eead67d43adb04b6ab09ea17eaefa7 | 0 | 8 | 0 | Eukaryota | Metazoa | Chordata |  | Squamata |  |  |  |
| 7139578b9115bb49978ff089d92945b5 | 0 | 0 | 0 | Eukaryota | Metazoa | Chordata |  | Squamata |  |  |  |
| bf63b34a40703090e7ec34b977fb872c | 0 | 0 | 0 | Eukaryota | Metazoa | Chordata |  | Squamata |  |  |  |
| c50b8986853d82d013a754e78626de99 | 0 | 0 | 0 | Eukaryota | Metazoa | Chordata |  | Squamata |  |  |  |
| c601da78db9491eb1805623d4f5de22c | 0 | 0 | 0 | Eukaryota | Metazoa | Chordata | Actinopteri |  |  |  |  |
| d328665d161445eed285310b40120890 | 84 | 0 | 0 | Eukaryota | Metazoa | Chordata |  |  |  |  |  |
| d55840af22c23bb82eec5190a7c9935d | 0 | 0 | 0 | Eukaryota | Metazoa | Chordata |  | Squamata |  |  |  |
| e2bb1c3a78736e3966829a19ac8a6126 | 0 | 0 | 0 | Eukaryota | Metazoa | Chordata |  | Squamata |  |  |  |
| e9f6f145c3e8ae3bc51b88dfdf411dbd | 0 | 10 | 0 | Eukaryota | Metazoa | Chordata |  | Squamata | Colubridae |  |  |
| d7cb8a3f34136e0b30cf1b2d30fabe8d | 0 | 0 | 0 | Eukaryota | Metazoa | Cnidaria | Hydrozoa | Leptothecata |  |  |  |
| 8abe7aecc27ad932bb326e57320824f3 | 19 | 0 | 0 | Eukaryota | Metazoa | Echinodermata | Asteroidea | Valvatida | Oreasteridae |  |  |
| f9276215a1b42c931340b956ca4757b3 | 0 | 0 | 0 | Eukaryota | Metazoa | Rotifera | Bdelloidea | Philodinida | Philodinidae | Macrotrachela |  |
| 422fa6d3e6e5854405d3b34c711e6782 | 0 | 0 | 0 | Eukaryota | Metazoa |  |  |  |  |  |  |
| e3c8da75b02e2c934f9bc68fdb324aa5 | 0 | 0 | 0 | Eukaryota | Metazoa |  |  |  |  |  |  |
| bf101dd52daf29ce3bc230671ae9f3b2 | 0 | 0 | 0 | Eukaryota | Metazoa |  |  |  |  |  |  |
| e43dbc9268ae5ea4f77eb288a30327c6 | 0 | 0 | 0 | Eukaryota | Metazoa |  |  |  |  |  |  |
| b4e71ecd9e13a1ad8ac24a07bd359be8 | 0 | 0 | 0 | Eukaryota | Metazoa |  |  |  |  |  |  |

| #ASV ID | SC1 | SC2 | SC3 | Superkingdom | Kingdom | Phylum | Class | Order | Family | Genus | Species |
| --- | --- | --- | --- | --- | --- | --- | --- | --- | --- | --- | --- |
| f4940b9aa86f03f3f222b68e80f5abb8 | 25 | 0 | 0 | Eukaryota | Metazoa | Arthropoda | Insecta | Coleoptera | Chrysomelidae | Agelastica | Agelastica coerulea |
| a8fbcdd964d02e437d7c349c33ef46e4 | 0 | 0 | 251 | Eukaryota | Metazoa | Annelida | Polychaeta | Phyllodocida | Nephtyidae | Aglaophamus | Aglaophamus dibranchis |
| 25ca4a2cc56daffb4f7358070876a7f7 | 0 | 0 | 63 | Eukaryota | Metazoa | Annelida | Polychaeta | Phyllodocida | Nephtyidae | Aglaophamus | Aglaophamus trissophyllus |
| 566efce7f80634ef733a156d7897aa48 | 0 | 3695 | 0 | Eukaryota | Metazoa | Annelida | Polychaeta | Phyllodocida | Nephtyidae | Aglaophamus | Aglaophamus trissophyllus |
| 6fe8a6e5e146131488f1f71c009bd93b | 0 | 0 | 0 | Eukaryota | Metazoa | Annelida | Polychaeta | Phyllodocida | Nephtyidae | Aglaophamus | Aglaophamus trissophyllus |
| 8b60eaaa89e01572fbb88a5c810d4725 | 0 | 0 | 0 | Eukaryota | Metazoa | Annelida | Polychaeta | Phyllodocida | Nephtyidae | Aglaophamus | Aglaophamus trissophyllus |
| 54365498f436f498c61b93f369620772 | 0 | 0 | 15 | Eukaryota | Metazoa | Chordata | Amphibia | Anura | Dendrobatidae | Allobates | Allobates femoralis |
| 090c761191703bb1fbe9aedbaa249ebf | 17 | 0 | 9 | Eukaryota | Metazoa | Annelida | Clitellata | Haplotaxida | Lumbricidae | Allolobophora | Allolobophora chlorotica |
| 1a8056778c5201f4a87e8174689b1681 | 30 | 0 | 8 | Eukaryota | Metazoa | Annelida | Clitellata | Haplotaxida | Lumbricidae | Allolobophora | Allolobophora chlorotica |
| a3a92718e2c2c66253f67a931d71b459 | 0 | 20 | 0 | Eukaryota | Metazoa | Annelida | Clitellata | Haplotaxida | Lumbricidae | Allolobophora | Allolobophora chlorotica |
| 059a2b2e2ad5353094280cc6145d5743 | 0 | 40 | 0 | Eukaryota | Metazoa | Arthropoda | Insecta | Psocoptera | Psocidae | Amphigerontia | Amphigerontia contaminata |
| e69436c3a62912d6f93aa160bbbf643f | 5 | 0 | 0 | Eukaryota | Metazoa | Arthropoda | Insecta | Psocoptera | Psocidae | Amphigerontia | Amphigerontia contaminata |
| 649429954022cf584069b329535be8a8 | 5 | 0 | 0 | Eukaryota | Metazoa | Arthropoda | Insecta | Hymenoptera | Andrenidae | Andrena | Andrena hirticincta |
| b9c18ede9eddb649dbc5bf6c987819bd | 0 | 0 | 0 | Eukaryota | Metazoa | Bryozoa | Gymnolaemata | Cheilostomatida | Hippothoidae | Antarctothoa | Antarctothoa sp. 'polystachya' |
| 8405259960ffe8c1f96ffb7e3b266b7f | 5 | 0 | 0 | Eukaryota | Metazoa | Arthropoda | Insecta | Coleoptera | Scarabaeidae |  | Aphodiinae sp. BOLD:AAN5996 |
| 9c89f86603d5009aaad1ba263a1f2432 | 39 | 0 | 0 | Eukaryota | Metazoa | Arthropoda | Branchiopoda | Anostraca | Artemiidae | Artemia | Artemia frameshifta |
| c15aa075c6540ccafed8d96fffbac14d | 0 | 18 | 9 | Eukaryota | Metazoa | Arthropoda | Branchiopoda | Anostraca | Artemiidae | Artemia | Artemia frameshifta |
| e8e2d1c0958ffd1fe66b0a025e2b5645 | 0 | 0 | 0 | Eukaryota | Metazoa | Arthropoda | Branchiopoda | Anostraca | Artemiidae | Artemia | Artemia frameshifta |
| 8b788ddb9e2a3756faa6d951778cd55d | 0 | 447 | 397 | Eukaryota | Metazoa | Arthropoda | Insecta | Hymenoptera | Bethylidae | Austranesia | Austranesia sp. 17 IA-2018 |
| e7ebc5995c645793172dc81483dd0254 | 0 | 0 | 0 | Eukaryota | Metazoa | Arthropoda | Insecta | Coleoptera | Cerambycidae | Batocera | Batocera lineolata |
| a22ba1170c0e73063b45ea6c4a6f1880 | 11 | 10 | 0 | Eukaryota | Metazoa | Rotifera | Bdelloidea | o__ |  |  | Bdelloidea sp. nd |
| 05ef7552829e187ef8a04e28d2906c59 | 0 | 8 | 0 | Eukaryota | Metazoa | Mollusca | Bivalvia | Mytiloida | Mytilidae | Brachidontes | Brachidontes rodriguezii |
| 13f0ac391ccb0ff198728f4a6bbb1080 | 0 | 17 | 0 | Eukaryota | Metazoa | Mollusca | Bivalvia | Mytiloida | Mytilidae | Brachidontes | Brachidontes rodriguezii |
| 2fd28833b2028682aaf194384392aba9 | 0 | 8 | 0 | Eukaryota | Metazoa | Mollusca | Bivalvia | Mytiloida | Mytilidae | Brachidontes | Brachidontes rodriguezii |
| 30177e2d1ceea56c4cf6bfd00959febe | 0 | 9 | 0 | Eukaryota | Metazoa | Mollusca | Bivalvia | Mytiloida | Mytilidae | Brachidontes | Brachidontes rodriguezii |
| 46c8c5c9a419c628ad4d247b685f0180 | 0 | 6 | 0 | Eukaryota | Metazoa | Mollusca | Bivalvia | Mytiloida | Mytilidae | Brachidontes | Brachidontes rodriguezii |
| 4dc1520eeaed5ecb72f5b20bb3eaf9d4 | 0 | 19 | 0 | Eukaryota | Metazoa | Mollusca | Bivalvia | Mytiloida | Mytilidae | Brachidontes | Brachidontes rodriguezii |
| 74f4e1f5322b79500ff00a8b0fe3d240 | 0 | 14 | 0 | Eukaryota | Metazoa | Mollusca | Bivalvia | Mytiloida | Mytilidae | Brachidontes | Brachidontes rodriguezii |
| cee0814f80f9b069a35b42f0aa88de85 | 0 | 6 | 0 | Eukaryota | Metazoa | Mollusca | Bivalvia | Mytiloida | Mytilidae | Brachidontes | Brachidontes rodriguezii |
| e21b7ecf303ffeed0a482ef14fabae44 | 0 | 59 | 0 | Eukaryota | Metazoa | Mollusca | Bivalvia | Mytiloida | Mytilidae | Brachidontes | Brachidontes rodriguezii |
| f6059ad0632b151a911010c58d1a9c35 | 0 | 8 | 0 | Eukaryota | Metazoa | Mollusca | Bivalvia | Mytiloida | Mytilidae | Brachidontes | Brachidontes rodriguezii |
| dc466c8a8957cbe196b0a667bef0519d | 37 | 0 | 40 | Eukaryota | Metazoa | Arthropoda | Arachnida | Sarcoptiformes | Brachychthoniidae |  | Brachychthoniidae sp. BOLD:ABV0368 |
| 1aa2a7e7c9a95668503fc1dda7dfa56f | 4 | 0 | 0 | Eukaryota | Metazoa | Arthropoda | Insecta | Coleoptera | Staphylinidae | Cafius | Cafius australis |
| f81c6945ae7145b45090e0ed4fa32924 | 0 | 0 | 0 | Eukaryota | Metazoa | Arthropoda | Insecta | Coleoptera | Staphylinidae | Cafius | Cafius australis |
| a6382624a173c3484f3b498edfd2d4d3 | 0 | 5 | 0 | Eukaryota | Metazoa | Arthropoda | Insecta | Hymenoptera | Formicidae | Camponotus | Camponotus sp. MG053 |
| aefb5d3e7e9f41df356f9eb8ec52c7c4 | 17 | 0 | 0 | Eukaryota | Metazoa | Arthropoda | Insecta | Diptera | Cecidomyiidae |  | Cecidomyiidae sp. BIOUG20769-C11 |
| c081b2e5f59ab109fcc844d4fb68a54d | 0 | 20 | 0 | Eukaryota | Metazoa | Arthropoda | Insecta | Diptera | Cecidomyiidae |  | Cecidomyiidae sp. BIOUG20893-G01 |
| 1eee3338b6d31c85cb66ffac57b68f7c | 0 | 9 | 0 | Eukaryota | Metazoa | Arthropoda | Insecta | Diptera | Cecidomyiidae |  | Cecidomyiidae sp. BOLD:ACI6109 |
| 06c2426cae7ad3ae4d0fc770eaf1d7b3 | 0 | 0 | 6 | Eukaryota | Metazoa | Annelida | Polychaeta | Phyllodocida | Polynoidae |  | cf. Polynoidae sp. DH-2009 |
| c11da9bd886c7b94e90e6e53e1696826 | 0 | 0 | 29 | Eukaryota | Metazoa | Annelida | Polychaeta | Phyllodocida | Polynoidae |  | cf. Polynoidae sp. DH-2009 |
| 06bbc8763f28d54fa99a3b40b608c69f | 0 | 24 | 169 | Eukaryota | Metazoa | Annelida | Polychaeta | Terebellida |  |  | cf. Terebellida sp. DH-2009 |
| 3325153b1618fdca2bc68db6461b4672 | 0 | 0 | 51 | Eukaryota | Metazoa | Annelida | Polychaeta | Terebellida |  |  | cf. Terebellida sp. DH-2009 |
| 74f079fdb77e0b4c12995db488d5085e | 0 | 0 | 0 | Eukaryota | Metazoa | Arthropoda | Insecta | Diptera | Chamaemyiidae |  | Chamaemyiidae sp. BOLD:ACG2991 |
| 8c0953af4c7c2cdf5804561fab138af9 | 0 | 0 | 11 | Eukaryota | Metazoa | Arthropoda | Insecta | Lepidoptera | Gelechiidae | Chionodes | Chionodes fuscomaculella |
| 3a0018caf61a860e36d81efe4e4c8e8f | 9 | 204 | 0 | Eukaryota | Metazoa | Annelida | Polychaeta | Spionida | Cirratulidae | Cirratulus | Cirratulus balaenophilus |
| e487b48222433918829cf2c3c62602d8 | 0 | 0 | 8 | Eukaryota | Metazoa | Annelida | Polychaeta | Spionida | Cirratulidae | Cirratulus | Cirratulus balaenophilus |
| 0ae114a845b8f4e424cdd1d067cf46b0 | 0 | 32 | 0 | Eukaryota | Metazoa | Arthropoda | Insecta | Diptera | Pipunculidae | Clistoabdominalis | Clistoabdominalis digitatus |
| 117d4a205023aa705fedbb282b710da9 | 0 | 7 | 0 | Eukaryota | Metazoa | Arthropoda | Insecta | Diptera | Pipunculidae | Clistoabdominalis | Clistoabdominalis digitatus |
| 117df6f4d5cb03c38edcef2023c47a2e | 0 | 18 | 0 | Eukaryota | Metazoa | Arthropoda | Insecta | Diptera | Pipunculidae | Clistoabdominalis | Clistoabdominalis digitatus |
| 1214c13977f03e71b687d44dd8490ac2 | 0 | 4 | 0 | Eukaryota | Metazoa | Arthropoda | Insecta | Diptera | Pipunculidae | Clistoabdominalis | Clistoabdominalis digitatus |
| 143641fa87b430fdb5ae284e6801a1e2 | 0 | 20 | 0 | Eukaryota | Metazoa | Arthropoda | Insecta | Diptera | Pipunculidae | Clistoabdominalis | Clistoabdominalis digitatus |
| 1f0c8d243e9c6d4e13c143015a7c7e27 | 0 | 7 | 0 | Eukaryota | Metazoa | Arthropoda | Insecta | Diptera | Pipunculidae | Clistoabdominalis | Clistoabdominalis digitatus |
| 2b999bd334b7d3d4574b1c487b1f701b | 0 | 5 | 0 | Eukaryota | Metazoa | Arthropoda | Insecta | Diptera | Pipunculidae | Clistoabdominalis | Clistoabdominalis digitatus |
| 2e27351642e5e9746e7fac844ce5ef92 | 0 | 4 | 0 | Eukaryota | Metazoa | Arthropoda | Insecta | Diptera | Pipunculidae | Clistoabdominalis | Clistoabdominalis digitatus |
| 43e5d4af75ed085582fd4a890705457a | 0 | 14 | 0 | Eukaryota | Metazoa | Arthropoda | Insecta | Diptera | Pipunculidae | Clistoabdominalis | Clistoabdominalis digitatus |
| 45c15400a3f38775331b30b85f12155f | 0 | 7 | 0 | Eukaryota | Metazoa | Arthropoda | Insecta | Diptera | Pipunculidae | Clistoabdominalis | Clistoabdominalis digitatus |
| 45c5e6d4274b1e05782af82b30e4216e | 0 | 5 | 0 | Eukaryota | Metazoa | Arthropoda | Insecta | Diptera | Pipunculidae | istoabdominalis | Clistoabdominalis digitatus |
| 510e62b17ba5959ccfc603968baba4fa | 0 | 6 | 0 | Eukaryota | Metazoa | Arthropoda | Insecta | Diptera | Pipunculidae | Clistoabdominalis | Clistoabdominalis digitatus |
| 5941f34c0ff71498ea1b4b8034f2a003 | 0 | 4 | 0 | Eukaryota | Metazoa | Arthropoda | Insecta | Diptera | Pipunculidae | Clistoabdominalis | Clistoabdominalis digitatus |
| 72b0ed8d4cddafe447d01569531fb54e | 0 | 14 | 0 | Eukaryota | Metazoa | Arthropoda | Insecta | Diptera | Pipunculidae | Clistoabdominalis | Clistoabdominalis digitatus |
| 7f30dca592a2439a16d599fe4674368d | 0 | 31 | 0 | Eukaryota | Metazoa | Arthropoda | Insecta | Diptera | Pipunculidae | Clistoabdominalis | Clistoabdominalis digitatus |
| 8107afe43dd932d51973d670622fd93e | 0 | 7 | 0 | Eukaryota | Metazoa | Arthropoda | Insecta | Diptera | Pipunculidae | Clistoabdominalis | Clistoabdominalis digitatus |
| 8424cccd50aa8031a384026410d215bb | 0 | 8 | 0 | Eukaryota | Metazoa | Arthropoda | Insecta | Diptera | Pipunculidae | Clistoabdominalis | Clistoabdominalis digitatus |
| a68e343aadc595b54a5a22355b3f2aa5 | 0 | 12 | 0 | Eukaryota | Metazoa | Arthropoda | Insecta | Diptera | Pipunculidae | Clistoabdominalis | Clistoabdominalis digitatus |
| a936ab4feee4fb457db6f9be534c0ba7 | 0 | 8 | 0 | Eukaryota | Metazoa | Arthropoda | Insecta | Diptera | Pipunculidae | Clistoabdominalis | Clistoabdominalis digitatus |
| a97f306a169d825b19e9c8cf9bc211f6 | 0 | 11 | 0 | Eukaryota | Metazoa | Arthropoda | Insecta | Diptera | Pipunculidae | Clistoabdominalis | Clistoabdominalis digitatus |
| b8ee2964272696231e8d4fde685b41de | 0 | 16 | 0 | Eukaryota | Metazoa | Arthropoda | Insecta | Diptera | Pipunculidae | Clistoabdominalis | Clistoabdominalis digitatus |
| bc5413a67107ec57341162bd3d8472d9 | 0 | 8 | 0 | Eukaryota | Metazoa | Arthropoda | Insecta | Diptera | Pipunculidae | Clistoabdominalis | Clistoabdominalis digitatus |
| bf1ef7e9289e9e9ae2083a3d88b32e09 | 0 | 16 | 0 | Eukaryota | Metazoa | Arthropoda | Insecta | Diptera | Pipunculidae | Clistoabdominalis | Clistoabdominalis digitatus |
| c0a1eb4811f31c13a805e0005512573b | 0 | 29 | 0 | Eukaryota | Metazoa | Arthropoda | Insecta | Diptera | Pipunculidae | Clistoabdominalis | Clistoabdominalis digitatus |
| c3510580ca801f46738ee4961811bb60 | 0 | 4 | 0 | Eukaryota | Metazoa | Arthropoda | Insecta | Diptera | Pipunculidae | Clistoabdominalis | Clistoabdominalis digitatus |
| d605ec6128565a6c19f10cd7da545436 | 0 | 4 | 0 | Eukaryota | Metazoa | Arthropoda | Insecta | Diptera | Pipunculidae | Clistoabdominalis | Clistoabdominalis digitatus |
| e40117b39922f4d6c06d8c4d5d967ba4 | 0 | 4 | 0 | Eukaryota | Metazoa | Arthropoda | Insecta | Diptera | Pipunculidae | Clistoabdominalis | Clistoabdominalis digitatus |
| e72b8a0f9ca445360ed6aaaedc4dc0f4 | 0 | 8 | 0 | Eukaryota | Metazoa | Arthropoda | Insecta | Diptera | Pipunculidae | Clistoabdominalis | Clistoabdominalis digitatus |
| f33bf11cb4c508d93a10dd86b70b84c8 | 0 | 23 | 19 | Eukaryota | Metazoa | Echinodermata | Crinoidea |  |  |  | Crinoidea sp. NHM_008 |
| 309a7c9a434caa70385fe8a9915b5083 | 9 | 0 | 0 | Eukaryota | Metazoa | Arthropoda | Malacostraca | Isopoda | Cymothoidae | Cymothoa | Cymothoa truncata |
| ab5a874162c0f599a8ea89749b1cde6e | 0 | 0 | 15 | Eukaryota | Metazoa | Chordata | Mammalia | Chiroptera | Molossidae | Cynomops | Cynomops paranus |
| 49cf8aeacba37bb33e6ead1dc603d692 | 649 | 547 | 923 | Eukaryota | Metazoa | Chordata | Aves | Passeriformes | Certhiidae | Cyphorhinus | Cyphorhinus arada |
| 317cdf8dfa2a5d8ce8c44528a6afb706 | 0 | 10 | 0 | Eukaryota | Metazoa | Chordata | Actinopteri | Blenniiformes | Dactyloscopidae | Dactyloscopus | Dactyloscopus tridigitatus |
| 16bcba5a277b434374fe482f2c23a5d2 | 0 | 3 | 6 | Eukaryota | Metazoa | Chordata | Actinopteri |  | Pomacentridae | Dascyllus | Dascyllus aruanus |
| 929391012990605acb06b6a916f3ee06 | 0 | 0 | 21 | Eukaryota | Metazoa | Arthropoda | Insecta | Orthoptera | Acrididae | Diabolocatantops | Diabolocatantops sp. BOLD:AAW5355 |
| 89f033ee291579b1638addda5c72a270 | 10 | 0 | 0 | Eukaryota | Metazoa | Chordata | Actinopteri | Lophiiformes | Ogcocephalidae | Dibranchus | Dibranchus atlanticus |
| 77ff31f998df974c6997adbc92f5b357 | 0 | 0 | 0 | Eukaryota | Metazoa | Mollusca | Gastropoda | Architaenioglossa | Diplommatinidae | Diplommatina | Diplommatina plecta |
| 60ce93d178c12a614a597907c81a43ec | 42 | 90 | 5 | Eukaryota | Metazoa | Arthropoda | Insecta | Trichoptera | Dipseudopsidae | Dipseudopsis | Dipseudopsis africana |
| 0cdad98ee2d166a8efd12c5a4abc8d87 | 0 | 3 | 0 | Eukaryota | Metazoa | Arthropoda | Insecta | Psocoptera | Psyllipsocidae | Dorypteryx | Dorypteryx domestica |
| e03b0b8a709bb39c984e08961f5c9e5b | 0 | 0 | 12 | Eukaryota | Metazoa | Arthropoda | Insecta | Hymenoptera | Dryinidae |  | Dryinidae sp. BOLD:ACL6033 |
| 582ef505d1ae1c1fab5f7b4cf382c648 | 0 | 13 | 0 | Eukaryota | Metazoa | Platyhelminthes | Rhabditophora | Tricladida | Dugesiidae | Dugesia | Dugesia naiadis |
| b73cfe920f532e119c11da35f8862005 | 0 | 0 | 0 | Eukaryota | Metazoa | Arthropoda | Arachnida | Araneae | Dysderidae | Dysdera | Dysdera calderensis |
| e5512dae70ecbee0113c00d8eeb2a175 | 0 | 0 | 0 | Eukaryota | Metazoa | Annelida | Clitellata | Haplotaxida | Enchytraeidae |  | Enchytraeidae sp. En_442 |
| 8bddb9f4a531784b33b45a6108138968 | 0 | 0 | 4 | Eukaryota | Metazoa | Bryozoa | Gymnolaemata | Cheilostomatida | Romancheinidae | Escharella | Escharella immersa |
| fdd4dd3f60036cd206dd318efdd2aa14 | 0 | 0 | 0 | Eukaryota | Metazoa | Annelida | Polychaeta | Phyllodocida | Phyllodocidae | Eumida | Eumida kelaino |
| 044389ec59b8f3360ba227e1230a7d7a | 0 | 0 | 25 | Eukaryota | Metazoa | Arthropoda | Malacostraca | Isopoda | Cirolanidae | Excirolana | Excirolana braziliensis |
| f266f698c8850cf0698507a6e1b1cb38 | 0 | 0 | 0 | Eukaryota | Metazoa | Arthropoda | Malacostraca | Isopoda | Cirolanidae | Excirolana | Excirolana braziliensis |
| ad3b7cf5687545a3e090d9b4209bda37 | 4 | 42 | 3 | Eukaryota | Metazoa | Mollusca | Gastropoda |  |  |  | Gastropoda sp. IOP_0604 |
| be5f1c452cad8ee2569505d2cce8442b | 430 | 192 | 52 | Eukaryota | Metazoa | Arthropoda | Insecta | Phthiraptera | Trichodectidae | Geomydoecus | Geomydoecus perotensis |
| c18fea0380bed2d81f31205aabec4858 | 4 | 0 | 0 | Eukaryota | Metazoa | Arthropoda | Insecta | Phthiraptera | Trichodectidae | Geomydoecus | Geomydoecus perotensis |
| bcb2f5add3f33b5fdb968d5aab4190e0 | 44 | 20 | 2 | Eukaryota | Metazoa | Chordata | Aves | Strigiformes | Strigidae | Glaucidium | Glaucidium passerinum |
| 9d475f617b01c1789e123aef3ec47b39 | 5 | 0 | 0 | Eukaryota | Metazoa | Chordata | Aves | Strigiformes | Strigidae | Glaucidium | Glaucidium tephronotum |
| 9c8e2787e172fd0a9777b1dffeef3f21 | 0 | 80 | 404 | Eukaryota | Metazoa | Chordata | Aves | Passeriformes | Formicariidae | Grallaria | Grallaria guatimalensis |
| 37cc0ca7efd917e0c2c3e7db217b4c8d | 0 | 0 | 0 | Eukaryota | Metazoa | Platyhelminthes | Monogenea | Gyrodactylidea | Gyrodactylidae | Gyrodactylus | Gyrodactylus pannonicus |
| 2d230758d5bb1bba16bea21f706c6f9a | 14 | 0 | 11 | Eukaryota | Metazoa | Arthropoda | Insecta | Diptera | Muscidae | Hebecnema | Hebecnema sp. BOLD-2016 |
| 1ba81e000e8b38ea9981469cb56cf318 | 0 | 0 | 0 | Eukaryota | Metazoa | Arthropoda | Insecta | Hymenoptera | Braconidae | Heterospilus | Heterospilus sp. BOLD:AAI1758 |
| 607918e5b817cb68ec75ca5ea19d4478 | 0 | 7 | 0 | Eukaryota | Metazoa | Arthropoda | Insecta | Hymenoptera | Braconidae | Heterospilus | Heterospilus sp. BOLD:AAI1758 |
| df781ae77319a6028c6a640206fafacf | 111 | 0 | 22 | Eukaryota | Metazoa | Arthropoda | Insecta | Hymenoptera | Braconidae | Heterospilus | Heterospilus sp. BOLD:AAI1758 |
| 021d52677f7a94c81661b17993822bf2 | 0 | 0 | 0 | Eukaryota | Metazoa | Arthropoda | Insecta | Hemiptera | Membracidae | Hypsauchenia | Hypsauchenia sp. CPL-2004 |
| bfbe8bc2d864cf0517d2bcb9e43811a7 | 23 | 0 | 11 | Eukaryota | Metazoa | Arthropoda | Arachnida | Ixodida | Ixodidae | Ixodes | Ixodes scapularis |
| d8e267e1037c0635c45f43502f03e93f | 0 | 0 | 8 | Eukaryota | Metazoa | Arthropoda | Arachnida | Ixodida | Ixodidae | Ixodes | Ixodes scapularis |
| 6c5d58a2bced961168e804015e94471b | 11 | 0 | 0 | Eukaryota | Metazoa | Chordata | Actinopteri |  | Sciaenidae | Johnius | Johnius carouna |
| 0132104f19475a35ba6cc5d07398ae40 | 32 | 0 | 0 | Eukaryota | Metazoa | Arthropoda | Insecta | Hemiptera | Tropiduchidae | Kallitaxila | Kallitaxila sinica |
| c1e05e4bbe69b2ff2641abc536b1a204 | 29 | 0 | 0 | Eukaryota | Metazoa | Chordata | Actinopteri | Atheriniformes | Atherinopsidae | Labidesthes | Labidesthes sicculus |
| 1e55132eff8cc2084a780b839748b4a1 | 11 | 0 | 0 | Eukaryota | Metazoa | Arthropoda | Arachnida | Mesostigmata | Laelapidae |  | Laelapidae sp. BOLD:ACI9030 |
| 02d876aa7e08f972c661231541e72697 | 0 | 88 | 0 | Eukaryota | Metazoa | Arthropoda | Malacostraca | Isopoda | Ligiidae | Ligia | Ligia pallasii |
| 54c8e3d20650082b3209fbcec791e657 | 0 | 54 | 0 | Eukaryota | Metazoa | Arthropoda | Chilopoda | Lithobiomorpha | Lithobiidae | Lithobius | Lithobius sp. BOLD:AAH6437 |
| 9a10f332bd01fada543aa2436aa39deb | 0 | 41 | 75 | Eukaryota | Metazoa | Cnidaria | Scyphozoa | Rhizostomeae | Lobonematidae |  | Lobonematidae sp. 4 LGD-2017 |
| 105e92529e0694d7d0b17673db6d4680 | 0 | 159 | 0 | Eukaryota | Metazoa | Arthropoda | Arachnida | Araneae | Ctenidae | Macroctenus | Macroctenus cf. kingsleyi MRAC 238214 DNA N0 |
| 27aa6b4f92976a30b52c384ad38727e2 | 54 | 663 | 2043 | Eukaryota | Metazoa | Arthropoda | Arachnida | Araneae | Ctenidae | Macroctenus | Macroctenus cf. kingsleyi MRAC 238214 DNA N0 |
| 45b75a8416c49d9dfbc892a54037512b | 0 | 101 | 0 | Eukaryota | Metazoa | Arthropoda | Arachnida | Araneae | Ctenidae | Macroctenus | Macroctenus cf. kingsleyi MRAC 238214 DNA N0 |
| 47cb763a41b81b531a1e78e1475ce4b3 | 0 | 48 | 0 | Eukaryota | Metazoa | Arthropoda | Arachnida | Araneae | Ctenidae | Macroctenus | Macroctenus cf. kingsleyi MRAC 238214 DNA N0 |
| eb52dc3498e6a5a191e02e3087a39f93 | 0 | 51 | 0 | Eukaryota | Metazoa | Arthropoda | Arachnida | Araneae | Ctenidae | Macroctenus | Macroctenus cf. kingsleyi MRAC 238214 DNA N0 |
| f94bd6f98867d7489a2732e066b6eb09 | 50 | 756 | 2981 | Eukaryota | Metazoa | Arthropoda | Arachnida | Araneae | Ctenidae | Macroctenus | Macroctenus cf. kingsleyi MRAC 238214 DNA N0 |
| 51ddfb64ae380382f27ab7e77294120a | 66 | 101 | 15 | Eukaryota | Metazoa | Arthropoda | Malacostraca | Amphipoda | Stenothoidae | Metopa | Metopa boeckii |
| 49d9a2992ea8f128efc31f081f04fb73 | 0 | 8 | 0 | Eukaryota | Metazoa | Arthropoda | Hexanauplia | Calanoida | Metridinidae | Metridia | Metridia gerlachei |
| bcce511319c7fa5700b921279c7759c2 | 0 | 66 | 0 | Eukaryota | Metazoa | Arthropoda | Hexanauplia | Calanoida | Metridinidae | Metridia | Metridia gerlachei |
| 48e6e5fb0486957c2a247fed88ebe152 | 0 | 0 | 0 | Eukaryota | Metazoa | Mollusca | Bivalvia | Pectinoida | Pectinidae | Mimachlamys | Mimachlamys nobilis |
| 69d698a262033d8cd4bd0b538eb17913 | 4 | 0 | 0 | Eukaryota | Metazoa | Ctenophora | Tentaculata | Lobata | Bolinopsidae | Mnemiopsis | Mnemiopsis leidyi |
| 7ce2a1de0479b954fa615c1c166a9ee9 | 3 | 61 | 32 | Eukaryota | Metazoa | Chordata | Mammalia | Rodentia | Muridae | Mus | Mus musculus |
| ad924262a050e757f4b5ecf4740eb42b | 0 | 0 | 0 | Eukaryota | Metazoa | Mollusca | Gastropoda | Neogastropoda | Terebridae |  | Myurella sp. NP-2009 |
| 038162c435c90d36837c481ae1b8ced1 | 0 | 0 | 0 | Eukaryota | Metazoa | Arthropoda | Insecta | Hemiptera | Aphididae | Neoamphorophora | Neoamphorophora kalmiae |
| c2cac860828c95a83e718fb6c3083a48 | 44 | 0 | 0 | Eukaryota | Metazoa | Chordata | Actinopteri | Cypriniformes | Cyprinidae | Notropis | Notropis baileyi |
| 982b2323f9ce221cdd9fe306ca94221d | 9 | 0 | 0 | Eukaryota | Metazoa | Echinodermata | Asteroidea | Valvatida | Odontasteridae | Odontaster | Odontaster validus |
| 623b4c18087252161e87aa6b3b231266 | 14 | 0 | 0 | Eukaryota | Metazoa | Chordata | Aves | Columbiformes | Columbidae | Oena | Oena capensis |
| 3f059d653b1dc71a0fea8c76a069dc1a | 0 | 8 | 0 | Eukaryota | Metazoa | Arthropoda | Collembola | Poduromorpha | Onychiuridae |  | Onychiuridae sp. DPCOL101273 |
| 4226040095340332184ab37b21930bb8 | 0 | 60 | 0 | Eukaryota | Metazoa | Arthropoda | Collembola | Poduromorpha | Onychiuridae |  | Onychiuridae sp. DPCOL101273 |
| 8286e0f2a630c5064918b2e5d340c13a | 0 | 14 | 0 | Eukaryota | Metazoa | Arthropoda | Collembola | Poduromorpha | Onychiuridae |  | Onychiuridae sp. DPCOL101273 |
| 87ca2999061d05cc34833a57fdfa948f | 0 | 11 | 0 | Eukaryota | Metazoa | Arthropoda | Collembola | Poduromorpha | Onychiuridae |  | Onychiuridae sp. DPCOL101273 |
| 27c47cf0f6454548b8b61ca71b1fda10 | 0 | 555 | 0 | Eukaryota | Metazoa | Echinodermata | Ophiuroidea | Ophiurida | Ophiuridae | Ophionotus | Ophionotus victoriae |
| 34349a9bd68aad880d06eadd0580adb3 | 0 | 543 | 0 | Eukaryota | Metazoa | Echinodermata | Ophiuroidea | Ophiurida | Ophiuridae | Ophionotus | Ophionotus victoriae |
| aa77bf1c1d8c1c4c54036e2d4a22f17e | 72 | 884 | 251 | Eukaryota | Metazoa | Echinodermata | Ophiuroidea | Ophiurida | Ophiuridae | Ophionotus | Ophionotus victoriae |
| c5ede58e1ba520be3c226d9af3fe5ae9 | 181 | 254168 | 104 | Eukaryota | Metazoa | Echinodermata | Ophiuroidea | Ophiurida | Ophiuridae | Ophionotus | Ophionotus victoriae |
| e413ba4affa1724c22adfa4a29b6456b | 16 | 0 | 0 | Eukaryota | Metazoa | Echinodermata | Ophiuroidea | Ophiurida | Ophiuridae | Ophionotus | Ophionotus victoriae |
| 08776efb618bab7db91cbbc7bdb8ad60 | 100 | 141 | 82 | Eukaryota | Metazoa | Annelida | Polychaeta | Eunicida | Dorvilleidae | Ophryotrocha | Ophryotrocha clava |
| 1ab21ffedcba2eaf938f38403575331e | 0 | 0 | 0 | Eukaryota | Metazoa | Annelida | Polychaeta | Eunicida | Dorvilleidae | Ophryotrocha | Ophryotrocha clava |
| 1fd6c0b4544074da97257b8ae088c534 | 0 | 0 | 0 | Eukaryota | Metazoa | Annelida | Polychaeta | Eunicida | Dorvilleidae | Ophryotrocha | Ophryotrocha clava |
| 4db6e15518fa36d0f21c5c1b18ab2426 | 0 | 0 | 0 | Eukaryota | Metazoa | Annelida | Polychaeta | Eunicida | Dorvilleidae | Ophryotrocha | Ophryotrocha clava |
| 9b610c0817198248eabdd1a50e3c45eb | 47 | 9 | 34 | Eukaryota | Metazoa | Annelida | Polychaeta | Sabellida | Siboglinidae | Osedax | Osedax sp. 'green-palp' |
| 024f89fd4819918fb10b516fd09de684 | 0 | 0 | 0 | Eukaryota | Metazoa | Arthropoda | Hexanauplia | Calanoida | Acartiidae | Paralabidocera | Paralabidocera grandispina |
| 2978acf0cabd5e5e96373e7b5b1c750d | 0 | 0 | 0 | Eukaryota | Metazoa | Arthropoda | Hexanauplia | Calanoida | Acartiidae | Paralabidocera | Paralabidocera grandispina |
| 99d529220add5867a5b7b5517e24ee0a | 0 | 0 | 0 | Eukaryota | Metazoa | Arthropoda | Hexanauplia | Calanoida | Acartiidae | Paralabidocera | Paralabidocera grandispina |
| b95764f468758182a51b0204150408fd | 0 | 0 | 0 | Eukaryota | Metazoa | Arthropoda | Hexanauplia | Calanoida | Acartiidae | Paralabidocera | Paralabidocera grandispina |
| bbbc12cd212fb39788b8c338ca1df94e | 0 | 0 | 0 | Eukaryota | Metazoa | Arthropoda | Hexanauplia | Calanoida | Acartiidae | Paralabidocera | Paralabidocera grandispina |
| ccd7960a78c504410cd0e608fe25b20a | 0 | 0 | 53 | Eukaryota | Metazoa | Arthropoda | Hexanauplia | Calanoida | Acartiidae | Paralabidocera | Paralabidocera grandispina |
| d69d7d4316c07d66e97321f061f55e96 | 0 | 0 | 439 | Eukaryota | Metazoa | Arthropoda | Hexanauplia | Calanoida | Acartiidae | Paralabidocera | Paralabidocera grandispina |
| e12e67cc1c4bf4aeb1762da979d5a76c | 0 | 0 | 21 | Eukaryota | Metazoa | Arthropoda | Hexanauplia | Calanoida | Acartiidae | Paralabidocera | Paralabidocera grandispina |
| c47af405209be7d7c485ec43577822eb | 45 | 450 | 83 | Eukaryota | Metazoa | Arthropoda | Insecta | Lepidoptera | Geometridae | Patalene | Patalene asychisaria |
| 59474d690d66cc3b4a87bff9f4691d6b | 0 | 154 | 85 | Eukaryota | Metazoa | Chordata | Actinopteri | Pempheriformes | Pempheridae | Pempheris | Pempheris schomburgkii |
| aeec72f6a05529ef56cc2487a59525fb | 0 | 19 | 0 | Eukaryota | Metazoa | Rotifera | Bdelloidea | Philodinida | Philodinidae | Philodina | Philodina megalotrocha |
| 7570ca83d62db5c984a1bba1733e134e | 0 | 54 | 0 | Eukaryota | Metazoa | Arthropoda | Arachnida | Araneae | Pimoidae | Pimoa | Pimoa sp. SP74 |
| 88abc7f2d23fbbe769be3b63a6ed9be4 | 0 | 46 | 0 | Eukaryota | Metazoa | Mollusca | Gastropoda | Architaenioglossa | Ampullariidae | Pomacea | Pomacea flagellata |
| ae36f7dd0ee6e1b35c86180b7ca76ceb | 23 | 0 | 20 | Eukaryota | Metazoa | Mollusca | Gastropoda | Acochlidiacea | Microhedylidae | Pontohedyle | Pontohedyle milaschewitchii |
| f69a438cf6cd050902020ec4c4abd80e | 0 | 6 | 0 | Eukaryota | Metazoa | Arthropoda | Malacostraca | Decapoda | Portunidae | Portunus | Portunus segnis |
| 76e992a5e227ddcbaa7cc2f5934aa31e | 0 | 0 | 0 | Eukaryota | Metazoa | Arthropoda | Malacostraca | Isopoda | Asellidae | Proasellus | Proasellus escolai |
| b6abe091223a3293f5c04959f1b3353b | 4 | 0 | 0 | Eukaryota | Metazoa | Arthropoda | Insecta | Trichoptera | Glossosomatidae | Protoptila | Protoptila voluta |
| d1609c1f386be7fc5fb6a5d6eb3334da | 5 | 0 | 0 | Eukaryota | Metazoa | Arthropoda | Arachnida | Araneae | Pholcidae | Psilochorus | Psilochorus hesperus |
| c744b206edbfcec355a78fbeb6f97339 | 0 | 0 | 0 | Eukaryota | Metazoa | Echinodermata | Holothuroidea | Dendrochirotida | Psolidae | Psolus | Psolus charcoti |
| 34bd79265ba58b213aa4ef9c483b867a | 12 | 0 | 0 | Eukaryota | Metazoa | Arthropoda | Insecta | Hymenoptera | Pteromalidae |  | Pteromalidae sp. BOLD-2016 |
| 8a52dd2dd4e431f95f536ee257fd9d8c | 0 | 0 | 7 | Eukaryota | Metazoa | Mollusca | Gastropoda | Neogastropoda | Costellariidae | Pusia | Pusia ebenus |
| a6e019c4eb9033dc67ddd5297790dadc | 30 | 320 | 0 | Eukaryota | Metazoa | Mollusca | Gastropoda | Neogastropoda | Costellariidae | Pusia | Pusia ebenus |
| c5f4e8a7aa7fc01e9e568d0ee82adab6 | 0 | 19 | 0 | Eukaryota | Metazoa | Kinorhyncha |  | Homalorhagida | Pycnophyidae | Pycnophyes | Pycnophyes flaveolatus |
| 778f60d6e06e2fe92127ca766ba82805 | 0 | 6 | 0 | Eukaryota | Metazoa | Arthropoda | Insecta | Diptera | Sciaridae |  | Sciaridae sp. BOLD:ACL4012 |
| ec6bf28b07a99c9518ec958a5a422135 | 9 | 0 | 0 | Eukaryota | Metazoa | Cnidaria | Hydrozoa | Narcomedusae | Cuninidae | Solmissus | Solmissus marshalli |
| 536a2504453b318a8b3437a969ab3c4e | 13 | 0 | 0 | Eukaryota | Metazoa | Arthropoda | Insecta | Hymenoptera | Braconidae | Spathius | Spathius aff. caudatus 1 AZR-2017 |
| da86e1d9f47ecbee986da0c249c045c2 | 0 | 0 | 0 | Eukaryota | Metazoa | Mollusca | Bivalvia | Arcoida | Arcidae | Tegillarca | Tegillarca nodifera |
| 022ce19250d18ecb26b61c640eda0273 | 20 | 0 | 66 | Eukaryota | Metazoa | Platyhelminthes | Rhabditophora | Rhabdocoela | Temnocephalidae | Temnosewellia | Temnosewellia albata |
| b68d4e4a5b71c2bed0cc29befd999b7c | 19 | 0 | 0 | Eukaryota | Metazoa | Platyhelminthes | Rhabditophora | Rhabdocoela | Temnocephalidae | Temnosewellia | Temnosewellia albata |
| cd0aaf55bf8d95b7d814772728cbcdf9 | 43 | 33 | 0 | Eukaryota | Metazoa | Platyhelminthes | Rhabditophora | Rhabdocoela | Temnocephalidae | Temnosewellia | Temnosewellia albata |
| e05a88e84160abd2e9b6a03c19fe24bb | 0 | 4 | 0 | Eukaryota | Metazoa | Arthropoda | Arachnida | Sarcoptiformes | Terpnacaridae |  | Terpnacaridae sp. BOLD:AAU6267 |
| 2db18cb4bbf8818a455e9fd61873538e | 0 | 0 | 9 | Eukaryota | Metazoa | Chordata | Actinopteri | Labriformes | Labridae | Thalassoma | Thalassoma quinquevittatum |
| ca0fe9710f24c79507aaec76e93f5f37 | 0 | 108 | 0 | Eukaryota | Metazoa | Annelida | Polychaeta | Terebellida | Terebellidae | Thelepus | Thelepus plagiostoma |
| a5dc4cebcfb9a3ff0aaf1a5d96223281 | 24 | 0 | 0 | Eukaryota | Metazoa | Arthropoda | Malacostraca | Decapoda | Hippolytidae | Tozeuma | Tozeuma carolinense |
| d19488b399402afa3f411d812814cb04 | 14 | 0 | 0 | Eukaryota | Metazoa | Arthropoda | Insecta | Coleoptera | Curculionidae | Trigonopterus | Trigonopterus sp. 621 MB-2015 |
| 0e83ce82bdc4c05fdceb584b8c1a4748 | 50 | 0 | 25 | Eukaryota | Metazoa | Mollusca | Bivalvia | Unionoida | Unionidae | Unio | Unio durieui |
| 831fd40b5e226f9ba3e551d843a083fb | 0 | 0 | 0 | Eukaryota | Metazoa | Chordata |  | Squamata | Phrynosomatidae | Uta | Uta stansburiana |
| 03d1d0e4744992cea69c783c23492768 | 6 | 0 | 0 | Eukaryota | Metazoa | Arthropoda | Arachnida | Araneae | Thomisidae | Xysticus | Xysticus bimaculatus |
| 44331df7f8fd1bd2d9de398b6671a6f5 | 0 | 0 | 7 | Eukaryota | Metazoa | Arthropoda | Arachnida | Araneae | Thomisidae | Xysticus | Xysticus durus |
| 51518acfa94d710f3f207cc63bbdd3b3 | 24987 | 27135 | 132795 | Eukaryota | Metazoa | Mollusca | Bivalvia | Nuculanoida | Yoldiidae | Yoldia | Yoldia eightsii |
| 952a48a38980fe174027fbf83a726534 | 0 | 0 | 23 | Eukaryota | Metazoa | Mollusca | Bivalvia | Nuculanoida | Yoldiidae | Yoldia | Yoldia eightsii |
| a247f6317f88869f57f03b49e184019d | 0 | 0 | 0 | Eukaryota | Metazoa | Mollusca | Bivalvia | Nuculanoida | Yoldiidae | Yoldia | Yoldia eightsii |
| e046b3168cca4162df81a8644b558c40 | 0 | 0 | 0 | Eukaryota | Metazoa | Mollusca | Bivalvia | Nuculanoida | Yoldiidae | Yoldia | Yoldia eightsii |
| 03e81e297b6623f4eb0a902ad85dc1e5 | 239 | 177 | 0 | Eukaryota | Metazoa | Annelida | Polychaeta | Phyllodocida | Nephtyidae |  |  |
| 1fcb35f2427dfc4bed9fc254e46bb1ae | 0 | 0 | 0 | Eukaryota | Metazoa | Annelida | Polychaeta | Phyllodocida | Nephtyidae |  |  |
| 39e26f74d65694291cf5576cf2ba2a77 | 0 | 0 | 0 | Eukaryota | Metazoa | Annelida | Polychaeta | Phyllodocida | Nephtyidae |  |  |
| 3d6829010866a65b9a9b23ea063e1bee | 0 | 10 | 0 | Eukaryota | Metazoa | Annelida | Polychaeta | Phyllodocida | Syllidae |  |  |
| 6eaab046f3e2abf830bdc00e5b3754b9 | 3226 | 7629 | 2349 | Eukaryota | Metazoa | Annelida | Polychaeta | Phyllodocida | Nephtyidae |  |  |
| 8b921e48355fa2d3d8865d1e9314fa29 | 0 | 0 | 0 | Eukaryota | Metazoa | Annelida | Polychaeta | Phyllodocida | Nephtyidae |  |  |
| b218ade6615cf431f64549fc2381b750 | 0 | 938 | 0 | Eukaryota | Metazoa | Annelida | Polychaeta | Phyllodocida | Nephtyidae |  |  |
| b62ed6887473a2c005f7755af1e557d1 | 0 | 0 | 0 | Eukaryota | Metazoa | Annelida | Polychaeta | Phyllodocida | Nephtyidae |  |  |
| bd51ab145696c70bea3cd3f51d0ff77e | 0 | 1632 | 0 | Eukaryota | Metazoa | Annelida | Polychaeta | Phyllodocida | Nephtyidae |  |  |
| bfe73aefcfc17b1366fd253f4dff1b5f | 0 | 0 | 0 | Eukaryota | Metazoa | Annelida | Polychaeta | Phyllodocida | Syllidae |  |  |
| c026982b2e750c8180dd9fbf53a2ad80 | 3076 | 3868 | 213 | Eukaryota | Metazoa | Annelida | Polychaeta | Phyllodocida | Nephtyidae |  |  |
| c27ec963a5bddf31574c53a4b762518b | 21633 | 83498 | 5790 | Eukaryota | Metazoa | Annelida | Polychaeta | Phyllodocida | Nephtyidae |  |  |
| ddb8a851dc2fb4d8b1cd2a786dcbcd63 | 0 | 393 | 0 | Eukaryota | Metazoa | Annelida | Polychaeta | Phyllodocida | Nephtyidae |  |  |
| e93b2240ca1a26ddd64935c2a5b6a500 | 0 | 323 | 0 | Eukaryota | Metazoa | Annelida | Polychaeta | Phyllodocida | Nephtyidae |  |  |
| eba1485e59af541ab50db5f2fc056bc1 | 0 | 0 | 0 | Eukaryota | Metazoa | Annelida | Polychaeta | Phyllodocida | Nephtyidae |  |  |
| f498bd369169dd037cc21ab6d2e4ab37 | 251 | 0 | 0 | Eukaryota | Metazoa | Annelida | Polychaeta | Phyllodocida | Nephtyidae |  |  |
| 09b18a9c4fcb593dba0f5d9211c9401f | 0 | 0 | 0 | Eukaryota | Metazoa | Arthropoda | Arachnida | Araneae | Idiopidae | Idiosoma |  |
| 0a328331716814a12e7cc8a01ab66f0b | 0 | 0 | 4 | Eukaryota | Metazoa | Arthropoda | Insecta | Hymenoptera | Ichneumonidae | Sussaba |  |
| 18a2e110004e455b097f83f0c05922c6 | 11 | 0 | 0 | Eukaryota | Metazoa | Arthropoda |  |  |  |  |  |
| 1d08860f1f02226df177b2e0a8b08994 | 74 | 0 | 0 | Eukaryota | Metazoa | Arthropoda | Insecta | Hymenoptera |  |  |  |
| 219a853a0a730d1f261dd01ef137372f | 0 | 0 | 0 | Eukaryota | Metazoa | Arthropoda | Insecta | Hymenoptera |  |  |  |
| 259e93f0bb65d07780a9b5ce5e757bec | 15 | 0 | 0 | Eukaryota | Metazoa | Arthropoda | Insecta | Hymenoptera |  |  |  |
| 2b91e6d72b48a449e4a36a1eb31fd98f | 0 | 0 | 8 | Eukaryota | Metazoa | Arthropoda | Insecta | Odonata | Gomphidae |  |  |
| 4ceafea83b9a28cfb4a0f341c9e25204 | 0 | 0 | 0 | Eukaryota | Metazoa | Arthropoda | Insecta |  |  |  |  |
| 7f8ade63fe041f2b9340d3bd51201fd8 | 3 | 51 | 15 | Eukaryota | Metazoa | Arthropoda | Arachnida | Sarcoptiformes | Eremellidae |  |  |
| 85bdd26a0ff9657d9d3d0f6fee445f9f | 11 | 0 | 0 | Eukaryota | Metazoa | Arthropoda | Insecta |  |  |  |  |
| a21b497bf2bb562fd354e41a49657fb2 | 16 | 9 | 0 | Eukaryota | Metazoa | Arthropoda | Insecta | Hymenoptera | Ichneumonidae | Sussaba |  |
| c2c3f6cfb7fa7b31e94dc12a632819a0 | 0 | 8 | 0 | Eukaryota | Metazoa | Arthropoda | Insecta | Plecoptera | Perlodidae | Perlodes |  |
| c5ab042adabf53629c87aa52668f9d3f | 7 | 0 | 0 | Eukaryota | Metazoa | Arthropoda | Insecta |  |  |  |  |
| db2ae51f2667f1784825677fde8fdff9 | 0 | 9 | 0 | Eukaryota | Metazoa | Arthropoda |  |  |  |  |  |
| ddc4af043f5cf8d8f1df36eade48f712 | 9 | 74 | 6 | Eukaryota | Metazoa | Arthropoda | Arachnida | Mesostigmata | Phytoseiidae |  |  |
| e9a28771acd1eadcbc6e3cbde61b6d97 | 0 | 76 | 969 | Eukaryota | Metazoa | Arthropoda | Arachnida | Araneae | Ctenidae | Macroctenus |  |
| fa059b9b01c9a35a7810e8938588daca | 0 | 0 | 0 | Eukaryota | Metazoa | Arthropoda | Insecta | Hymenoptera | Ichneumonidae | Sussaba |  |
| fe2a798e52b52deb5f4945df43e2da07 | 490 | 136 | 18 | Eukaryota | Metazoa | Arthropoda | Arachnida | Araneae | Idiopidae | Idiosoma |  |
| 46e05651925bb77d8a5dc5398736e827 | 0 | 0 | 0 | Eukaryota | Metazoa | Bryozoa | Gymnolaemata | Cheilostomatida | Watersiporidae | Watersipora |  |
| 0570abfcfec201193b5de68031237381 | 0 | 0 | 0 | Eukaryota | Metazoa | Chordata |  |  |  |  |  |
| 08fb7435697fe5871371c4d08735a66f | 0 | 85 | 0 | Eukaryota | Metazoa | Chordata |  | Squamata |  |  |  |
| 3a17a2da986ef5f9fc4f284ccf62cf0f | 0 | 4098 | 60 | Eukaryota | Metazoa | Chordata |  | Squamata |  |  |  |
| 3af4ceac3db9571c00383925b63140a7 | 0 | 305 | 0 | Eukaryota | Metazoa | Chordata |  | Squamata |  |  |  |
| 41d7aed584a3f627fbaa45301ac5f177 | 0 | 42 | 0 | Eukaryota | Metazoa | Chordata |  | Squamata |  |  |  |
| 4717177bb3a3232378589d5091aefc4d | 0 | 708 | 0 | Eukaryota | Metazoa | Chordata |  | Squamata |  |  |  |
| 57d22f660bfa49aed96abf13802d0113 | 0 | 9 | 0 | Eukaryota | Metazoa | Chordata |  |  |  |  |  |
| 5dcb313708db18bd631c29e23e586fe8 | 0 | 0 | 47 | Eukaryota | Metazoa | Chordata |  |  |  |  |  |
| 5fae74a14078ba92dbaaeaaddec8b856 | 12 | 0 | 0 | Eukaryota | Metazoa | Chordata |  | Squamata | Sphaerodactylidae | Teratoscincus |  |
| 70eead67d43adb04b6ab09ea17eaefa7 | 2 | 5407 | 29 | Eukaryota | Metazoa | Chordata |  | Squamata |  |  |  |
| 7139578b9115bb49978ff089d92945b5 | 0 | 328 | 0 | Eukaryota | Metazoa | Chordata |  | Squamata |  |  |  |
| bf63b34a40703090e7ec34b977fb872c | 0 | 160 | 0 | Eukaryota | Metazoa | Chordata |  | Squamata |  |  |  |
| c50b8986853d82d013a754e78626de99 | 0 | 215 | 0 | Eukaryota | Metazoa | Chordata |  | Squamata |  |  |  |
| c601da78db9491eb1805623d4f5de22c | 0 | 4 | 0 | Eukaryota | Metazoa | Chordata | Actinopteri |  |  |  |  |
| d328665d161445eed285310b40120890 | 8 | 0 | 0 | Eukaryota | Metazoa | Chordata |  |  |  |  |  |
| d55840af22c23bb82eec5190a7c9935d | 0 | 152 | 0 | Eukaryota | Metazoa | Chordata |  | Squamata |  |  |  |
| e2bb1c3a78736e3966829a19ac8a6126 | 0 | 1224 | 0 | Eukaryota | Metazoa | Chordata |  | Squamata |  |  |  |
| e9f6f145c3e8ae3bc51b88dfdf411dbd | 0 | 0 | 0 | Eukaryota | Metazoa | Chordata |  | Squamata | Colubridae |  |  |
| d7cb8a3f34136e0b30cf1b2d30fabe8d | 0 | 0 | 4 | Eukaryota | Metazoa | Cnidaria | Hydrozoa | Leptothecata |  |  |  |
| 8abe7aecc27ad932bb326e57320824f3 | 0 | 0 | 0 | Eukaryota | Metazoa | Echinodermata | Asteroidea | Valvatida | Oreasteridae |  |  |
| f9276215a1b42c931340b956ca4757b3 | 72 | 0 | 10 | Eukaryota | Metazoa | Rotifera | Bdelloidea | Philodinida | Philodinidae | Macrotrachela |  |
| 422fa6d3e6e5854405d3b34c711e6782 | 0 | 24 | 0 | Eukaryota | Metazoa |  |  |  |  |  |  |
| e3c8da75b02e2c934f9bc68fdb324aa5 | 57 | 0 | 323 | Eukaryota | Metazoa |  |  |  |  |  |  |
| bf101dd52daf29ce3bc230671ae9f3b2 | 0 | 0 | 5 | Eukaryota | Metazoa |  |  |  |  |  |  |
| e43dbc9268ae5ea4f77eb288a30327c6 | 0 | 267 | 0 | Eukaryota | Metazoa |  |  |  |  |  |  |
| b4e71ecd9e13a1ad8ac24a07bd359be8 | 0 | 0 | 8 | Eukaryota | Metazoa |  |  |  |  |  |  |

**Table S5.** eDNA species list retrieved using the COI marker using a 97% sequence BLAST match against GenBank database. First column shows ASVs identification tag, followed by number of reads allocated to each ASVs per sample site (HC1, HC2, HC3) and the last seven columns show the taxonomy ranking to each ASVs from Kingdom to species, respectively. Hangar Cove replicates 1-3 (HC1, HC2, HC3) and South Cove replicates 1-3 (SC1-3).
